# Energetically equivalent structural transitions in the Rad17–Rad9–Hus1–Rad1–Rhino complex underlie the sequential progression from activation through maintenance to inactivation of the ATR-dependent DNA damage response

**DOI:** 10.1093/nar/gkag093

**Published:** 2026-02-16

**Authors:** Yasunori Fukumoto, Ryuzaburo Yuki, Yasumitsu Ogra

**Affiliations:** Laboratory of Toxicology and Environmental Health, Graduate School of Pharmaceutical Sciences, Chiba University, Chiba 260-8675, Japan; Laboratory of Biochemistry & Molecular Biology, Kyoto Pharmaceutical University, Kyoto 607-8414, Japan; Laboratory of Toxicology and Environmental Health, Graduate School of Pharmaceutical Sciences, Chiba University, Chiba 260-8675, Japan

## Abstract

Activation of the ATR-dependent DNA damage response (ATR-DDR) is well characterized; however, the molecular mechanisms underlying its maintenance and inactivation remain largely elusive. Rhino is the least understood component of ATR-DDR. Structural modeling and binding free energy calculations revealed structural remodeling involving Rad17, Rad9–Hus1–Rad1 (9–1–1), and Rhino during ATR-DDR progression. Biochemical and computational analyses revealed the competitive binding of Rad17 and Rhino to the 9–1–1 complex, suggesting a structural transition from the Rad17–9–1–1 complex to the Rhino–9–1–1 complex. The presence of two conserved KYxxL+ motifs in Rhino suggests that it bridges the two 9–1–1 complexes. This enables the polymerization of multiple 9–1–1 complexes through Rhino and explains the long-standing discrepancy between the conventional model and experimental observations of Rad17 and Rad9 foci. Furthermore, structural analysis of the Rad9 C-terminal tail revealed its ability to compete with both Rhino and Rad17, leading to disassembly of the checkpoint complex and providing a mechanism for checkpoint inactivation. Quantum chemical calculations revealed comparable binding free energies for intermediate complexes. These observations suggest that the Rad17–9–1–1–Rhino complex undergoes energetically equivalent structural transitions, providing a mechanistic basis for the sequential progression of ATR-DDR.

## Graphical Abstract

**Graphical Abstract:**
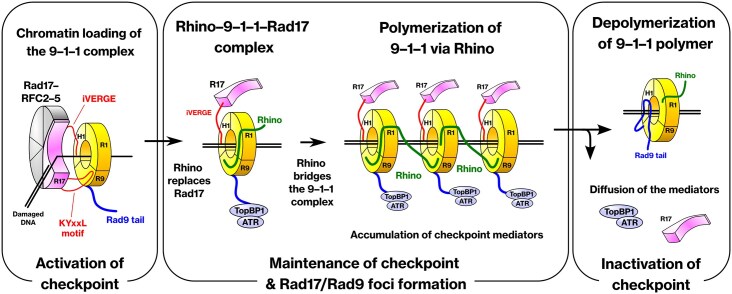

## Introduction

The DNA damage response (DDR) is involved in maintaining genome stability under homeostatic conditions and contributes to resistance to chemotherapy and radiotherapy in cancer cells. The ATR-dependent DDR (ATR-DDR) is a major regulator of DNA damage checkpoints, arresting cell cycle progression and allowing time for DNA repair. In ATR-DDR, various DNA modifications are processed via distinct mechanisms and converted into single-stranded gaps, serving as the initial triggers for ATR activation [1, 2]. While the activation of ATR-DDR has been explored, the molecular mechanisms underlying its transition from activation to maintenance and inactivation remain to be elucidated.

Regulation of the Rad17–RFC2–5 and Rad9–Hus1–Rad1 (9–1–1) complexes is a central issue in ATR-DDR. Rad17 has sequence similarity with the replication factor C (RFC) [3] and comprises an RFC-like complex wherein RFC1 is replaced by Rad17 [4, 5]. This Rad17–RFC2–5 complex recognizes damaged DNA through the direct interaction of Rad17 with the junction of single- and double-stranded DNA, where the 5′-end is recessed [5–9]. The 9–1–1 complex is a PCNA-like heterotrimer [10, 11] loaded onto damaged chromatin by the Rad17–RFC2–5 complex [12–14]. The 9–1–1 complex serves as a scaffold that tethers other DDR factors to activate downstream responses in ATR-DDR. A major factor in this process is the Rad9 tail, an intrinsically disordered region in the C-terminus of Rad9 that protrudes from the core-ring structure of the 9–1–1 complex [15–17]. Another factor is the hydrophobic pocket on the front side of the Rad9 core-ring structure [11]. The molecular functions of Rad17 and 9–1–1 complex are well illustrated in the activation phase, but remain to be elucidated in subsequent phases.

Our previous biochemical analyses identified the Rad17 KYxxL motif as the first conserved motif essential for interaction with the 9–1–1 complex [18]. Later structural studies showed that the KYxxL motif engaged the Rad9 front pocket [5, 7]. In our *in silico* analyses, most of the interaction energy was attributed to Rad17, especially the KYxxL motif, whereas the RFC2–5 subunits showed almost no binding energy [19]. Rad17 contains two additional domains that interact with the 9–1–1 complex. One is Rad17 T16–D24 residues, which interacts with Rad1 [20]. The other is the C-terminal acidic sequence termed iVERGE, which is essential for interacting with the 9–1–1 complex in a manner dependent on its phosphorylation by CK2 and CK1 [19, 21–23]. Our *in silico* and biochemical analyses suggest an association between iVERGE and Hus1 [19]. In *Saccharomyces cerevisiae*, the C-terminal peptide of a Rad17 homolog (RAD24) interacts with a Hus1 homolog (MEC3) in an analogous manner [6]. A static interaction between Rad17 and the 9–1–1 complex has been illustrated; however, little is known about its dynamics and dissociation in the subsequent stages after chromatin loading of the 9–1–1 complex.

Rhino (RAD9, HUS1, RAD1-interacting nuclear orphan) is one of the least characterized members of the ATR-DDR family. Rhino interacts with the 9–1–1 complex and TopBP1 and is required for ATR-dependent Chk1 phosphorylation [24, 25]. The interaction with the 9–1–1 complex was attributed to Rhino S55–F61 residues, which interact with the hydrophobic cleft on the outer side of Rad1 [24, 26]. However, the molecular mechanisms underlying the involvement of Rhino in ATR-DDR remain unclear.

Previous work reported competitive binding of Rhino W56–A64 residues and Rad17 T16–D24 residues to Rad1 [20]; however, the binding energy of Rad17 T16–D24 to Rad1 was approximately −300 kJ/mol, whereas the Rad17 KYxxL motif and the associated peptide showed a binding energy of approximately −1300 kJ/mol with Rad9 [19]. If Rhino competes with Rad17 to bind to the 9–1–1 complex, it must compete with the Rad17 KYxxL motif in the Rad9 interaction.

Here, we investigated the binding conformation of Rhino on the 9–1–1 complex using biochemical and computational approaches to examine the competitive binding of Rhino and Rad17. *In silico* analyses were conducted to characterize the structural and energetic features of the binding conformations of Rhino K13–F18, K90–F96, the Rad9 C-terminal tail, and p21 in the Rad9 front pocket. These analyses aimed to identify a conserved motif, the KYxxL+ motif, which contributes to the polymerization of the 9–1–1 complex and checkpoint inactivation. Collectively, these efforts were intended to explore energetically equivalent remodeling within the Rad17–Rad9–Hus1–Rad1–Rhino complex that underlies the progression of ATR-DDR from activation through maintenance to inactivation.

## Materials and methods

### Structure prediction by AlphaFold2

The structures of protein complexes composed of the core-ring structure of the 9–1–1 complex and various ligand peptides were predicted using AlphaFold v2.3.1 [27] with the AlphaFold-Multimer implementation [28]. All five preset models were employed, and five conformations were generated from each model. Among the 25 predicted structures, the one with the highest confidence score (ipTM + pTM) was selected as the best model and used as the initial structure for subsequent conformational sampling via molecular dynamics simulations. The ipTM + pTM scores for each AlphaFold2 prediction are listed in Supplementary Table S1.

### Simulated annealing molecular dynamics simulation

Possible binding conformations of ligand peptides on the core-ring structure of the 9–1–1 complex were sampled using simulated annealing molecular dynamics (SA-MD), as described previously [19]. The initial protein complex was protonated using the H++ server [29]. tLEaP was used to restore a disulfide bond between Rad1 C58 and C272 and to generate topology files using the Amber ff19SB force field [30]. The protein complex was placed in a truncated octahedral box with at least 12 Å between the protein surface and the box edges. The simulation box was filled with the OPC water model and neutralized by adding Na^+^ or Cl^−^ ions. Coordinate and topology files were converted to GROMACS format using ParmEd [31] from AmberTools [32].

MD simulations were performed using GROMACS 2024.1 [33], compiled with CUDA 12.4. The geometry of the initial structure was optimized using the steepest descent method with an energy convergence threshold of 1000 kJ/mol·nm. Subsequent simulations employed the leapfrog integration algorithm. Bond lengths were constrained using the LINCS algorithm [34]. The Verlet scheme was used to generate neighbor lists, and a cutoff of 12 Å was applied to van der Waals and short-range electrostatic interactions. Long-range electrostatics were treated using the particle-mesh Ewald method [35]. The system temperature was maintained at 300 K using the modified Berendsen thermostat, with two temperature coupling groups: one for the proteins and one for water and ions. The pressure was maintained at 100 kPa using the C-rescale barostat during NPT equilibration, and the Parrinello–Rahman barostat was used during the production simulations. Heavy atoms of proteins were restrained during NVT and NPT equilibration phases. Subsequently, the position restraints were removed, and the production MD simulation was performed using the simulated annealing (SA) algorithm.

The SA algorithm was implemented with a time step of 1 fs, and production runs were performed for 25, 50, or 100 ns, as described previously [19]. Two temperature-coupling groups were defined: a high-temperature group (ligand peptides) and a low-temperature group (the core ring structure of the 9–1–1 complex). The high-temperature coupling profile was as follows: the system was linearly heated from 300 K to 1000 K over 0–500 ps, maintained at 1000 K during 500–1000 ps to promote conformational transitions, then cooled linearly back to 300 K over 1000–2000 ps, followed by equilibration at 300 K during 2000–2500 ps. The low-temperature group was cycled between 300 K and 350 K in synchronization with the high-temperature group, as shown in Supplementary Fig. S1A. This cycle was repeated 10, 20, or 40 times over the course of 25, 50, or 100 ns, depending on the simulation purpose (Supplementary Fig. S1B). Trajectories were analyzed using the TRJCONF command, and averaged structures were generated from each 300 K equilibration phase using the RMS command. These averaged structures were energy-minimized using GROMACS with the steepest descent algorithm, applying a convergence threshold of 1000 kJ/mol·nm, and used as input for the subsequent fragment molecular orbital calculation.

Root-mean-square fluctuation (RMSF) was calculated using the RMSF command of GROMACS. The RMSF for each residue was calculated based on the trajectory segments corresponding to the preceding cooling, equilibration, and subsequent heating steps (Supplementary Fig. S1A). RMSF values were averaged across all conformations within each cluster and presented as mean ± standard deviation.

### Pair-interaction energy calculation using the fragment molecular orbital method

Pair-interaction energy (PIE), also referred to as the inter-fragment interaction energy, was calculated for structures obtained by averaging over SA-MD trajectories and subsequently energy-minimized. The interaction between each residue of the ligand and receptor was analyzed by calculating PIE (Supplementary Fig. S1C and D). PIE was calculated using the fragment molecular orbital (FMO) method [36, 37] implemented in GAMESS [38, 39].

The calculations were performed as described previously [19, 40]. Input files for GAMESS were generated using Facio 23.1.5 [41]. Each polypeptide was divided into single-residue fragments (Supplementary Fig. S1C). PIE was calculated using GAMESS 2023.R2 [38, 39], dynamically linked to Intel oneAPI Math Kernel Library. The molecular orbitals of the fragments were calculated using the third-generation density functional tight-binding (DFTB3) method combined with the conductor-like polarizable continuum model (PCM) [42]. The DFTB3 calculation used the 3OB-3-1 parameter set [43, 44]. Dispersion interactions were corrected using the third-generation implementation of Grimme’s empirical dispersion correction, DFT-D3(BJ) [45]. PIE between each residue of the ligand peptide and the core-ring structure of the 9–1–1 complex was calculated using two-body expansion (FMO2) of the FMO method with DFTB3 and PCM (FMO2-DFTB3/PCM) [38], along with the PIE decomposition analysis (PIEDA) [46]. The resulting PIE lists were used for subsequent analyses.

### Identification of stable conformations sampled in the SA-MD simulations

Stable conformations of ligand peptides bound to the receptor were identified by analyzing the PIE matrices, as previously described with modifications [19]. Using PIE values as feature vectors, similar conformations were grouped using agglomerative hierarchical clustering implemented using the AgglomerativeClustering class in scikit-learn 1.4.2. Dendrograms were created using the *dendrogram* function from the *hierarchy* module in SciPy 1.10.1. For each conformation, the total PIE, $\mathop \sum \limits_i \mathop \sum \limits_j PI{{E}_{i,j}}$, was calculated, where *i* and *j* denote residues from the ligand and receptor, respectively (Supplementary Fig. S1D). The mean and distribution of $\mathop \sum \limits_i \mathop \sum \limits_j PI{{E}_{i,j}}$ in each cluster were compared to identify the most stable cluster. Dot plots and box-and-whisker plots were depicted using Matplotlib 3.8.4 and Seaborn 0.13.2. Box boundaries indicate the 25th and 75th percentiles. Statistical significance was assessed using the Tukey–Kramer test with a family-wide error rate of 5%, implemented in statsmodels 0.14.2. In dot plots, different letters indicate groups that differ significantly. Representative conformations from stable clusters were visualized using PyMOL 2.6.0 [47].

In each coordinate belonging to the specified stable cluster, PIE values were summed across all residue *j* in the receptor to compute the total interaction energy for each ligand residue *i*, expressed as $\mathop \sum \limits_j PI{{E}_{i,j}}$ (Supplementary Fig. S1D). Within the cluster, the mean and standard deviations of $\mathop \sum \limits_j PI{{E}_{i,j}}$ were calculated to estimate the contribution of each ligand residue to the overall interaction. In addition to the total PIE, the interaction energies corresponding to electrostatic interaction (*E*es), dispersion interaction (*E*disp), and solvation energy (*G*sol) were also analyzed to compute $\mathop \sum \limits_j Ee{{s}_{i,j}}$, $\mathop \sum \limits_j \textit{Edis}{{p}_{i,j}}$, and $\mathop \sum \limits_j Gso{{l}_{i,j}}$, respectively.

### Conformational sampling of Rhino M1–S67 on the 9–1–1 complex

The conformation of the Rhino N-terminal sequence, M1–S67, on the core-ring structure of the 9–1–1 complex was analyzed as follows. The input sequence for AlphaFold2 included full-length Rhino, Rad9 (M1–S270), Hus1, and Rad1. In the subsequent SA-MD simulation, the input structure was composed of Rhino M1–S67, Rad9 M1–S270, Hus1, and Rad1. Each production run lasted 100 ns and was repeated 10 times, resulting in 400 averaged conformations. These conformations were sampled from the 300 K equilibration phases following the simulated annealing cycles (Supplementary Fig. S1A and B). For each conformation, PIE was calculated using the FMO2-DFTB3/PCM method (Supplementary Fig. S1C and D).

To model the interaction between Rhino and Rad9, the PIE values between Rhino P10–A31 and Rad9 M1–S270 were used to construct pairwise interaction matrices, which were analyzed using agglomerative hierarchical clustering. Based on the resulting dendrogram, the conformations were grouped into 12 clusters (Supplementary Fig. S2A and B). The most stable cluster was identified, and statistical significance was evaluated using the Tukey–Kramer test (Supplementary Fig. S2C).

For the Rhino–Rad1 interaction, PIE values were analyzed for three different Rhino segments: C30–T63 (Supplementary Fig. S4), T38–K46 (Supplementary Fig. S5), and T52–F61 (Supplementary Fig. S6). Each segment was paired with Rad1 to generate interaction matrices. These analyses resulted in 14, 11, and 24 clusters, respectively, from which the most stable cluster in each case was identified.

### Conformational sampling of Rhino S83–S104 on the Rad9 front pocket

The binding mode of the Rhino K90–F96 to the front pocket of Rad9 was modeled as follows. The input sequence for AlphaFold2 included Rhino F61–M140, Rad9 M1–S270, Hus1, and Rad1. In the subsequent SA-MD simulations, the Rhino S83–S104 segment was treated as the ligand, and Rad9 M1–S270, Hus1, and Rad1 were treated as the receptor. Each production run lasted 25 ns and was repeated 10 times, resulting in a total of 100 conformations. PIE values between Rhino S83–S104 and Rad9 M1–S266 were calculated to construct interaction matrices, based on which the conformations were grouped into 10 clusters (Supplementary Fig. S8A). A second round of SA-MD simulation was performed using the most stable conformations from clusters 2 and 6 in Supplementary Fig. S8B as starting structures. Each production run lasted 50 ns and was repeated 10 times, resulting in an additional 200 sampled conformations. The 100 conformations from the first round and 200 from the second round were combined and grouped into 22 clusters (Supplementary Fig. S8C and D).

### Conformational sampling of the Rad9 tail on the core-ring structure of the 9–1–1 complex

The conformation of the Rad9 tail on the core-ring structure of the 9–1–1 complex was modeled as follows. The input sequences for AlphaFold2 were composed of full-length Rad9, Hus1, and Rad1. The predicted structure was used as the initial model for further analysis. In the first round of conformational sampling, the same set of proteins was used as the input structures for SA-MD simulations. The high-temperature profile used either 1000 K, 800 K, or 650 K as the maximum temperature (Supplementary Fig. S9A). Production runs lasted 100 ns at 800 K and 650 K, and 50 ns at 1000 K. In total, 13 simulations were conducted (five runs at 1000 K and four runs each at 800 K and 650 K), and 420 conformations were extracted from the equilibration phases. PIE was calculated using the FMO2-DFTB3/PCM method. RMSFs of Cα atoms in the Rad9 C-terminal tail (H271–G391) were averaged across 13 production simulation runs to identify regions that stably interact with the core-ring structure, which were selected for further structural optimization (Fig. 7A).

PIE matrices were constructed between Rad9 H271–G391 (tail) and Rad9 M1–S270 (core ring) to evaluate the overall interaction. The sampled conformations were grouped into 10 clusters (Supplementary Fig. S9B), and stable clusters were selected based on total PIE values and the Tukey–Kramer test (Supplementary Fig. S9C and D).

### Iterative conformational sampling of the Rad9 tail on the Rad9 front pocket

To refine the conformation of the Rad9 tail on the Rad9 front pocket, PIE matrices between Rad9 residues P356–A371 and M1–S270 were constructed from the SA-MD simulation shown in Fig. 7A, and the sampled conformations were grouped into 18 clusters (Supplementary Fig. S10A). Representative structures from clusters 0, 6, and 8 in Supplementary Fig. S10B were used as the initial models in subsequent SA-MD simulations. Rad9 residues P356–L370 were treated as the ligand, and Rad9 M1–S270, Rad1, and Hus1 were used as the receptor (Supplementary Figs S11 and  S12). A maximum temperature of 1000 K was used in the high-temperature group (Supplementary Fig. S11A). Each production run lasted 25 ns and was repeated 10–20 times, resulting in 100–200 conformations sampled from the equilibration phases. SA-MD simulation and conformational sampling were iteratively conducted up to three consecutive rounds. Following each round, stable clusters were identified based on the sum of PIE values and statistical significance assessed by the Tukey–Kramer test (Fig. 7E and Supplementary Figs S11 and  S12).

The binding energy (*ΔG*_bind_) between Rad9 residues P356–L370 and M1–S270 was calculated for conformations within the identified stable clusters (clusters 0–3, 0–9, 8–3, 8–6, 6–7–4, and 6–7–2–6; Supplementary Fig. S13A and B). *ΔG*_bind_ was defined as *G*_complex_ – (*G*_receptor_* + G*_ligand_) and was calculated using the FMO3-DFTB3/PCM method, as described below. The resulting binding energies were statistically compared across clusters using the Tukey–Kramer test (Supplementary Fig. S13C).

### Conformational sampling of the Rad9 tail on Hus1

For the optimization of the Rad9 tail conformation on Hus1, the PIE values obtained from the initial SA-MD simulation in Fig. 7A were reanalyzed. PIE matrices between Rad9 residues H271–G391 and those of Hus1 were constructed to categorize the sampled conformations into 24 distinct clusters (Supplementary Fig. S15A).

In the second round of sampling, Rad9 residues Q286–R320 were treated as the ligand, and Rad9 M1–S270, Hus1, and Rad1 as the receptor. SA-MD simulation and conformational sampling were repeated using the most stable conformations from clusters 3, 11, and 21 in Supplementary Fig. S15A. The high-temperature profile used 1000 K as the maximum temperature, as shown in Supplementary Fig. S11A. Production runs were conducted for 50 ns and repeated 15 times, resulting in a total of 300 sampled conformations. PIE values were calculated to construct interaction matrices between Rad9 residues Q286–R320 and those of Hus1, and the resulting conformations were grouped into 20 clusters (Supplementary Fig. S16A).

In the third sampling, cluster 14 in Supplementary Fig. S16B was selected as the initial model. Under the same conditions as the second sampling, the SA-MD simulation was conducted for 25 ns and repeated 18 times, resulting in an additional set of 180 conformations. Before PIE analyses, the PIE datasets from the second and third sampling were merged, yielding a total of 480 PIE datasets. These were analyzed using interaction matrices between Rad9 residues S291–T313 and those of Hus1, and the 480 conformations were grouped into 60 clusters (Supplementary Fig. S17A). The top seven clusters that exhibited the lowest medians for the sums of PIEs were selected, and statistical significance was tested using Student’s or Welch’s *t*-test, with comparisons made against the most stable cluster, cluster 35.

### Conformational sampling of p21 H152–P164 on the Rad9 front pocket

To model the interaction of p21 and the Rad9 front pocket, the sequences of p21 H152–P164, Rad9 M1–S270, Rad1, and Hus1 were used as input for AlphaFold2. One of the top-ranked models was selected as the initial structure for the subsequent SA-MD simulation. Production simulations were performed for 50 ns and repeated 10 times, yielding a total of 200 sampled conformations. These conformations were then grouped into 11 clusters (Supplementary Fig. S19A).

### Calculation of the final binding free energy of a ligand–receptor complex

The final binding energy (*ΔG*_bind_) between each ligand peptide and the core-ring structure of the 9–1–1 complex was calculated to provide semi-quantitative energetic comparisons, as described previously [19]. The most stable structure in each cluster was used as the initial model. The terminal residues of the newly generated termini, which resulted from truncation, were neutralized by either deprotonating amino groups or converting carboxyl groups into aldehyde groups. Geometry optimization was first performed using the steepest descent algorithm in GROMACS, followed by refinement with the Hessian update algorithm in GAMESS for over 1000 steps under the FMO2-DFTB3/PCM method. The dispersion-corrected Gibbs free energy of the ligand–receptor complex in solvent (*G*_complex_), as well as those of the receptor (*G*_receptor_) and the ligand (*G*_ligand_), were calculated using the three-body expansion (FMO3) of the FMO method with DFTB3 and PCM (FMO3-DFTB3/PCM). The FMO3 calculations were performed in the same manner as the FMO2-DFTB3/PCM method, except that FMO3 includes three-body interactions to improve accuracy. The final binding free energy was calculated as *ΔG*_bind_* = G*_complex_ – (*G*_receptor_* + G*_ligand_). The following subcomplexes were analyzed: Rhino P10–A31 and Rad9 M1–S270; Rhino S83–S98 and Rad9 M1–S270; Rad9 T355–A371 and M1–S270; p21 H152–P164 and Rad9 M1–S270; Rhino T38–I48 and Rad1 D12–S282; Rhino T52–F61 and Rad1 M1–S282; and Rad9 S291–T313 and Hus1 M1–S280. The resulting complex structures were deposited as Supplementary PDB files. Additionally, the complex of Rhino T88–S98 with Rad9 M1–D267 was extracted from PDB 8WU8 and included for comparison.

The area of the interacting surface between a pair of proteins was calculated based on the solvent-accessible surface area of the individual monomer and the corresponding dimer, as calculated using the SASA command in GROMACS [48].

### APBS

Electrostatic potentials on the surfaces of Rad9 and Rad1 were calculated using the Adaptive Poisson–Boltzmann Solver (APBS). PDB files were converted to PQR format using PDB2PQR v2.1.1 [49] with the Amber force field. Electrostatic potential maps were then generated using APBS v3.0.0 [50]. The resulting potential, calculated on the solvent-accessible surface, was visualized on the van der Waals surface of the receptor protein using PyMOL. The potential values were color-coded from −5.0 kT/e (red) to +5.0 kT/e (blue).

### Conventional MD simulations of the 9–1–1 complex and Rhino

The conformational stability of Rhino on the 9–1–1 complex was evaluated using conventional MD simulations. The 9–1–1 complex consisted of the core-ring structure, including Rad9 M1–S270, Hus1, and Rad1. The C-terminal tail of Rad9 was excluded from all models.

For simulations of the 9–1–1 complex alone, the experimental structure 3G65 [11] was used as the initial model. This structure includes Rad9 M1–S270, Rad1 D13–E275, and Hus1 M1–S280. Missing loops and residues were reconstructed using MODELLER 10.1 [51]. This experimental structure and its derived MD simulation were used as references to assess the quality and stability of the subsequent MD simulations.

**Figure 1.**
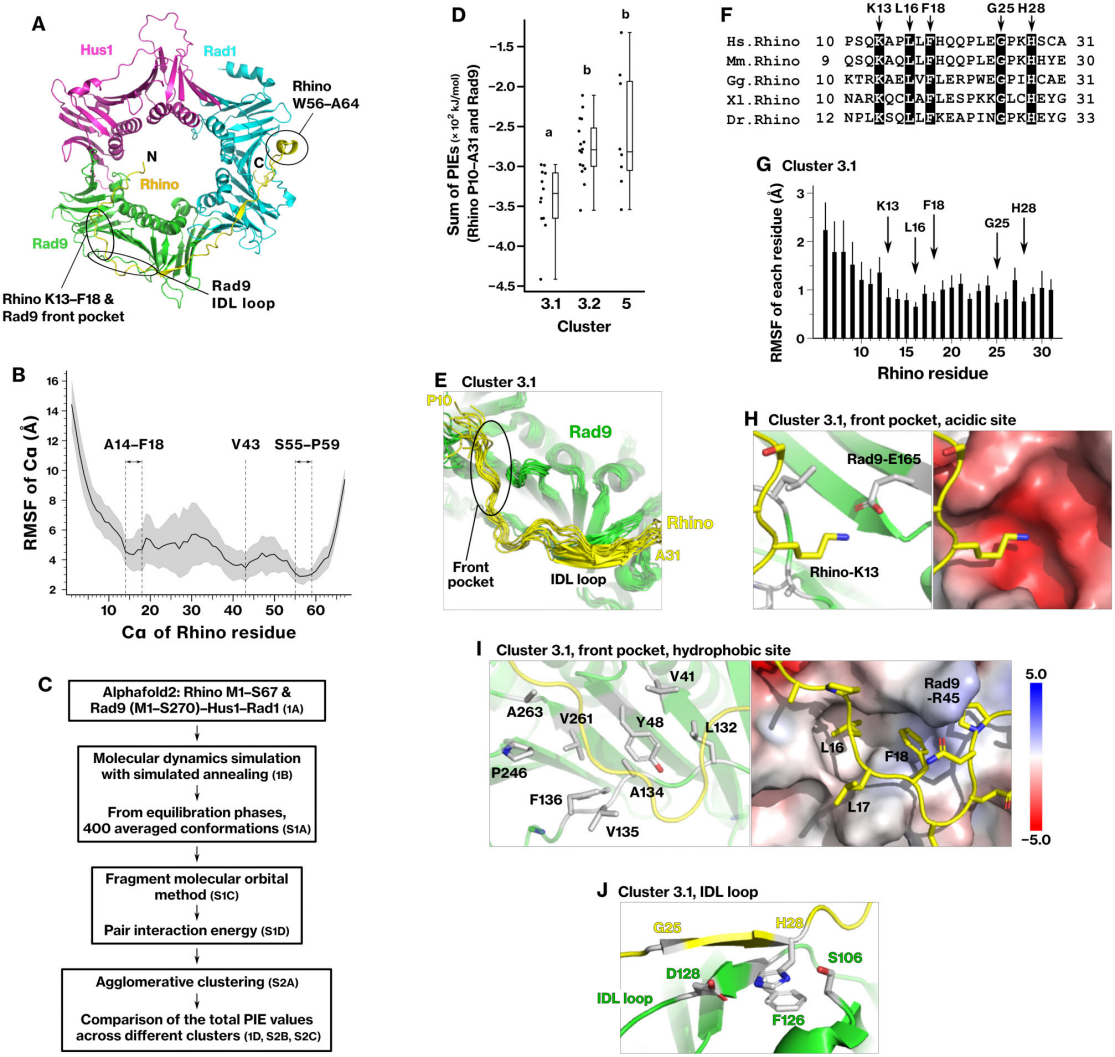
*In silico* analysis predicts the association of Rhino-K13, L16, and F18 with acidic and hydrophobic subpockets within the Rad9 front pocket. (**A**) The modeled structure of Rhino residues M1–S67, predicted by AlphaFold-Multimer, is shown on the front side of the 9–1–1 complex. IDL: interdomain linking. (**B**) Dynamics of the Rhino M1–S67 peptide were simulated using SA-MD for 100 ns. RMSF values were calculated for each alpha carbon (Cα) of Rhino residues. The graph represents the results from 10 independent simulations. The black line and gray area represent the mean and standard deviation, respectively. (**C**) The Rhino–9–1–1 complex structure was refined following the indicated scheme. Details are described in the “Materials and methods” section under Conformational sampling of Rhino M1–S67 on the 9–1–1 complex. (**D**) Dot plot showing the total PIE for each cluster identified in Supplementary Fig. S2. (**E**) Main chains of Rhino (P10–A31, yellow) and Rad9 (green) in all conformations within cluster 3.1. (**F**) Multiple sequence alignment of Rhino P10–A31 residues. (**G**) RMSFs were calculated for each Rhino M1–T31 residue across all conformations within cluster 3.1. Thick and thin bars represent the mean and standard deviation, respectively. (**H, I**) Acidic and hydrophobic subpockets of the Rad9 front pocket in the most stable conformation of cluster 3.1. Side chains of Rad9 (white) and Rhino (yellow) are depicted. (**J**) β-strand of Rhino and the IDL loop of Rad9. The corresponding PIEs and structure are shown in Table 1 and in Supplementary PDB file 1, respectively.

**Figure 2.**
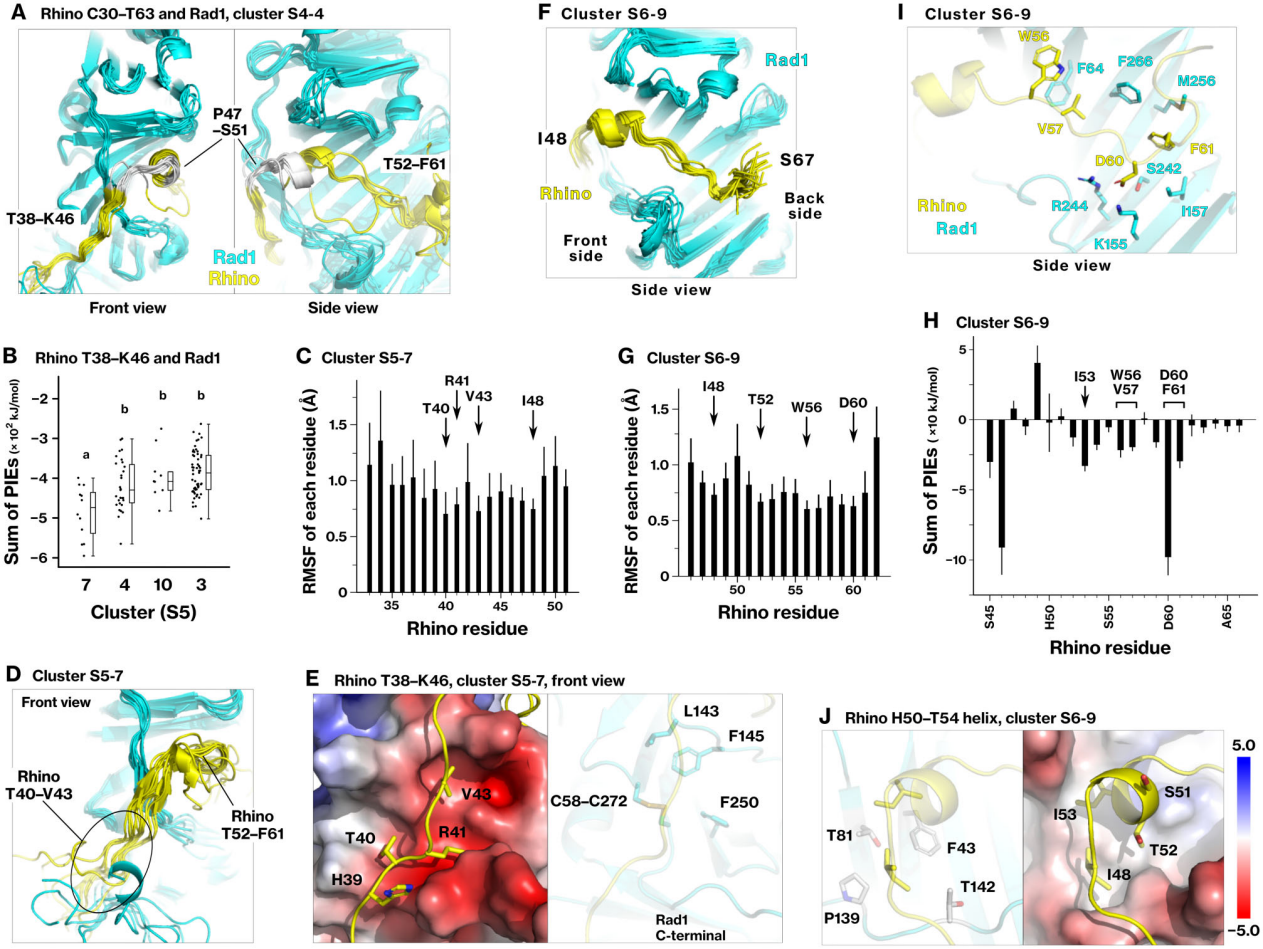
Rhino has two regions that interact with Rad1: residues T52–F61 and T38–K46. (**A**) Stable conformations of the Rhino C30–T63 segment (yellow) on Rad1 (cyan) are depicted for cluster 4 in Supplementary Fig. S4. Rhino P47–S51 are highlighted in white. (**B**) Dot plot showing the total PIE ($\mathop \sum \limits_i \mathop \sum \limits_j PI{{E}_{i,j}}$) for each conformation in stable clusters from Supplementary Fig. S5B. (**C**) RMSFs were calculated for Rhino residues T33–S51 and averaged within cluster 7 in Supplementary Fig. S5 (S5–7). Thick and thin bars represent the mean and standard deviation, respectively. The corresponding PIEs are shown in Table 2. (**D**) Main chains of Rhino (yellow) and Rad1 (cyan) from all conformations within cluster S5–7 are depicted from the front side of Rad1. (**E**) A hydrophobic pocket on the front side of Rad1 is depicted with side chains of Rhino (left) and Rad1 (right). (**F**) Main chains of Rhino (yellow) and Rad1 (cyan) in cluster 9 from Supplementary Fig. S6 (S6–9) are depicted from the outer side of Rad1. (**G**) RMSFs were calculated for Rhino residues T52–S61 in each conformation and averaged within cluster S6–9. (**H**) PIEs were summed for each Rhino residue across conformations in cluster S6-9. (**I**) The most stable conformation in cluster S6-9 is depicted. (**J**) The α-helix of Rhino H50–T54 is shown. The corresponding structures are provided in Supplementary PDB files 2 and 3.

**Figure 3.**
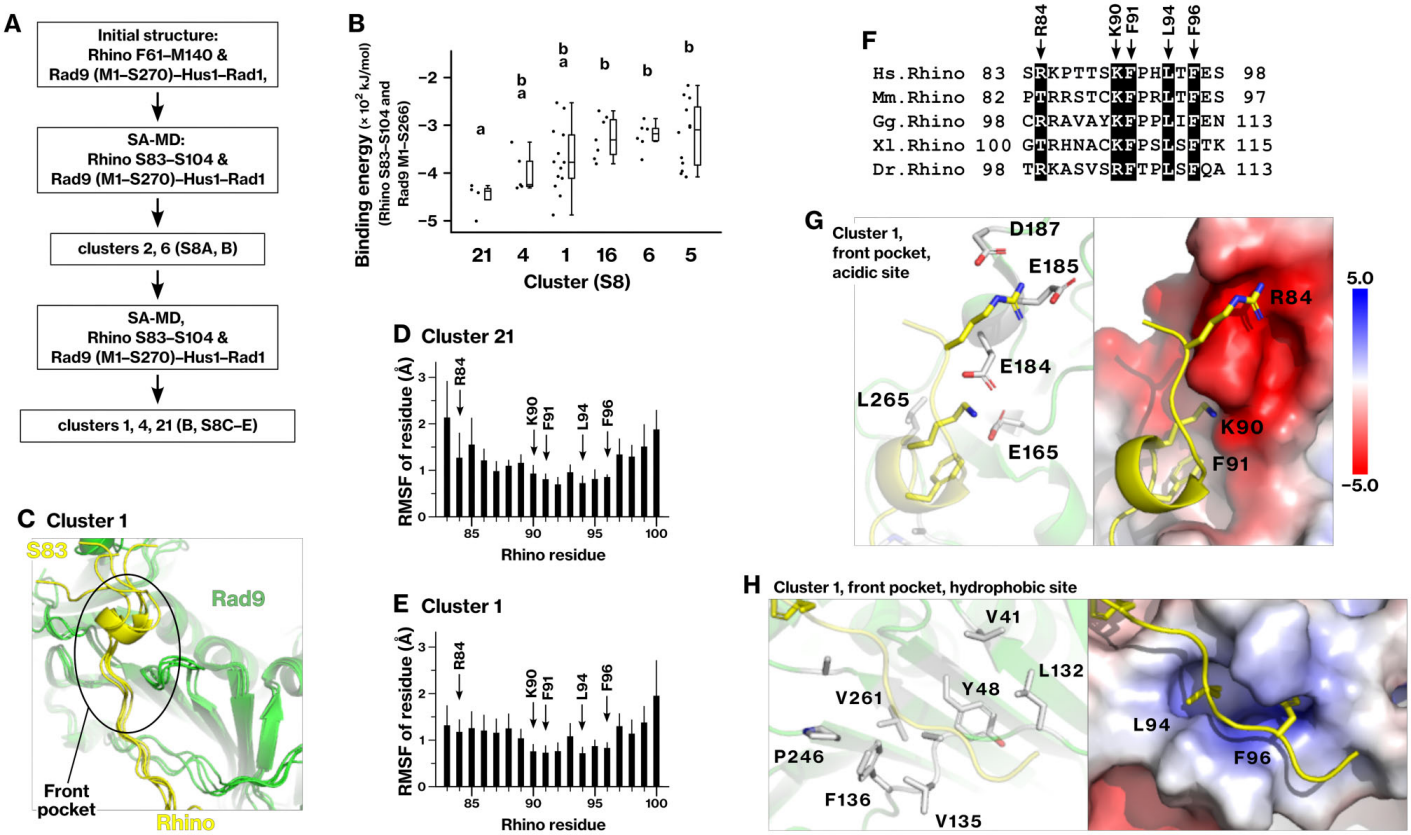
*In silico* modeling of Rhino K90–F96 binding to the Rad9 front pocket: structural mimicry of the Rad17 KYxxL motif. (**A**) The complex structure formed by Rhino S83–S104 and the core-ring structure of the 9–1–1 complex was analyzed as described in the scheme shown. Details are provided in the “Materials and methods” section under Conformational sampling of Rhino S83–S104 on the front pocket of Rad9. (**B**) Dot plot showing the total PIE for each cluster. (**C**) Main chains of Rhino (S83–S104, yellow) and Rad9 (green) for stable conformations within cluster 1. (**D, E**) RMSFs were calculated for Rhino S83–S104 residues and averaged across each cluster. Thick and thin bars represent the mean and standard deviation, respectively. (**F**) Multiple sequence alignment of Rhino S83–S98. (**G, H**) The Rhino peptide S83–S98 (yellow), bound to the front pocket of Rad9 (green), is shown for the most stable conformation in cluster 1. The corresponding PIEs, binding free energies, and structural coordinates are provided in Table 3, in Table 4, and in Supplementary PDB file 4, respectively.

For simulations involving Rhino, heterotetrameric models were constructed by combining Rad9 M1–S270, Rad1 M1–S282, Hus1 M1–S280, and either Rhino R8–S67 or Rhino S83–S98. For simulations of dimeric 9–1–1 complexes, two copies of Rad9 M1–S270, Rad1 M1–S282, and Hus1 M1–S280 were included, with Rhino placed on each monomer. Missing residues were modeled using MODELLER. Two dimeric models were generated: one containing Rhino M1–S104 and the other containing Rhino R41–S104. The most stable conformations in cluster 3.1 in Fig. 1D and in cluster 1 in Fig. 3B and Table 4 were used as modeling templates to maintain consistency and data continuity across Figs. 1–3, 5), and 6.

The complex structure was protonated using the H++ server. Topology files were generated using tLEaP following the same procedure as described earlier, except that a coarse-grained water model was incorporated. The 9–1–1 complex, either alone or bound to Rhino, was solvated using a hybrid solvation scheme combining the TIP3P and WatFour (WT4) water models [52]. First, a 10 Å shell of TIP3P water was added around the solute using the SOLVATESHELL command in tLEaP. This presolvated system was then embedded within a truncated octahedral box of WT4 water, maintaining a minimum distance of 10 Å between the TIP3P shell and the box wall, using the SOLVATEOCT command. The WT4 topology was incorporated from the SIRAH force field [53]. For simulations of the 9–1–1 dimer, the system was solvated in a truncated octahedral box filled with WT4 water, ensuring at least 20 Å between the solute and the box boundaries. All systems underwent energy minimization and equilibration under both NVT and NPT ensembles at 310 K. Conventional MD simulations were conducted at a constant temperature of 310 K with a 2-fs time step. Production runs were performed for 1000 ns.

### Multiple sequence alignment and profile hidden Markov models

A profile hidden Markov model (HMM) for the conserved KYxxL+ motif was constructed using the hmmbuild module of HMMER v.3.3.2 [54]. The multiple sequence alignment (Fig. 10A) was used as input. During HMM construction, all sequences were treated as effective sequences with equal weights based on the specific relative entropy at each alignment position. The resulting profile HMM was converted to an HMM logo using the hmmlogo module of HMMER and visualized using Skylign [55]. A KYxxL motif in *Schizosaccharomyces pombe* Rad17 was identified using AlphaFold2.

### Co-precipitation assay of Rhino and the 9–1–1 complex

The interaction between Rhino and the 9–1–1 complex was examined as described previously [18, 19]. In brief, COS-1 cells were maintained in DMEM supplemented with 5% bovine serum and 1% fetal bovine serum. Cells were seeded at 4.0–5.0 × 10^5^ cells per 35-mm dish and incubated overnight. The cells were transfected with 1.0 μg of plasmid DNA and 6.5 μg of acidified polyethylenimine [56]. Prior to transfection, the medium was replaced with 1.0 ml of fresh DMEM/5% BS/1% FBS. This reduced volume was empirically found to improve transfection efficiency significantly. After 24 h, the medium was replaced with 2.0 ml of fresh medium. At 48 h post-transfection, cells were harvested, washed with ice-cold PBS, and lysed with at least 20 packed cell volumes of low-salt buffer [10 mM HEPES-NaOH (pH 7.8), 10 mM KCl, 0.1% Triton X-100, 10% glycerol, 0.34 M sucrose, and 1.5 mM MgCl_2_], supplemented with a protease and phosphatase inhibitor cocktail [57]. Lysis was carried out on ice for 20 min. The lysate was clarified by centrifugation (17 900 × *g*, 20 min) and collected as the low-salt extract. Samples were prepared in duplicate for each independent experiment. Untransfected cells were used as controls.

Rhino-flag was immunoprecipitated, and co-precipitation of the endogenous 9–1–1 complex was detected by western blotting using an anti-Rad1 antibody. The low-salt extract was incubated overnight at 4°C with M2 agarose beads in binding buffer [20 mM HEPES-NaOH (pH 7.8), 120 mM NaCl, 0.2% Triton X-100]. Beads were then collected by centrifugation and washed seven times with wash buffer (identical to the binding buffer but containing 0.1% Triton X-100). Bound proteins were eluted by boiling in Laemmli sodium dodecyl sulfate–polyacrylamide gel electrophoresis (SDS–PAGE) sample buffer. Eluted samples were analyzed using SDS–PAGE followed by western blotting. Signal intensities from anti-Rad1 blots were quantified using ChemiDoc XRS+ (Bio-Rad) and normalized to corresponding anti-FLAG signals. Statistical significance was evaluated using either Student’s or Welch’s t-test. The antibodies used were as follows: anti-FLAG, Medical & Biological Laboratories, PM020; M2 agarose, Merck Millipore Sigma, A2220; anti-Rad1, Santa Cruz Biotechnology, N-18, sc-14314. Wild-type Rhino or the K13–F18 mutant was used as a reference for quantification, and their signal intensities were set to 100%.

The insoluble fraction of the low-salt extract contains chromatin [57, 58], and the chromatin fraction was solubilized for immunoprecipitation assay, as previously described [57, 59]. The insoluble pellet obtained from the final centrifugation step during the low-salt extract preparation was washed once with low-salt buffer and then resuspended in the same buffer. The suspension was sonicated using an ultrasonic disruptor (TOMY DIGITAL BIOLOGY, Japan, Cat. No. UR-21P) for 10 s while cooling on ice. This procedure was repeated 10 times, with at least a 1-min interval between successive sonication. The resulting suspension was clarified by centrifugation (17 900 × *g*, 20 min), and the supernatant was collected as the low-salt buffer-solubilized chromatin fraction for subsequent immunoprecipitation assay. To induce replication stress, thymidine was added to the cell culture medium at a final concentration of 4 mM, 24 h post-transfection. Cells were then incubated with thymidine for an additional 24 h prior to harvest.

### Plasmids

The coding sequence of human Rhino (NM_001252499.3) was synthesized and cloned into the pTwist CMV BG WPRE Neo vector by Twist Bioscience (California, USA). Two FLAG tags were fused to the C-terminus of Rhino. Mutant constructs encoding Rhino variants, including K13E/P15S/L16S/L17S/F18S, F91A/L94A/F96A, and R84A/K85A/K90E/F91S/L94S/F96S, were also synthesized.

## Results

### *In silico* analysis suggests Rhino interacts with the 9–1–1 complex through three conserved regions

AlphaFold-Multimer predicted the complex structure of Rhino and the 9–1–1 complex and the association of Rhino M1–S67 with the front side of the 9–1–1 complex, the same side that interacts with the Rad17–RFC2–5 complex (Fig. 1A). Rhino was predicted to bind Rad9 through a PIP box-like motif (K13–F18) and subsequent loop region, similar to the C-termini of the PCNA-binding proteins p21 and FEN1 [60, 61]. K13–F18 contacts a conserved front pocket of Rad9, which binds to the Rad17 KYxxL motif in humans [5] and *S. cerevisiae* [6, 7]. Rhino W56–A64 was predicted to bind to a hydrophobic groove on Rad1, consistent with a previous co-crystal structure using a Rhino-derived peptide [26]. These structural features suggest that Rhino makes extensive contacts with the front side of the 9–1–1 complex.

To evaluate the stability of these interactions, SA-MD simulations were performed using the AlphaFold2-derived model. The simulations revealed three regions within Rhino, A14–F18, V43, and S55–P59, which exhibited reduced RMSF values (Fig. 1B). These results suggest that the M1–S67 region anchors Rhino to the 9–1–1 complex via three conserved and conformationally stable interaction sites.

### Rhino residues K13 and L16/F18 associate with chemically distinct subsites in the Rad9 front pocket

We analyzed the trajectory of the SA-MD simulation shown in Fig. 1B to identify the most stable binding conformation of Rhino residues P10–A31 with Rad9 (Fig. 1C). As described in the “Materials and methods” section under Conformational sampling of Rhino M1–S67 on the 9–1–1 complex, analysis of PIE matrices identified cluster 3 as the most stable (Supplementary Fig. S2A and B), exhibiting the largest sum of PIEs, $\mathop \sum \limits_i \mathop \sum \limits_j PI{{E}_{i,j}}$, where *i* and *j* represent residues from Rhino P10–A31 and Rad9, respectively (Fig. 1D and Supplementary Fig. S2A–C). Cluster 3 was further divided into subclusters 3.1 and 3.2, and subcluster 3.1 was determined to be the most stable (Fig. 1D).

In cluster 3.1, Rhino P10–A31 was associated with the front pocket and interdomain-linking loop (IDL loop) of Rad9 (Fig. 1E). Rhino-K13 showed the highest binding energy in the analysis of the sum of PIEs for each Rhino residue *i*, $\mathop \sum \limits_j PI{{E}_{i,j}}$ (Table 1; Supplementary Fig. S1D) and is conserved in vertebrates (Fig. 1F). Rhino residues L16 and F18 were conserved and showed smaller RMSF than that of the surrounding residues (Fig. 1G), indicating that the conformation of these residues was stable and functioned as anchoring points during the SA-MD simulation.

**Table 1.** Pair interaction energies (PIEs) between Rhino P10‒A31 and Rad9 M1‒S270, corresponding to the interactions shown in Fig. 1

Rhino residue	PIE	*E*es	*E*disp	*G*sol
**K13**	**−126 ± 15**	−220 ± 13	−12 ± 1	107 ± 12
**H28**	**−43 ± 16**	−24 ± 12	−13 ± 2	−8 ± 4
**L16**	**−27 ± 1**	−6 ± 2	**−13 ± 1**	**−10 ± 2**
**G25**	**−20 ± 6**	−13 ± 5	−3 ± 1	−5 ± 2
E24	−19 ± 16	51 ± 22	−7 ± 2	−63 ± 8
Q20	−18 ± 8	−5 ± 5	−6 ± 2	−7 ± 6
H19	−18 ± 6	−11 ± 4	−6 ± 2	−2 ± 3
**F18**	**−15 ± 3**	−3 ± 3	**−13 ± 2**	0 ± 2

The PIEs for each Rhino residue (*i*), $\mathop \sum \limits_j PI{{E}_{i,j}}$, were summed over all conformations, and the corresponding means and standard deviations were calculated for cluster 3.1, as defined in Fig. 1. Electrostatic interactions (*E*es_*i,j*_), dispersion interactions (*E*disp_*i,j*_), and solvation energies (*G*sol_*i,j*_) were computed using pair interaction energy decomposition analysis. Only residues with substantial contributions to the interaction energy are listed. All values are in kJ/mol.

Further analysis revealed that K13, L16, and F18 were engaged in two chemically distinct regions of the Rad9 front pocket. The front pocket of Rad9 was composed of two distinct regions: an acidic and a hydrophobic subpocket (Supplementary Fig. S3A and B). Rhino-K13 interacted with the acidic subpocket (Fig. 1H), consistent with the large electrostatic interaction energy (*E*es; Table 1) indicated by pair interaction energy decomposition analysis (PIEDA). In contrast, Rhino-L16 and F18 interacted with a hydrophobic subpocket (Fig. 1I). Most of the interaction energy resulted from dispersion interaction (*E*disp) and solvation energy (*G*sol; Table 1), suggesting the contribution of hydrophobic and van der Waals interactions. These results indicate that Rhino-K13, L16, and F18 act as anchoring residues that secure Rhino to the Rad9 front pocket. Their binding pattern is structurally conserved and was later defined as the KYxxL+ motif.

### Rhino G25–H28 residues form a β-strand that stabilizes the interaction with the Rad9 IDL loop

Rhino-G25 and H28 were also conserved and constituted an additional interaction site contributing to the binding energy (Fig. 1F; Table 1). These residues exhibited smaller RMSF values than surrounding residues, indicating their conformational stability (Fig. 1G). In this region, main-chain hydrogen bonds connected two antiparallel β-strands: one formed by Rhino G25–C30 and the other by Rad9 F126–D128 within the IDL loop (Fig. 1J and Supplementary Fig. S3C). These results indicate that the β-strand formed by Rhino G25–H28 stabilizes the interaction with the Rad9 IDL loop, contributing to the overall binding affinity.

The most stable conformation in cluster 3.1 was geometry optimized and used to calculate the final binding energy (*ΔG*_bind_). The final binding free energy between Rhino P10–A31 and Rad9 core-ring structure was estimated as −610 kJ/mol using the FMO3-DFTB3/PCM method (Table 12).

### Rhino contains two Rad1-interacting regions, T38–K46 and T52–F61

The interaction between Rhino C30–T63 and Rad1 was analyzed using the same trajectories employed for the analysis of the Rhino–Rad9 interaction as described in the “Materials and methods” section, and clusters 3 and 4 were identified as stable conformational states (Supplementary Fig. S4A–C). The sum of the PIEs for each Rhino residue revealed that Rhino has two regions associated with Rad1: T38–K46 and T52–F61 residues (Supplementary Fig. S4D). These regions were separated by residues P47–S51 (Fig. 2A), and two discrete Rad1-binding regions were identified within the N-terminal segment of Rhino.

### Rhino-V43 occupies the hydrophobic pocket on the front side of Rad1

The results of the SA-MD simulation were reanalyzed, focusing on the Rhino T38–K46 residues, and cluster 7 was identified as the most stable cluster (Fig. 2B and Supplementary Fig. S5A–C). In cluster 7, the Rhino-T40, R41, V43, and I48 showed lower RMSF (Fig. 2C). Rhino-R41 and V43 were associated with the acidic and hydrophobic subpockets on the front side of Rad1 (Fig. 2D and E) via electrostatic and hydrophobic interactions (Table 2), respectively. These interactions are described in detail in Supplementary Results 1. Based on the most stable structure in cluster 7, the final binding energy of the Rhino T38–I48 peptide to Rad1 was estimated to be about −482 kJ/mol using the FMO3-DFTB3/PCM method (Table 12). These findings define T38–I48 as a discrete, structurally stable region through which Rhino interacts with Rad1.

**Table 2.** PIEs between Rhino T38‒K46 and Rad1 M1–S282, corresponding to the interactions shown in Fig. 2

Rhino residue	PIE	*E*es	*E*disp	*G*sol
**R41**	**−167 ± 25**	−287 ± 29	−15 ± 4	133 ± 22
K46	−150 ± 30	−287 ± 23	−8 ± 1	145 ± 14
**T40**	−42 ± 24	−30 ± 17	−9 ± 4	−5 ± 13
**V43**	**−30 ± 5**	−3 ± 7	**−11 ± 2**	**−16 ± 4**
H39	−30 ± 24	−24 ± 16	−6 ± 2	1 ± 12

The *PIE*_*i,j*_, *E*es_*i,j*_, *E*disp_*i,j*_, and *G*sol_*i,j*_ for each Rhino residue (*i*) were summed across conformations, and the means and standard deviations were calculated for cluster S5–7, as defined in Fig. 2. All values are in kJ/mol.

### Rhino T52–F61 contains three subregions that interact with the outer side of Rad1

The conformation of Rhino T52–F61 on Rad1 was analyzed in a similar manner to Rhino T38–K46, and cluster 9 was identified as the most stable (Supplementary Fig. S6A and B). Rhino T52–F61 was associated with the outer side of Rad1 (Fig. 2F and I). Across this region, the RMSF values were uniformly low, and three peaks were observed in the interaction energy profile (Fig. 2G and H; details are provided in Supplementary Results 1). Our model of Rhino S55–A64 on Rad1 in cluster 9 closely matched the experimental structure of PDB 6J8Y [26], validating our integrative optimization pipeline that combines SA-MD simulations, FMO calculations, and conformational clustering.

In addition to the previously reported S55–A64 residues, the upstream H50–T54 residues formed an α-helical structure, which created an additional contact site (Fig. 2J). Notably, Rhino-S51, which is phosphorylated and involved in its interaction with DNA polymerase theta [62], was located on the solvent-exposed face of the helix in our model, suggesting that S51 remains accessible even when Rhino is associated with the 9–1–1 complex.

The calculated binding energy, derived from the optimized structure in cluster 9, was −381 kJ/mol (Table 12). Collectively, these findings indicate that the I48–F61 segment of Rhino contributes to a stable conformation on the Rad1 surface.

### Structural characterization of Rhino K90–F96 binding to the Rad9 front pocket

The structure of the Rhino T88–S98 segment bound to the Rad9 front pocket has been experimentally reported; however, we hypothesized that the reported structure may require refinement. The structure was analyzed by X-ray crystallography using a synthetic peptide corresponding to the T88–S98 region of Rhino [63]. This region contains a conserved KFxxLxF sequence. However, in the crystal structure (PDB 8WU8), the acidic subpocket of the Rad9 front pocket was occupied by the N-terminal amino group of residue T88 instead of Rhino-K90 (Supplementary Fig. S7A and B). Therefore, we hypothesized that this interaction is likely an artifact caused by the charged N-terminal amino group of the synthetic peptide. In line with this hypothesis, binding energy calculations using the crystal structure (PDB 8WU8) yielded a relatively weak binding energy of −117 kJ/mol (Table 4). These observations prompted us to revisit the structure of Rhino K90–F96 on Rad9 using *in silico* modeling.

To refine the interaction model of Rhino K90–F96 with Rad9, we examined its binding conformation using an *in silico* approach. AlphaFold2 predicted the association of Rhino K90–F96 with the front pocket of Rad9 in an analysis of Rhino F61–M140 residues. We further optimized the binding conformations of Rhino S83–S104, as described in the “Materials and methods” section, under Conformational sampling of Rhino S83–S104 on the Rad9 front pocket (Fig. 3A and Supplementary Fig. S8). Cluster 21 was identified as the most stable, exhibiting statistically significant differences from all other clusters, except for clusters 1 and 4 (Fig. 3B and Supplementary Fig. S8D and E). Cluster 4 was excluded from subsequent analyses because it exhibited substantially weaker binding energy (Table 4).

We analyzed the interaction energies and flexibility profiles of Rhino S83–S104 in clusters 1 and 21. PIE calculations revealed that the R84 and K90 residues showed the largest interaction energies (Table 3). F91, L94, and F96 exhibited interaction energies arising from dispersion and solvation, and showed the lowest RMSF (Fig. 3D and E), forming a conformationally stable core within the segment. These key residues were conserved across vertebrate Rhino proteins (Fig. 3F), underscoring their functional relevance.

**Table 3.** PIEs between Rhino S83‒S104 and Rad9 M1–S270, corresponding to the interactions shown in Fig. 3

Rhino residue	PIE	*E*es	*E*disp	*G*sol
Cluster 21				
R84	**−217 ± 18**	−294 ± 14	−9 ± 3	86 ± 14
K90	**−207 ± 53**	−314 ± 40	−9 ± 2	115 ± 20
F91	**−15 ± 3**	3 ± 3	**−14 ± 2**	**−6 ± 0**
L94	**−21 ± 3**	−3 ± 4	**−13 ± 1**	**−6 ± 2**
F96	**−17 ± 7**	0 ± 3	**−13 ± 6**	**−4 ± 3**
Cluster 1				
R84	**−147 ± 44**	−239 ± 42	−9 ± 3	100 ± 22
K90	**−195 ± 36**	−275 ± 35	−7 ± 1	87 ± 17
F91	**−10 ± 5**	12 ± 4	**−14 ± 2**	**−9 ± 4**
L94	**−20 ± 4**	−4 ± 2	**−12 ± 2**	**−6 ± 2**
F96	**−16 ± 4**	−1 ± 4	**−12 ± 2**	**−3 ± 1**

The *PIE*_*i,j*_, *E*es_*i,j*_, *E*disp_*i,j*_, and *G*sol_*i,j*_ for each Rhino residue were summed across conformations, and the means and standard deviations were calculated for clusters 1 and 21, as defined in Fig. 3. All values are in kJ/mol.

We examined the structural features of cluster 1 in more detail. Rhino-R84, K90, and F91 were positioned within the acidic subpocket of the Rad9 front pocket (Fig. 3G), whereas L94 and F96 occupied the hydrophobic subpocket (Fig. 3H). The overall topology was consistent with that of the Rad17 KYxxL motif (Fig. 7N and O) and Rhino K13–F18 region (Fig. 1). A key structural difference between the modeled and experimental conformations (PDB 8WU8) is the positioning of K90. In clusters 1 and 21, K90 was properly inserted into the acidic subpocket of Rad9 and contributed significantly to binding energy and stability (Fig. 3D, E, and G; Table 3). These results suggest that Rhino K90–F96 engages the Rad9 front pocket through the same fundamental mechanism as other Rad9-binding segments, a binding pattern later referred to as the KYxxL+ motif.

The final binding energies, *ΔG*_bind_, for the interaction between Rad9 and Rhino were calculated, yielding −515 and −411 kJ/mol for clusters 1 and 21, respectively (Table 4). These findings demonstrate that energetically favorable conformations were successfully identified through the SA-MD simulation.

**Table 4.** Binding energy of the Rhino S83‒S104 segment bound to the Rad9 core-ring structure (M1–S270), corresponding to the interactions shown in Fig. 3

Cluster	*ΔG*_bind_ (kJ/mol)
PDB 8WU8	−117
1	−515
4	−175
21	−441

The binding free energy (*ΔG*_bind_) was calculated as *ΔG*_bind_ = *G*_complex_ − (*G*_Rad9_ + *G*_Rhino_) using the FMO3-DFTB3/PCM method as described in the “Materials and methods” section under Calculation of the final binding free energy of a ligand–receptor complex. For comparison, the structure from PDB 8WU8 was optimized, and its binding energy was evaluated in the same manner.

### Rhino K13–F18 residues are important for interaction with the 9–1–1 complex *ex vivo*

**Figure 4.**
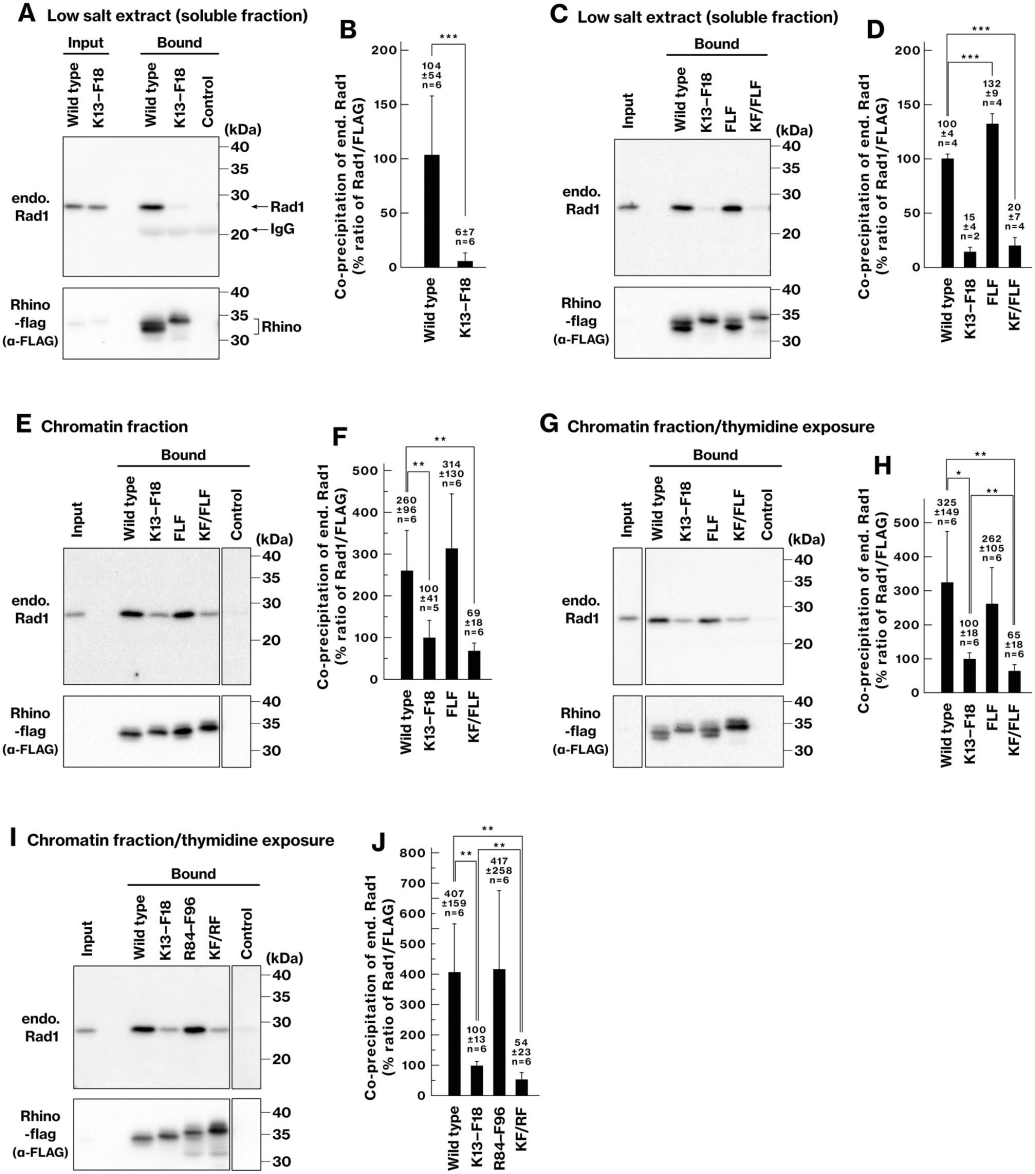
Rhino K13–F18, but not F91/L94/F96, residues are important for interaction with the 9–1–1 complex *ex vivo*. (**A**–**D**) COS-1 cells were transfected with vectors expressing Rhino-flag, and low-salt extracts, depleted of chromatin, were prepared. Rhino-flag was precipitated, and the co-precipitation of endogenous Rad1 was examined. The input represents 1.5% of the total lysate. Graphs show the results from three (B) and two (D) independent experiments. (**E**–**J**) The same experiment was performed as in panels (A–D), except that low-salt buffer-insoluble chromatin fractions were solubilized and used for the co-precipitation assay. In panels (G–J), cells were exposed to thymidine for 24 h prior to harvesting. All lanes shown were obtained from the same blot. Graphs represent the results of three independent experiments. K13–F18 (KF), FLF, and R84–F96 (RF) indicate the K13E/P15S/L16S/L17S/F18S, F91A/L94A/F96A, and R84A/K85A/K90E/F91S/L94S/F96S mutants of Rhino, respectively. Data are presented as mean ± standard deviation. *n* indicates the number of biological replicates. * *P* < 0.05; ** *P* < 0.01; *** *P* < 0.001.

Our *in silico* analyses predicted that Rhino contains two conserved motifs that bind to the front pocket of Rad9: K13–F18 and K90–F96. We examined which of these served as the primary interaction sites *ex vivo*. We evaluated the interaction using the soluble fraction of cell extracts, which excludes chromatin-bound proteins [57]. Endogenous Rad1 co-precipitated with wild-type Rhino-flag (Fig. 4A), confirming the formation of the Rhino–9–1–1 complex. The K13–F18 mutation (K13E/P15S/L16S/L17S/F18S) abolished this interaction (Fig. 4A and B), verifying the *in silico* results. By contrast, endogenous Rad1 co-precipitated with the F91A/L94A/F96A mutant, despite substitution of the three residues identified as Rad9-binding residues in both our *in silico* model (Fig. 3) and the crystal structure [63] (Fig. 4C and D). These results indicate that the T88–S98 region is dispensable for Rad9 binding in the soluble fraction and that K13–F18 is the primary functional interaction site.

### Rhino K90–F96 residues contribute to Rhino–9–1–1 complex formation in the chromatin fraction

We further characterized the interaction between Rhino and the 9–1–1 complex in the chromatin context using a solubilized chromatin fraction prepared for immunoprecipitation assays [57, 59]. These assays were performed in the presence or absence of thymidine exposure, which induces replication stress and activates the ATR-dependent checkpoint [59]. In this fraction, endogenous Rad1 co-precipitated with exogenously expressed wild-type Rhino-flag regardless of thymidine exposure, and the K13–F18 mutation substantially reduced this interaction (Fig. 4E, G, I). Notably, a substantial amount of Rad1 (25%–38% of the wild-type level) was still co-precipitated with the Rhino K13–F18 mutant (Fig. 4F, H, J). Additional mutations, F91A/L94A/F96A or R84A/K85A/K90E/F91S/L94S/F96S, decreased the residual interaction to 65% and 54% of that observed for the K13–F18 mutant, respectively (Fig. 4I and J). These results support the previous crystallographic data [63] and suggest that the R84–F96 region contributes to the binding of Rhino to the 9–1–1 complex in the chromatin fraction.

### Stability of the interaction between Rhino and the 9–1–1 complex in MD simulation

**Figure 5.**
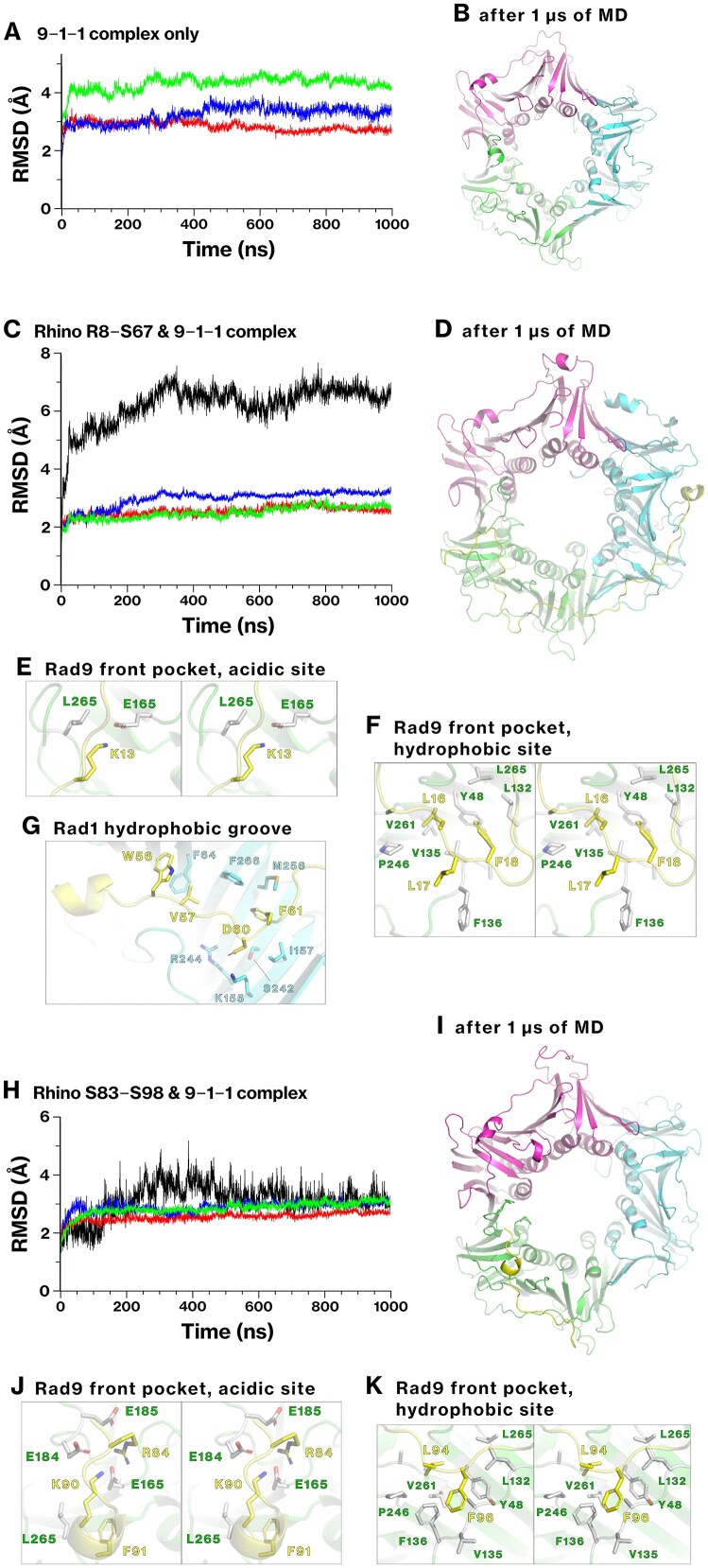
Stability of the association of Rhino with the 9–1–1 complex in MD simulations. The dynamics of the 9–1–1 complex and Rhino were simulated at 310 K for 1 μs. Three simulation setups were evaluated: the 9–1–1 complex alone (**A, B**); the complex with the Rhino M1–S67 peptide (**C**–**G**); and the complex with the Rhino S83–S104 peptide (**H**–**K**). RMSD was calculated for Rad9 (green), Rad1 (blue), Hus1 (red), and Rhino (black). For Rhino, RMSD was calculated for the R8–S67 (C) and S83–F96 (H) segments. After 1 μs of simulation, the final conformations were visualized for the interfaces between Rhino and Rad9 or Rad1. The corresponding structures are provided in Supplementary PDB files 10 and 11.

We evaluated the dynamic stability of the interaction between Rhino and the 9–1–1 complex using conventional MD simulations to validate the predicted binding modes. The core-ring structure of the 9–1–1 complex was used for all analyses, excluding the Rad9 C-terminal tail. First, the dynamics of the 9–1–1 complex without any ligand peptides were evaluated. The root-mean-square deviation (RMSD) of Rad9 (M1–S270), Hus1, and Rad1 reached a plateau at 3–4 Å, reflecting the basal conformational stability of each subunit (Fig. 5A), and the overall structure remained stable (Fig. 5B). This simulation served as a reference for the subsequent simulations, as it was based on the experimental structure.

Next, we performed MD simulations using the 9–1–1 complex associated with either the Rhino R8–S67 or S83–S98 peptide. In simulations with the Rhino R8–S67 peptide, Rhino exhibited a higher RMSD than the 9–1–1 subunits (Fig. 5C), largely due to the internal flexibility of residues L23–T38; however, the interfaces between Rhino and either Rad9 or Rad1 were preserved throughout the 1 μs simulation (Fig. 5D–G). Rhino residues K13, L16, and F18, key residues of the KYxxL+ motif, retained stable contact with the Rad9 front pocket (Fig. 5E and F). Similarly, in the 1 μs simulation involving the Rhino S83–S98 peptide, residues K90, F91, L94, and F96, also key residues of the KYxxL+ motif, maintained stable interactions with the Rad9 front pocket (Fig. 5I–K). These results indicate that the KYxxL+ motifs in the Rhino R8–S67 and S83–S98 residues possess sufficient structural stability to sustain their interactions with Rad9.

### MD simulation of the dimeric structures of the 9–1–1 complexes

**Figure 6.**
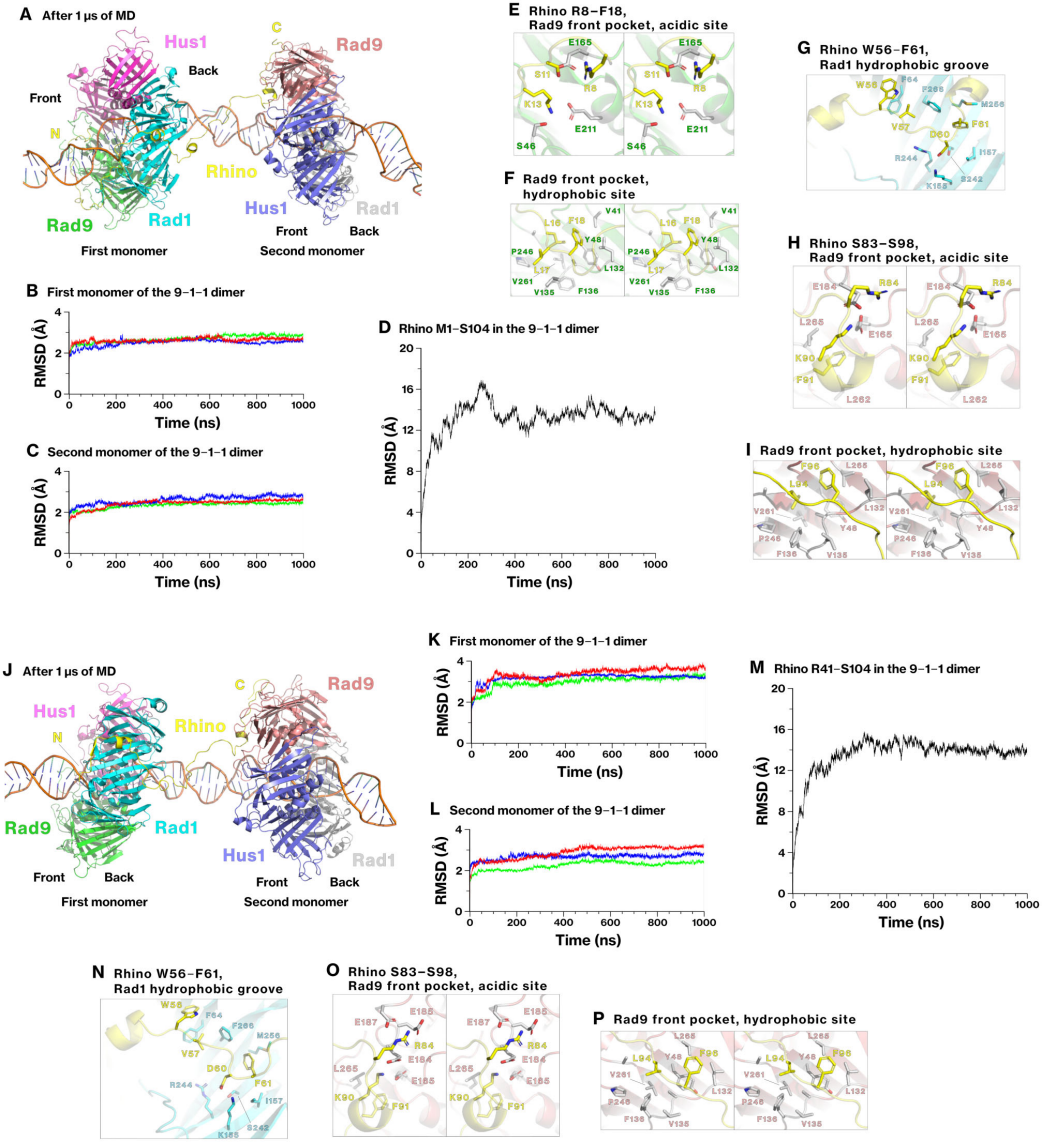
Stability of dimers composed of the 9–1–1 complexes connected by Rhino in molecular dynamics simulations. (**A**–**I**) Two 9–1–1 complexes were connected via the Rhino peptide M1–S104, and the resulting dimer was placed on double-stranded DNA. The dynamics were simulated using conventional MD at 310 K for 1 μs. The front and back sides of each monomer, as well as the N- and C-termini of the Rhino peptide, are indicated (A). RMSD was calculated for Rad9, Hus1, Rad1, and Rhino (B–D). Enlarged panels show the interface structures between Rhino and Rad9 or Rad1 at the end of the MD simulation (E–I). (**J**–**P**) The same MD simulation was performed, except that the Rhino peptide R41–S104 was used to exclude the first KYxxL+ motif located in K13–F18. The corresponding structures are provided in Supplementary PDB files 12 and 13.

Rhino contains two distinct regions interacting with the Rad9 front pocket (Figs 1, 3, and 4). We hypothesized that this feature would enable Rhino to bridge the two Rad9 subunits and connect two 9–1–1 complexes. To explore this possibility, we constructed a dimeric model of the 9–1–1 complex by incorporating the Rhino M1–S104 peptide. In this model, the R8–S67 region of Rhino interacts with Rad9 and Rad1 in the first monomer, whereas the S83–S98 region binds to Rad9 in the second monomer. The second 9–1–1 complex was positioned at the backside of the first complex (Fig. 6A), according to the spatial orientation of Rhino, whose C-terminal region extends toward the back of the complex (Figs 1 and 2). Double-stranded DNA was placed along the central axis of the dimer, assuming that such a dimer could form on chromatin. This assumption is supported by our observation that mutations in the S83–S93 segment of Rhino, which includes the second KYxxL+ motif, significantly reduced its co-precipitation with the 9–1–1 complex, specifically in the chromatin fraction (Fig. 4G–J).

The structural stability of the dimeric 9–1–1 complex bridged by Rhino was assessed using conventional MD simulations, and the two monomers remained connected through Rhino throughout a 1 μs simulation (Fig. 6A). All subunits of the 9–1–1 complex exhibited RMSD values below 3 Å (Fig. 6B and C), and the RMSD of Rhino plateaued (Fig. 6D), indicating that the entire assembly remained structurally stable. The interfaces between Rhino and the 9–1–1 complexes remained stable throughout the simulation, including those mediated by the K13–F18, T52–F61, and S83–S98 segments of Rhino (Fig. 6E–I). These results demonstrate that the dimeric configuration of the 9–1–1 complexes remains conformationally stable under simulated physiological conditions.

### Bridging of 9–1–1 complexes by Rhino in the absence of the K13–F18 segment

In the solubilized chromatin fraction, Rhino retained a substantial interaction with the 9–1–1 complex even when the K13–F18 segment was mutated, indicating that this region is important, but not essential, for binding to the 9–1–1 complex under chromatin-bound conditions (Fig. 4E–J). Furthermore, this residual interaction was dependent on the K90–F96 segment of Rhino (Fig. 4G–J). Therefore, we hypothesized that bridging might still occur in the absence of the K13–F18 segment, which contains the first KYxxL+ motif.

To test this hypothesis, we constructed an alternative dimeric model of the 9–1–1 complex using the Rhino R41–S104 peptide, in which Rhino connects Rad1 of the first 9–1–1 complex with Rad9 of the second complex. Throughout a 1 μs MD simulation, the R41–S104 segment of Rhino maintained stable interactions, and the binding interfaces with Rad1 and Rad9 were preserved relative to their initial conformations (Fig. 6J, N–P). The RMSD of all subunits, including Rhino, plateaued, indicating a stable conformational state (Fig. 6K–M). These results indicate that Rhino R41–S104 can bridge Rad1 and Rad9 of two distinct 9–1–1 complexes, even in the absence of its N-terminal Rad9-binding motif.

This structural configuration suggests a mechanism for higher-order assembly of the 9–1–1 complex. In the modeled dimer, the Rad9 front pocket of the first 9–1–1 complex remains accessible for interaction with another 9–1–1 complex. This implies that Rhino can sequentially link Rad1 of one complex to Rad9 of the next, forming a repeating array of 9–1–1 dimers. Such iterative bridging may facilitate the formation of a linear polymer of 9–1–1 complexes along the chromatin.

### *In silico* analysis of the association of the Rad9 C-terminal tail with the Rad9 front pocket and the iVERGE-binding surface on Hus1

**Figure 7.**
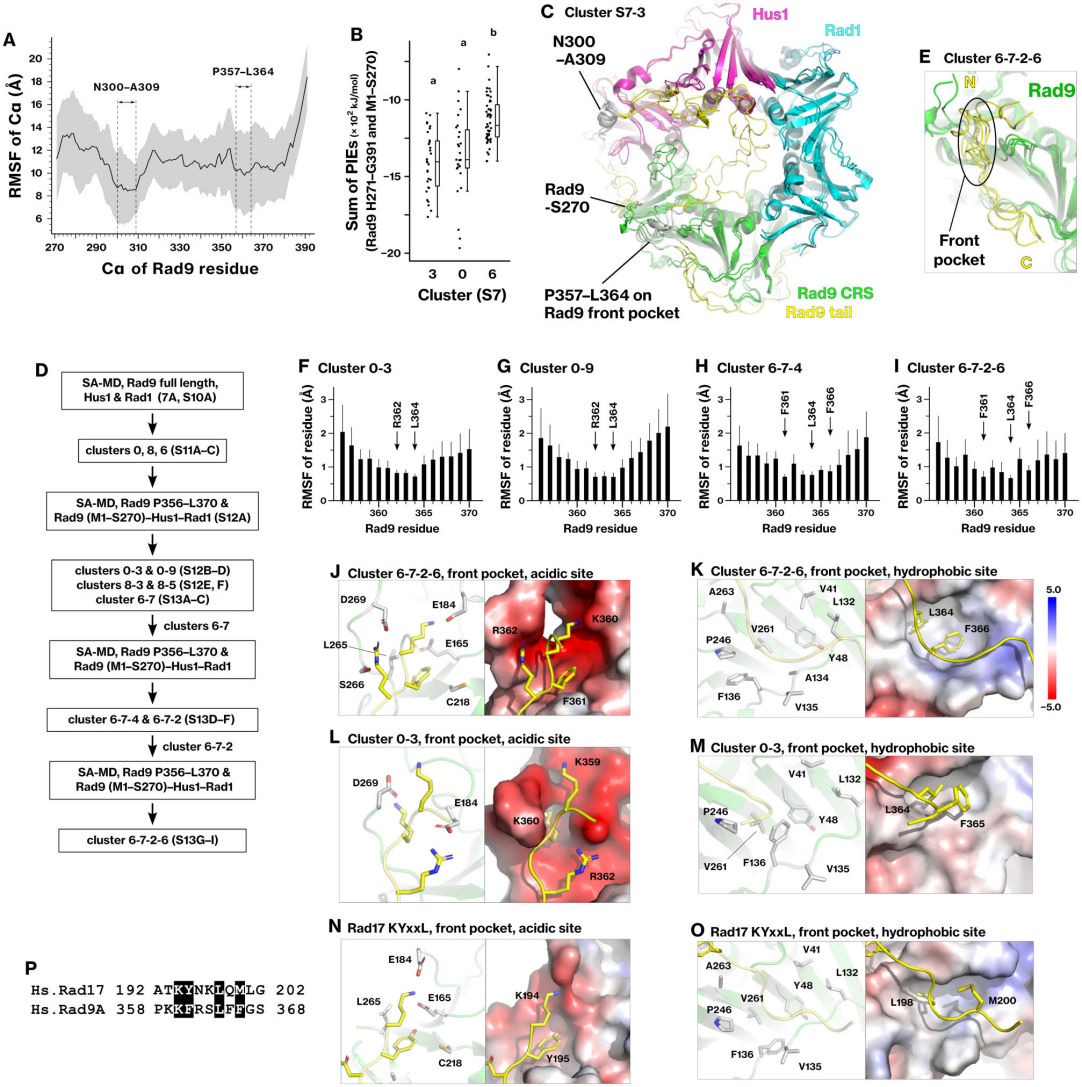
The Rad9 tail engages the Rad9 front pocket via two structurally distinct modes. (**A**) The association between the Rad9 tail and the core-ring structure of the 9–1–1 complex was analyzed using the SA-MD simulations (Supplementary Fig. S9A). RMSFs were calculated for Rad9 residues H271–G391 and averaged across 13 repeats of the productive run. The black line and gray area indicate the mean and standard deviation. (**B**) Dot plot showing the total PIE for stable clusters identified in Supplementary Fig. S9. (**C**) Main chains of the Rad9 tail (yellow), Rad9 M1–S270 (green), Hus1 (magenta), and Rad1 (cyan) are shown. Energetically stable conformations within cluster 3 from panel (B) were selected. (**D**) The conformation of the Rad9 tail was further refined according to the indicated scheme. Clusters 0–3, 0–9, 6–7–4, and 6–7–2–6 were identified as stable. Details are described in Supplementary Results 2. (**E**) Main chains of the Rad9 tail (yellow) and Rad9 M1–S270 (green) are shown for cluster 6–7–2–6. (**F**–**I**) RMSFs were calculated for Rad9 K359–F366 and averaged within each cluster. Thick and thin bars represent the mean and standard deviation, respectively. (**J**–**M**) The Rad9 tail (yellow) in the Rad9 front pocket (green) is illustrated for the most stable conformations in clusters 6–7–2–6 and 0–3. (**N, O**) The Rad17 KYxxL motif (yellow) on the Rad9 front pocket (green) is depicted in the experimental structure (PDB 7Z6H). (**P**) Sequence alignment of the Rad17 KYxxL motif and Rad9 P358–S368. The corresponding PIEs and binding free energies are shown in Tables 5–7, and the structures are provided in Supplementary PDB files 5 and 6.

Previous biochemical studies have demonstrated that the C-terminal tail of Rad9 interacts with the core-ring structure of the 9–1–1 complex [63, 64]; however, no structural data are available to explain the molecular details of this interaction. We examined the association of the Rad9 C-terminal tail with the core-ring structure using *in silico* modeling, as described in the “Materials and methods” section, under Conformational sampling of the Rad9 tail on the core-ring structure of the 9–1–1 complex. Analysis of the RMSFs of the Rad9 tail (H271–G391) revealed a stable region spanning residues N300–A309 (Fig. 7A). In addition, residues P357–L364 exhibited a distinct trough in the RMSF profile. This result raised the possibility that the Rad9 tail has specific interaction sites with the core-ring structure.

Before refining the individual binding interfaces, we first explored the overall stable conformations of the Rad9 C-terminal tail. Among the sampled conformations, clusters 0 and 3 were identified as the most stable based on the PIE analysis (Fig. 7B and Supplementary Fig. S10B–D). In these representative conformations, the Rad9 tail interacted with Hus1 and Rad9 (Fig. 7C). Specifically, P357–L364 residues were located in the Rad9 front pocket, consistent with previous biochemical studies [63, 64]. Additionally, the N300–A309 residues were associated with the outer surface of Hus1 (Fig. 7C), which is the same interface utilized by the Rad17-iVERGE and RAD24 C-terminus [6, 19]. These findings suggest that specific segments of the Rad9 tail stably interact with both Rad9 and Hus1.

### Conformational sampling of Rad9 P356–L370 bound to the Rad9 front pocket

To characterize the binding conformation of the Rad9 tail at the Rad9 front pocket, we focused on residues P356–L370. We refined the structure of the Rad9 P356–L370 residues in the Rad9 front pocket using SA-MD simulations and PIE analysis (Fig. 7D). Four final clusters, 0–3, 0–9, 6–7–4, and 6–7–2–6, were identified as stable and statistically independent. Details of the conformational sampling and optimization are described in Supplementary Results 2 and Supplementary Figs S10–S13.

### Rad9 P356–L370 adopts two distinct binding conformations, one recapitulating the KYxxL motif interaction

We evaluated the residue-level basis for Rad9 tail binding at the front pocket. K359, K360, and R362 residues exhibited the strongest interaction energies across all four clusters through electrostatic interactions (Tables 5 and 6; Supplementary Fig. S13D and E). In contrast, F361, L364, and F366 residues contributed to binding primarily through dispersion and solvation, suggesting van der Waals and hydrophobic interactions. Among them, L364 displayed the lowest RMSF across all clusters (Fig. 7F–I), indicating its role as a central anchoring residue. Supporting this conclusion, previous biochemical studies have demonstrated that the K359A/K360A/R362A or L364A mutations abolished the interaction between the Rad9 tail and the core-ring structure [63]. Together, these findings identified a set of key residues: K359, K360, R362, and L364.

**Table 5.** PIEs between the Rad9 K359‒F366 and M1‒S270, corresponding to the interactions shown in Fig. 7

Rhino residue	Cluster 0–3	Cluster 0–9	Cluster 6–7–4	Cluster 6–7–2–6
**K359**	**−166 ± 49**	**−128 ± 30**	**−160 ± 34**	−32 ± 36
**K360**	**−206 ± 63**	**−178 ± 37**	−54 ± 55	**−161 ± 46**
F361	20 ± 11	10 ± 17	−14 ± 8	−10 ± 5
**R362**	**−165 ± 20**	**−209 ± 21**	**−91 ± 66**	**−99 ± 52**
L364	−15 ± 7	−16 ± 9	−15 ± 6	−18 ± 6
F365	−2 ± 4	−8 ± 6	−3 ± 5	2 ± 5
F366	−6 ± 9	−3 ± 8	−13 ± 8	−10 ± 2

The *PIE*_*i,j*_ for each Rad9 residue (*i*) was summed across conformations, and the means and standard deviations were calculated for clusters 0–3, 0–9, 6–7–4, and 6–7–2–6, as defined in Fig. 7 and Supplementary Fig. S13. All values are in kJ/mol.

**Table 6.** PIEs between Rad9 K359‒F366 and M1‒S270, corresponding to the interactions shown in Fig. 7

Rhino residue	PIE	*E*es	*E*disp	*G*sol
Cluster 6–7–2–6				
K359	−32 ± 36	−136 ± 56	−1 ± 1	106 ± 28
K360	−161 ± 46	−257 ± 45	−7 ± 2	104 ± 8
**F361**	**−10 ± 5**	9 ± 3	**−15 ± 2**	**−4 ± 4**
R362	−99 ± 52	−172 ± 35	−8 ± 3	80 ± 16
**L364**	**−18 ± 6**	0 ± 4	**−13 ± 1**	**−5 ± 4**
**F366**	**−10 ± 2**	4 ± 2	**−12 ± 3**	**−3 ± 2**
Cluster 0–3				
K359	−166 ± 49	−316 ± 28	−7 ± 4	156 ± 24
K360	−206 ± 63	−330 ± 45	−13 ± 4	136 ± 24
**R362**	**−165 ± 20**	−264 ± 11	**−12 ± 2**	110 ± 12
**L364**	**−15 ± 7**	3 ± 3	**−16 ± 1**	**−3 ± 6**
F365	−2 ± 4	1 ± 2	−6 ± 3	3 ± 3
F366	−6 ± 9	−7 ± 4	−4 ± 2	4 ± 8

The *PIE*_*i,j*_, *E*es_*i,j*_, *E*disp_*i,j*_, and *G*sol_*i,j*_ for each Rad9 residue were summed across conformations, and the means and standard deviations were calculated for clusters 6–7–2–6 and 0–3, as defined in Fig. 7. All values are in kJ/mol.

We compared the four clusters and classified them into two groups: clusters 0–3 and 0–9, and clusters 6–7–4 and 6–7–2–6. In the first group (0–3 and 0–9), R362 and L364 exhibited the lowest RMSF (Fig. 7F and G). R362 interacted with the acidic subpocket (Fig. 7L and Supplementary Fig. S14E and G), whereas L364 and F365 interacted with the hydrophobic subpocket (Fig. 7M and Supplementary Fig. S14F and H). In the second group (6–7–4 and 6–7–2–6), F361, L364, and F366 were the most stable (Fig. 7H and I). K360 and F361 interacted with the acidic subpocket (Fig. 7J and Supplementary Fig. S14A and C), whereas L364 and F366 interacted with the hydrophobic subpocket (Fig. 7K and Supplementary Fig. S14B and D). These interaction profiles align with previous biochemical findings that the F365A/F366A mutations abolish the interaction between the Rad9 C-terminal tail and the core-ring structure [64]. These findings revealed that the Rad9 tail adopts two alternative stabilization modes through distinct anchoring residues.

We compared the optimized conformations of the Rad9 tail with the experimentally determined structure of the Rad17 KYxxL motif bound to the Rad9 front pocket (PDB 7Z6H) [5]. Notably, the conformation observed in cluster 6–7–2–6 closely resembled this crystal structure (Fig. 7N and O), with Rad9 residues K360–F366 aligning with the corresponding Rad17 residues K194–M200 (Fig. 7P). These findings validate the SA-MD- and FMO-derived Rad9 tail conformations by demonstrating their agreement with the experimentally determined structure and known biochemical characteristics of the Rad17 KYxxL motif [5, 18, 63, 64].

### Two most stable conformations of the Rad9 C-terminal are energetically equivalent

For clusters 0–3, 0–9, 6–7–4, and 6–7–2–6, the final binding free energy, *ΔG*_bind_, between Rad9 P356–L370 and the Rad9 core-ring structure was calculated (Table 7). Among them, clusters 6–7–2–6 and 0–3 exhibited the strongest and comparable binding energies. This result indicates that the KYxxL-like conformation in cluster 6–7–2–6 and the alternative conformation represented by cluster 0–3 are energetically equivalent, suggesting that the Rad9 tail associates with the front pocket through two distinct yet comparable stable binding modes.

**Table 7.** Binding free energy of the Rad9 T355‒A371 segment bound to the core-ring structure (M1‒S270), corresponding to the interactions shown in Fig. 7

Cluster	*ΔG*_bind_ (kJ/mol)
0–3	−437
0–9	−269
6–7–4	−345
6–7–2–6	−461

The binding free energy (*ΔG*_bind_) was calculated using the FMO3–DFTB3/PCM method as described in the “Materials and methods” section under Calculation of the final binding free energy of a ligand–receptor complex. CRS, core-ring structure.

### Rad9 C-terminal tail engages a basic–hydrophobic groove on Hus1 through conserved acidic and hydrophobic residues

**Figure 8.**
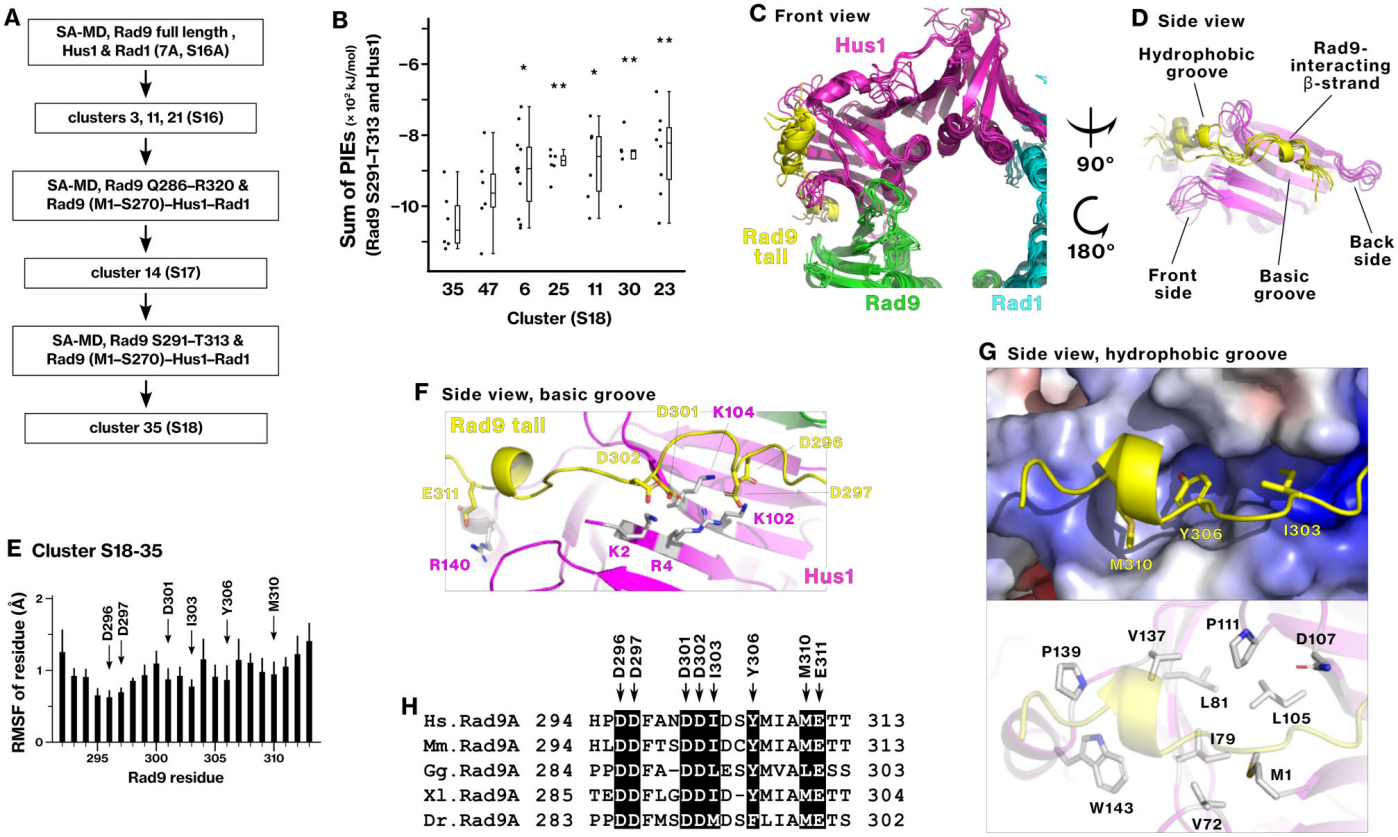
Rad9 C-terminal tail engages a basic–hydrophobic groove on the outer side of Hus1. (**A**) The conformation of the Rad9 tail on Hus1 was analyzed according to the indicated scheme. Details are described in Supplementary Results 3. (**B**) Dot plot showing the total PIE for each conformation within the clusters (Supplementary Fig. S17B and C). Statistical significance was assessed relative to cluster 35 (S18–35) using Student’s or Welch’s t-test. * *P* < 0.05; ** *P* < 0.01. (**C, D**) The main chains of the Rad9 C-terminal tail (S291–T313, yellow) and Hus1 in cluster 35 are shown. (**E**) RMSFs were calculated for Rad9 S291–T313 residues and averaged within cluster 35. Thick and thin bars represent the mean and standard deviation, respectively. (**F, G**) The most stable conformation in cluster 35 is depicted. Hus1 and Rad9 S291–T313 are shown in magenta and yellow, respectively. (**H**) Multiple sequence alignment of the Hus1-binding region in the Rad9 C-terminal tail. The corresponding PIEs are shown in Tables 8 and 9, and the structure is provided in Supplementary PDB file 7.

We investigated how the C-terminal tail of Rad9 interacts with Hus1 by reanalyzing the trajectory of the SA-MD simulations involving full-length Rad9, Hus1, and Rad1. Details of the conformational sampling and optimization are provided in Supplementary Results 3 (Fig. 8A and Supplementary Figs S15–S17). During the iterative refinement, unstable peripheral residues were excluded, allowing us to narrow down the Hus1-interacting core region to Rad9 residues S291–T313. We identified cluster 35 as the most stable cluster (Fig. 8B).

We analyzed the most stable conformational cluster, cluster 35, in which Rad9 residues D296–E311 engaged both the basic and hydrophobic grooves on the outer surface of Hus1 (Fig. 8C and D and Supplementary Fig. S18A). Most of the interaction energy arose from electrostatic interactions between the basic residues of Hus1 and acidic residues of Rad9 D296–E311 (Tables 8 and 9). The RMSF analysis indicated that the Rad9 peptide was anchored by several residues (Fig. 8E). Rad9-D296, D297, D301, and D302 interacted with the basic groove of Hus1 (Fig. 8F). Rad9-I303, Y306, and M310 were engaged with the hydrophobic groove of Hus1 (Fig. 8G). The Hus1-interacting residues in Rad9 are conserved across vertebrates (Fig. 8H). These results define Rad9 S291–T313 as a stable Hus1-binding segment. The final binding energy, *ΔG*_bind_, for the interaction of Rad9 S291–T313 with Hus1 was calculated to be −635 kJ/mol (Table 12).

**Table 8.** PIEs between Hus1 M1–S280 and Rad9 S291‒T313, corresponding to the interactions shown in Fig. 8

Hus1 residue	PIE (kJ/mol)
K102	−284 ± 20
K104	−265 ± 30
R4	−225 ± 35
K2	−153 ± 39
M1	−151 ± 35
R140	−124 ± 40
K108	−91 ± 22

The *PIE*_*i,j*_ for each Hus1 residue was summed across conformations, and the means and standard deviations were calculated for cluster S18–35, as defined in Fig. 8.

**Table 9.** PIEs between Rad9 S291‒T313 residues and Hus1 M1‒S280, corresponding to the interactions shown in Fig. 8

Rad9 residue	PIE	*E*es	*E*disp	*G*sol
D296	**−223 ± 22**	−231 ± 20	−8 ± 1	13 ± 3
D297	**−267 ± 26**	−284 ± 21	−8 ± 0	20 ± 12
D301	**−184 ± 16**	−205 ± 20	−9 ± 1	30 ± 6
D302	**−109 ± 10**	−125 ± 13	−6 ± 2	22 ± 8
I303	−23 ± 5	−17 ± 5	**−12 ± 2**	6 ± 2
Y306	−9 ± 6	−1 ± 5	**−9 ± 3**	0 ± 4
M310	−8 ± 4	−1 ± 2	**−10 ± 2**	3 ± 1
E312	**−89 ± 24**	−93 ± 30	−4 ± 1	8 ± 14

The *PIE*_*i,j*_, *E*es_*i,j*_, *E*disp_*i,j*_, and *G*sol_*i,j*_ for each Rad9 residue were summed across conformations, and the means and standard deviations were calculated for cluster S18–35, as defined in Fig. 8. All values are in kJ/mol.

This binding interface of Hus1 is not exclusive to its interaction with the Rad9 tail. Our previous study showed that the same Hus1 residues are involved in binding Rad17 iVERGE [19]. The C-terminus of RAD24 also interacts with the same surface on MEC3 [6]. As discussed later, these observations suggest potential competition between the Rad9 tail and iVERGE for the same binding surface on Hus1.

### p21 H152–P164 adopts two distinct binding conformations on the Rad9 front pocket

**Figure 9.**
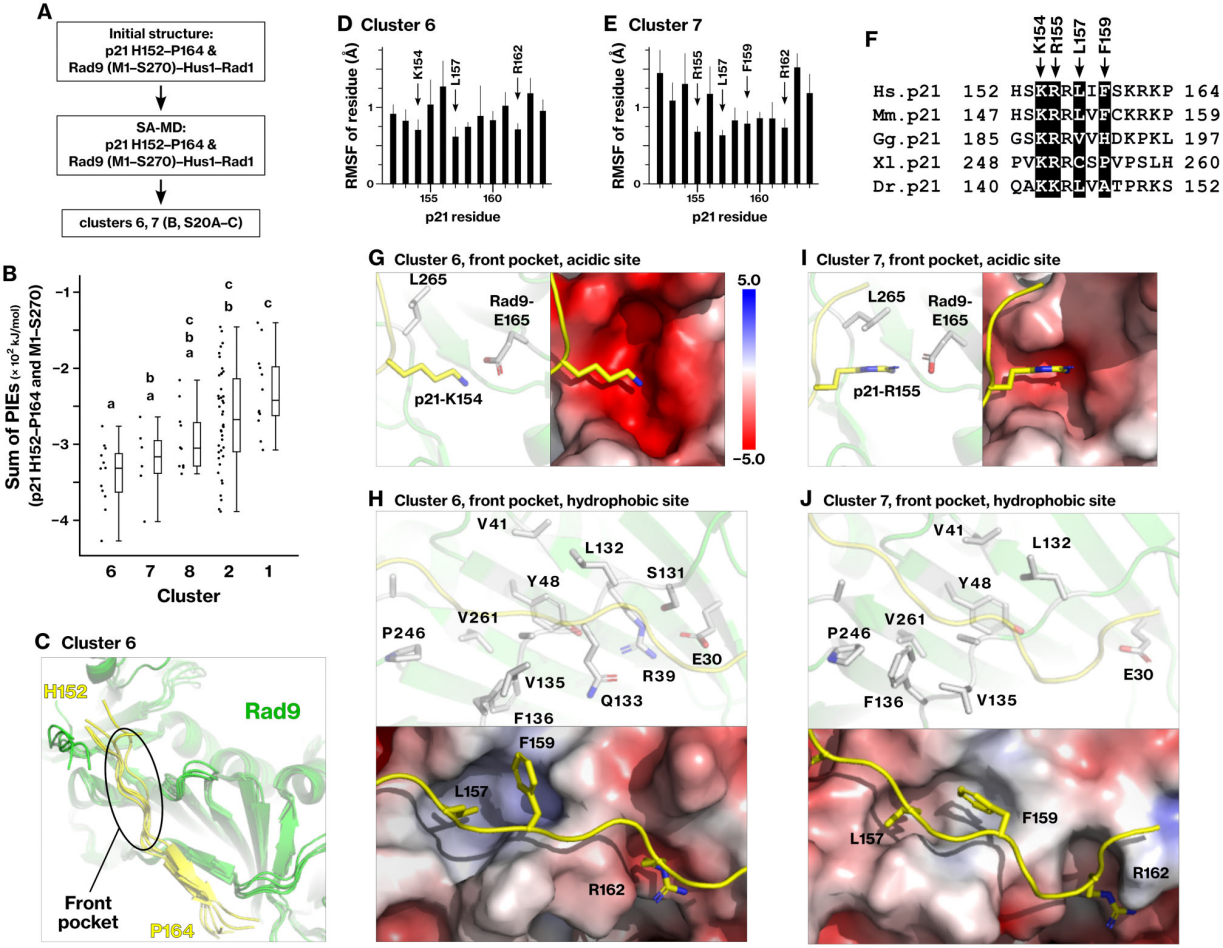
p21 residues H152–P164 adopt two distinct conformations on the Rad9 front pocket. (**A**) The association between p21 H152–P164 and the core-ring structure was analyzed according to the indicated scheme. (**B**) Dot plot showing the total PIE for each conformation. Clusters 6 and 7 were selected as the stable clusters (Supplementary Fig. S19A–C). (**C**) Main chains of p21 (yellow) and the Rad9 core-ring structure (green) are shown for stable conformations within cluster 6. (**D, E**) RMSFs for p21 residues were calculated and averaged within each cluster. Thick and thin bars represent the mean and standard deviation, respectively. (**F**) Multiple sequence alignment of p21 H152–P164 residues. (**G**–**J**) Structure of p21 (yellow) in the front pocket of Rad9 (green) is shown for the most stable conformations in clusters 6 and 7. The corresponding PIEs and binding free energies are presented in Tables 10 and 11, and the structures are provided in Supplementary PDB files 8 and 9.

We also examined the interaction between p21 and the 9–1–1 complex *in silico*. A previous biochemical study reported that the C-terminal region of p21 (residues 139–160) is associated with the core-ring structure of the 9–1–1 complex [11]; however, no structural data has been reported. AlphaFold2 predicted the association of p21 H152–P164 peptide with the Rad9 front pocket (Fig. 9A). Analysis of the PIEs identified cluster 6 as the most stable (Fig. 9B and Supplementary Fig. S19A and B), whereas clusters 7 and 8 were grouped with cluster 6 according to the Tukey–Kramer test (Supplementary Fig. S19C). Clusters 6 and 7 were selected for subsequent structural and energetic analyses.

p21 interacted with the Rad9 front pocket via two binding patterns observed in the Rad9 C-terminal tail (Fig. 9C). p21-K154 and R155 contributed the largest interaction energies in clusters 6 and 7, respectively (Table 10), and both residues interacted with the acidic subpocket (Fig. 9G and I). In both clusters, L157 and F159 contributed interaction energy dominated by dispersion and solvation (Table 10) and occupied the hydrophobic subpocket (Fig. 9H and J). Notably, p21-L157 exhibited the lowest RMSF (Fig. 9D and E), suggesting that L157 stabilized p21 on the front pocket. Consistent with the interaction energy and stability profiles, p21-K154, R155, and L157 are conserved among the vertebrate p21 sequences (Fig. 9F). This interaction pattern is consistent with recent biochemical data showing that the human p21-I158A/F159A double mutant was deficient in binding to the core-ring structure of the 9–1–1 complex [63]. These findings indicate that p21 binds to the Rad9 front pocket through two distinct conformations represented by clusters 6 and 7, and employs a binding mode shared with the Rad9 C-terminal tail and Rhino-derived peptides, later referred to as the KYxxL+ motif.

**Table 10.** PIEs between p21 H152‒P164 and Rad9 M1–S270, corresponding to the interactions shown in Fig. 9

p21 residue	PIE	*E*es	*E*disp	*G*sol
Cluster 6				
**K154**	**−128 ± 14**	−186 ± 17	−12 ± 2	71 ± 15
R155	−64 ± 51	−130 ± 52	−5 ± 4	72 ± 17
R156	−36 ± 11	−67 ± 18	−6 ± 1	37 ± 14
**L157**	**−22 ± 2**	−2 ± 3	**−13 ± 1**	**−8 ± 2**
**F159**	**−18 ± 2**	−3 ± 1	**−14 ± 2**	−1 ± 1
K161	−39 ± 19	−68 ± 21	−6 ± 2	34 ± 11
**R162**	**−97 ± 15**	−102 ± 27	−13 ± 4	16 ± 17
Cluster 7				
K154	−50 ± 53	−120 ± 49	−4 ± 4	73 ± 25
**R155**	**−136 ± 14**	−178 ± 19	−15 ± 2	59 ± 16
R156	−52 ± 33	−91 ± 40	−6 ± 2	45 ± 16
**L157**	**−20 ± 7**	3 ± 3	**−15 ± 1**	**−9 ± 5**
**F159**	**−16 ± 3**	−2 ± 2	**−14 ± 2**	0 ± 2
K161	−66 ± 40	−102 ± 37	−8 ± 2	44 ± 5
**R162**	**−78 ± 14**	−75 ± 17	−13 ± 2	9 ± 14

The *PIE*_*i,j*_, *E*es_*i,j*_, *E*disp_*i,j*_, and *G*sol_*i,j*_ for each p21 residue were summed across conformations, and the means and standard deviations were calculated for clusters 6 and 7, as defined in Fig. 9. All values are in kJ/mol.

The final binding energies between p21 and Rad9 were calculated to be −390 and −415 kJ/mol for clusters 6 and 7, respectively (Table 11), indicating that these conformations are energetically comparable. These structural and thermodynamic data define two comparable p21–Rad9 binding conformations stabilized by a conserved interaction motif.

**Table 11.** Binding free energy between p21 H152‒P164 and the Rad9 core-ring structure (M1‒S270), corresponding to the interactions shown in Fig. 9

Cluster	*ΔG*_bind_ (kJ/mol)
6	−390
7	−415

The binding free energy (*ΔG*_bind_) was calculated using the FMO3-DFTB3/PCM method as described in the “Materials and methods” section under Calculation of the final binding free energy of a ligand–receptor complex.

### Identification of the KYxxL+ motif as a conserved Rad9-interaction element across multiple binding proteins

**Figure 10.**
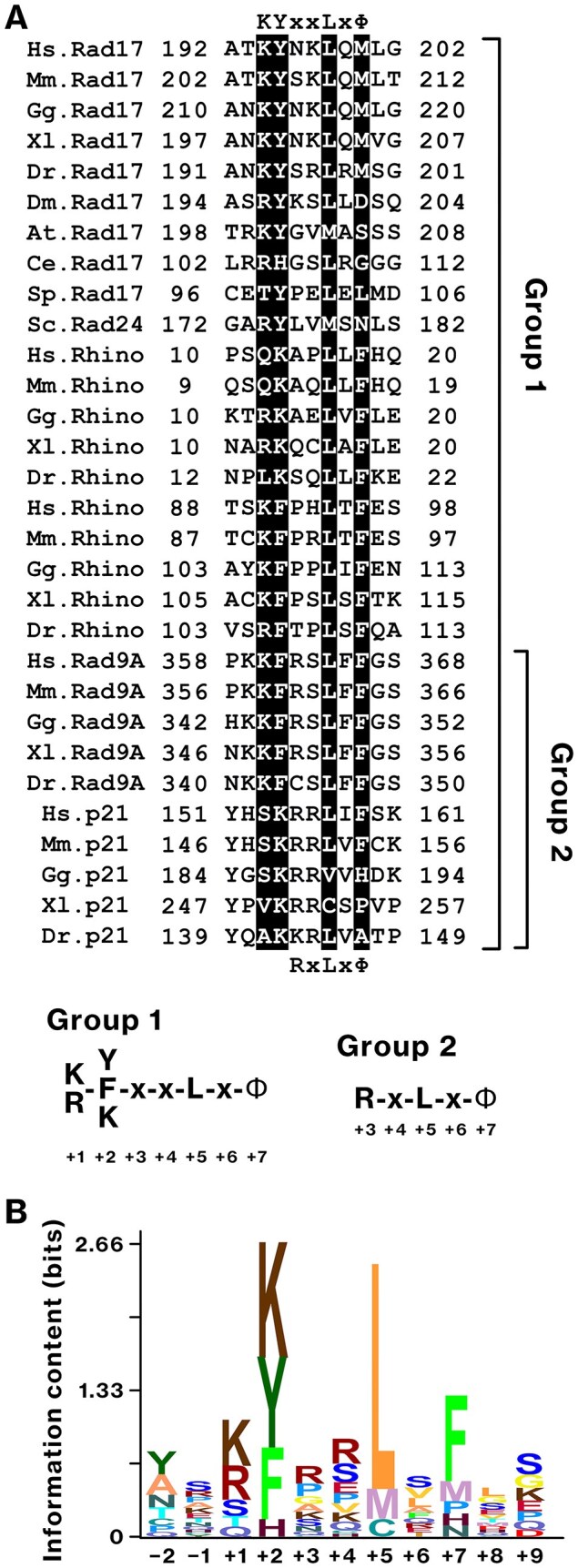
Multiple sequence alignment reveals two subgroups of the KYxxL+ motif with distinct interactions at the Rad9 front pocket. (**A**) Multiple sequence alignment of the Rad9-binding regions in Rhino, the Rad17 KYxxL motif, the Rad9 C-terminal tail, and p21. Conserved residues in Group 1 are shown as white letters on a black background. A subset of the sequences sharing the RxLxΦ consensus is classified as Group 2. Hs, *Homo sapiens*. Mm, *Mus musculus*. Gg, *Gallus gallus*. Xl, *Xenopus laevis*. Dr, *Danio rerio*. Dm, *Drosophila melanogaster*. At, *Arabidopsis thaliana*. Ce, *Caenorhabditis elegans*. Sc, *Saccharomyces cerevisiae*. Sp, *Schizosaccharomyces pombe*. (**B**) A profile HMM was constructed based on the alignment, and the corresponding HMM logo was shown.

Multiple sequence alignments of the Rad17 KYxxL motif and other Rad9-binding regions revealed a conserved sequence pattern among human Rhino Q12–F18, K90–F96, Rad9 K360–F366, and p21 S153–F159 (Fig. 10A). To further characterize this conserved pattern, we constructed a profile HMM (Fig. 10B). We refer to this motif as the KYxxL+ motif, as it represents an expansion of the original Rad17 KYxxL motif.

Our analysis indicated that leucine at position +5 was the most conserved residue among all aligned sequences (Fig. 10A). Structural analysis indicated that this leucine anchors stably to the hydrophobic subpocket of the Rad9 front pocket primarily via hydrophobic and van der Waals interactions, as supported by RMSF and PIEDA analyses (Figs 1, 3, 7, and 9; Tables 1, 3, 6, and 10). The hydrophobic residues at position +7 were highly conserved, whereas those at position +6 were more variable. However, in some cases, such as in the Rad9 C-terminal tail, a hydrophobic residue at position +6 functionally substitutes for the residue at position +7 (Fig. 7M). Together, these results define a conserved hydrophobic core within the KYxxL+ motif, anchored by leucine at the position +5 and stabilized by the surrounding variable residues.

The KYxxL+ motif adopts two distinct interaction modes with the acidic subpocket of the Rad9 front pocket. Group 1, the predominant mode, follows the consensus K/R–Y/F/K–x–x–L–x–Φ, where Φ denotes a hydrophobic residue. The lysine at position +1 is functionally dispensable if the position +2 is occupied by a basic residue, as observed for p21 and Rhino K13–F18. These residues at +1 and +2 interact with the acidic subpocket of Rad9 (Figs 1, 3, 7, and 9). Group 2, by contrast, is defined by the consensus R–x–L–x–Φ. It features an arginine at position +3 that engages the acidic subpocket instead of the residues at positions +1 or +2 (Figs 7 and 9). These observations indicate that the KYxxL+ motif employs two structurally distinct but functionally equivalent strategies to engage the acidic subpocket. Together, these results establish the KYxxL+ motif as a conserved module mediating interaction with Rad9, anchored by a conserved hydrophobic core and modulated by flexible engagement of the acidic subpocket.

### Summary of binding free energies of the 9–1–1 complex and its ligands

The final binding energies between the 9–1–1 complex and its ligands are summarized (Table 12). These values provide semi-quantitative estimates of the relative binding free energies among candidate complexes. We previously reported that the Rad17 KYxxL motif and its surrounding peptides exhibited a binding free energy (*ΔG*_bind_) of −1327 kJ/mol in the interaction with the front pocket of Rad9 [19], which was calculated based on the experimental structure of the Rad17–RFC2–5 and 9–1–1 complexes [5]. It showed the largest binding energies among all tested ligands. At the front pocket of Rad9, Rhino P10–A31 (the first KYxxL+ motif), Rhino S83–S98 (the second KYxxL+ motif), and Rad9 T335–A371 showed comparable binding energies, suggesting that these ligands are interchangeable, except for the Rad17 KYxxL motif. In contrast, on the Rad1 surface, Rhino exhibited a stronger binding affinity than Rad17, suggesting that Rhino is capable of replacing Rad17 at this binding interface. On the Hus1 surface, Rad17-iVERGE and Rad9 S291–T313 showed similar binding energies, suggesting potential interchangeability between these ligands.

**Table 12.** Binding free energies of the ligand peptides to the core-ring structure of the 9‒1‒1 complex

Ligand and receptor	*ΔG*_bind_ (kJ/mol)	Buried surface area (Å^2^)	Structure used for the calculation	Reference
Rad17 KYxxL & Rad9 CRS	−1327	1041	PDB 7Z6H	[Ref. 5, 19]
Rhino P10‒A31 & Rad9 CRS	−610	1417	Fig. 1	This study
Rhino S83‒S98 & Rad9 CRS	−515	981	Fig. 3	This study
Rad9 T355‒A371 & Rad9 CRS	−461	934	Fig. 7	This study
p21 & Rad9 CRS	−415	993	Fig. 9	This study
Rad17 & Rad1	−293	707	PDB 8GNN	[Ref. 20, 19]
Rhino T38‒I48 & Rad1	−482	812	Fig. 2	This study
Rhino T52‒F61 & Rad1	−381	945	Fig. 2	This study[Ref. 26]
Rad17-iVERGE & Hus1	−693	627	[Ref. 20]	[Ref. 20]
Rad9 S291‒T313 & Hus1	−635	956	Fig. 8	This study
RFC2 & Rad9/Rad1	41	403	PDB 7Z6H	[Ref. 5, 19]
RFC5 & Rad1	8	706	PDB 7Z6H	[Ref. 5, 19]
RFC4 & Rad1	93	286	PDB 7Z6H	[Ref. 5, 19]

The binding free energy (*ΔG*_bind_) was calculated as *ΔG*_bind_ = *G*_complex_ – (*G*_receptor_ + *G*_ligand_). The structures of the complexes were optimized using the FMO2-DFTB3/PCM method, and the free energies of the receptor (*G*_receptor_), ligand (*G*_ligand_), and complex (*G*_complex_) were subsequently calculated using the FMO3-DFTB3/PCM method. The complex structures used for the FMO3 calculations are provided in the Supplementary PDB files. CRS, core-ring structure.

## Discussion

Although the Rad17–RFC2–5 and 9–1–1 complexes are well known for their roles in the activation of ATR-DDR, current knowledge remains largely limited to this activation phase, and their functions in the subsequent processes are still poorly understood. Our computational and biochemical analyses suggest regulatory mechanisms that govern Rad17 and the 9–1–1 complex, in conjunction with Rhino, during the transition from activation to maintenance and inactivation of ATR-DDR.

Rhino has been identified as a component of the ATR-dependent DNA damage and replication checkpoints [24, 25]; however, its molecular function remains poorly understood. One factor complicating the characterization of Rhino is that most of its sequence comprises intrinsically disordered regions. This study reveals that the N-terminal half of Rhino adopts specific conformations upon association with the 9–1–1 complex. These conformations enabled semi-quantitative structure–function relationship analyses, leading to several novel findings and hypotheses. These include the identification of the KYxxL+ motif, a model describing energetically equivalent transitions in the complex, a hypothesis of 9–1–1 polymerization, and the concept of dissociation-mediated inactivation, as discussed below.

Building on the structural insights obtained in this study, we reexamined how the 9–1–1 complex recognizes its client proteins. Previous studies have implied that the PIP-box, a PCNA-interacting motif, might mediate interactions with the 9–1–1 complex. However, we investigated the KYxxL+ motif as a common motif among Rad9-interacting proteins and redefined client specificity in the 9–1–1 complex. A major difference is the presence of basic residues that interact with the acidic subpocket.

**Figure 11.**
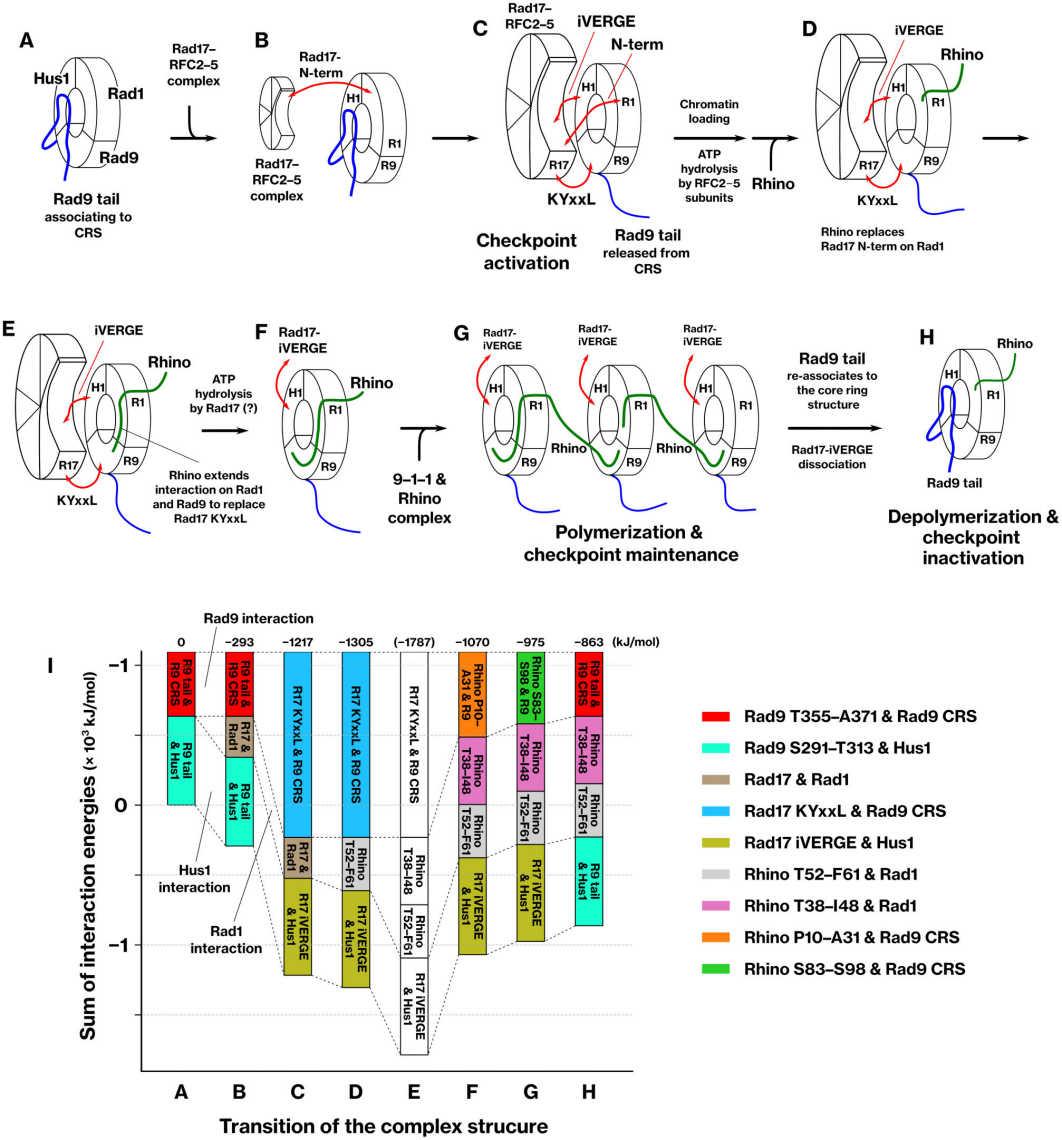
Rhino induces energetically neutral remodeling of the Rad17–9–1–1 complex. Changes in protein–protein interactions on the 9–1–1 complex were inferred from its interactions with ligand peptides. The pairs of interacting proteins are listed in Table 13. Details are described in the “Discussion” section. (**A**–**C**) An increase in total binding energy promotes the association of Rad17 (red) with the 9–1–1 complex and the release of the Rad9 tail (blue), enabling its interaction with checkpoint mediators. (**D**–**F**) Following chromatin loading of the 9–1–1 complex, the KYxxL motif and the N-terminus of Rad17 are displaced by Rhino (green), and Rad17 becomes anchored to Hus1 via its iVERGE region. (**G**) Rhino K90–F96 interacts with Rad9 on the adjacent complex, bridging Rad1 and Rad9 and promoting polymerization of the 9–1–1 complexes. This polymerization helps maintain checkpoint signaling. (**H**) Upon checkpoint inactivation, the Rad9 tail competitively replaces Rhino K90–F96 and the iVERGE, leading to disassembly of the 9–1–1 polymer and checkpoint complex. (**I**) Binding free energies between ligand peptides and the 9–1–1 complex were calculated using the FMO3-DFTB3/PCM method and are listed in Table 12. These values were summed across the interfaces of each intermediate complex in steps A–H and presented relative to step A. The total binding energy in step E is hypothetical, as the simultaneous binding of the Rad17 KYxxL motif and Rhino T38–I48 may cause steric hindrance and reduce overall binding energy. CRS, core-ring structure.

We also examined the remodeling of this complex. The current model describes the role of Rad17 and the 9–1–1 complex in the activation phase of ATR-DDR, while the behavior of these proteins during the maintenance and inactivation phases remains to be elucidated. Our precise structural optimizations enabled semi-quantitative evaluation of the interactions between the 9–1–1 complex and its client proteins, revealing energetically equivalent transitions in the Rad17–9–1–1–Rhino complex (Fig. 11). Our results suggest the 9–1–1 complex undergoes remodeling through changes in client protein interactions during the maintenance and inactivation phases without significant changes in the total binding energy, as discussed below.

Our analysis of the structure–function relationship of Rhino prompted us to revisit the long-standing discrepancies between the current ATR-DDR model and experimental observations of Rad17 and Rad9 as foci under fluorescence microscopy. This discrepancy can be explained by our finding that Rhino contains two KYxxL+ motifs, enabling it to bridge multiple 9–1–1 complexes on chromatin (Fig. 6). This interaction forms the basis of the 9–1–1 polymer hypothesis, which proposes that the polymerized 9–1–1 complexes act as a structural scaffold that concentrates checkpoint mediators to sustain ATR-DDR signaling.

Based on these integrated structural and functional insights, the disassembly of the checkpoint complex during ATR-DDR inactivation was investigated. Although a clamp unloader has been identified for PCNA [65], no analogous enzymes have been identified for the 9–1–1 complex. Our results indicate that the Rad9 tail competes with Rhino and Rad17 for binding to the core-ring structure, resulting in their dissociation (Figs 7 and 8). This suggests that the inactivation of ATR-DDR is driven by the dissociation of the Rad9-based protein complex and disassembly of the 9–1–1 polymer, which disperses checkpoint mediators.

Taken together, our results suggest a fundamental mechanism underlying the transition from activation to maintenance and inactivation of ATR-DDR, involving transitions among thermodynamically equivalent states and the presence of multiple copies of Rad17 and 9–1–1 complexes. In the following sections, we discuss key structural and functional findings that clarify how Rhino contributes to the regulation of ATR-DDR through its interactions with the 9–1–1 complex and Rad17.

### Rhino functions as a structural modulator of the Rad17–Rad9–Hus1–Rad1 complex

We propose a model in which Rhino acts as a structural modulator that governs the key molecular mechanisms underlying ATR-DDR (Fig. 11). In this model, Rhino competes with Rad17 for binding to Rad9 and Rad1, thereby promoting the displacement of the Rad17–RFC2–5 complex while leaving Rad17-iVERGE associated with Hus1 (Fig. 11D–F). This structural rearrangement represents the transition from the activation to the maintenance phase of ATR-DDR.

The maintenance phase is characterized by the association of Rhino with the 9–1–1 complex. The presence of two KYxxL+ motifs in Rhino suggests the potential for polymerization of the 9–1–1 complex on damaged chromatin. Under the 9–1–1 polymer hypothesis, Rhino connects multiple 9–1–1 complexes, stabilizing the checkpoint signaling platform (Fig. 11G). This polymerized state is subsequently resolved when the C-terminal tail of Rad9 competes for the same binding surfaces, leading to the displacement of Rhino and disassembly of the 9–1–1 polymer (Fig. 11H). Under this hypothesis, the second KYxxL+ motif is displaced by the Rad9 tail, and the inactivation of ATR-DDR is represented by the dissociation of Rhino and the disassembly of the 9–1–1 polymer.

In contrast, if the 9–1–1 polymer hypothesis is not assumed, the first KYxxL+ motif is displaced by the Rad9 tail, leading to the dissociation of Rhino from the 9–1–1 complex and inactivation of the checkpoint. In this scenario, the functional role of the second motif remains unexplained.

Together, these results integrate the activation, maintenance, and inactivation phases into a unified framework explained by the state transitions of the 9–1–1 complex. In the following sections, we describe the structural and biochemical evidence supporting each step of this model.

### Remodeling of the Rad17–Rad9–Hus1–Rad1–Rhino complex through energetically equivalent structural transitions

Interaction energy calculations using the FMO method enabled semi-quantitative thermodynamic analysis of the protein complexes between the 9–1–1 complex and its client proteins. The total binding free energies (*ΔG*) of all ligands on the 9–1–1 complex were comparable across structural intermediates (Fig. 11I). This suggests that the replacement of Rad17 by Rhino or Rhino by the Rad9 tail can occur without substantial energetic penalty. These findings suggest that the 9–1–1 complex is remodeled through transitions among structural states that are functionally distinct yet energetically equivalent, underpinning the progression from activation to maintenance and inactivation phases of the checkpoint response.

### Previous biochemical data suggest the presence of multiple copies of Rad17 and 9–1–1 complexes at DNA damage sites and support the 9–1–1 polymer hypothesis

Conventional models of ATR activation depict a single molecule of the Rad17–RFC2–5 and 9–1–1 complexes placed at the junction of single- and double-stranded DNA. However, Rad17 and the 9–1–1 complex were observed as foci under fluorescence microscopy. Since these foci represent clusters containing multiple copies of Rad17 or Rad9 complexes at DNA damage sites, the prevailing model cannot explain these experimental observations.

Rad17 and Rad9 foci have been reported under various conditions. Endogenous Rad17 has been reported to form nuclear foci after ultraviolet (UV) irradiation [66], ionizing radiation [67], and the induction of replication stress [68, 69]. Endogenous Rad17 has also been observed as foci of Rad17-S645 phosphorylation [13]. These observations suggest that multiple copies of endogenous Rad17 are phosphorylated by ATR and accumulate at the sites of DNA damage. Endogenous Rad9 was observed as foci after UV irradiation [70], double-strand breaks (DSBs) [70–72], and replication stress [70, 71, 73]. Exogenous Rad9 was observed as foci after UV irradiation [74, 75], DSBs [75], and replication stress [73, 74]. Strikingly, DDC1, a Rad9 homolog in *S. cerevisiae*, formed nuclear foci after induction of a single chromosomal DSB by the HO endonuclease [76, 77], indicating that Rad9 focus formation does not require multiple DNA lesions. These findings indicate that multiple copies of Rad17 and the 9–1–1 complex accumulate at damage sites; however, this feature has not been incorporated into prevailing ATR-DDR models.

Our structural and biochemical analyses, particularly the identification of two KYxxL+ motifs in Rhino, led us to hypothesize that Rhino mediates the polymerization of the 9–1–1 complex. In this model, Rad17 is incorporated into each 9–1–1 monomer through the iVERGE–Hus1 interaction (Fig. 11G). The 9–1–1 polymer hypothesis thus provides a potential explanation for the discrepancy between the experimental observations of Rad17 and Rad9 foci and the prevailing model, which assumes single units of Rad17 and Rad9.

### Free energy differences regulate client exchange on the 9–1–1 complex, driving Rad17 association through displacement of the Rad9 C-terminal tail

We identified a mechanism whereby the association of the Rad17–RFC2–5 complex with the 9–1–1 complex is facilitated by an increase in binding free energy. The 9–1–1 complex contains three major interaction sites: the front pocket of Rad9, the side groove of Rad1, and the basic and hydrophobic grooves of Hus1. These sites undergo client exchange as the checkpoint progresses through activation, maintenance, and inactivation (Fig. 11; Table 13).

**Table 13.** Association of ligands with the 9‒1‒1 complex during the checkpoint activation, maintenance, and deactivation

Checkpoint stages in Fig. 11	Default(A)	Rad17 association(C)	Rhino association(F)	Polymerize(G)	Depolymerize(H)
Rad9	Rad9 tail	Rad17 KYxxL	Rhino K13‒F18	Rhino K90‒F96	Rad9 tail
Hus1	Rad9 tail	iVERGE	iVERGE	iVERGE	Rad9 tail
Rad1	————	Rad17 N-term	Rhino	Rhino	Rhino
TopBP1-binding activity of Rad9 tail	off	on	on	on	off

The ligand peptides associated with Rad9, Hus1, and Rad1 are listed for each checkpoint phase, corresponding to panels A, C, F, G, and H in Fig. 11. The TopBP1-binding activities of the Rad9 tail were inferred from its association with the core-ring structure.

In the default state, the Rad9 front pocket and Hus1 groove are occupied by the Rad9 C-terminal tail, whereas the Rad1 side groove remains unoccupied (Fig. 11A). This configuration allows Rad17 to initially bind to Rad1 through its N-terminus without an energetic barrier (Fig. 11A and B; Table 13). Upon binding, Rad17 displaces the Rad9 tail through its KYxxL motif and iVERGE domain (Fig. 11C). On the Rad9 front pocket, the Rad17-KYxxL motif exhibits a binding energy of −1327 kJ/mol [19], substantially exceeding that of the Rad9 C-terminal tail, −461 kJ/mol. This energy difference provides the driving force for Rad17–RFC2–5 to replace the Rad9 tail and to associate with the 9–1–1 complex (Fig. 11I, columns B and C). These findings suggest that differences in the binding free energy promote the initial association of Rad17–RFC2–5 with the 9–1–1 complex.

### Rhino replaces Rad17 at the interfaces on Rad9 and Rad1 after the chromatin loading of the 9–1–1 complex

We identified the binding mode of Rhino on the 9–1–1 complex, which shows that its N-terminus competes with Rad17 for interaction sites on Rad9 and Rad1, suggesting that Rhino displaces the Rad17–RFC2–5 complex. This model is supported by structural and biochemical data indicating interactions mediated by the KYxxL+ motif in the Rhino K13–F18 segment (Figs 1 and 4), as well as by data involving the G25–C30 β-strand, T38–I48, and F52–F61 segments (Figs 1 and 2). These interactions position Rhino over substantial regions of the Rad9 and Rad1 surfaces (Fig. 1). Binding energy calculations show that Rhino engages these interfaces with an energy comparable to that of Rad17 [Rhino: −1473 kJ/mol (−610, −482, −381); Rad17: −1620 kJ/mol (−1327, −293); Table 12]. These structural and thermodynamic findings indicate that Rhino competitively replaces Rad17 at the binding interfaces on Rad9 and Rad1.

We propose that Rhino interacts with the 9–1–1 complex after its chromatin loading, replacing Rad17. Structural and biochemical data indicate that the Rad17–RFC2–5 complex recognizes the junction of single- and double-stranded DNA [5–9] and loads the 9–1–1 complex onto chromatin [12–14]. The Rad17–Rad9 interaction is essential for chromatin loading. However, whether this interaction is required for the subsequent checkpoint response remains to be determined. In contrast, Rhino is known to interact with TopBP1 [24, 25], implicating it in the later stages of the checkpoint response. These observations suggest that Rhino functions downstream of the initial Rad17–Rad9 interaction. Given their competitive nature, we suggest that Rhino replaces Rad17 on the 9–1–1 complex following chromatin loading (Fig. 11D).

We propose that ATP hydrolysis by Rad17 facilitates its replacement by Rhino. Previous studies have shown that Rad17 binds ATPγS in the context of the Rad17–RFC2–5 and 9–1–1 complex in both yeast and human systems, suggesting that ATP hydrolysis may facilitate the dissociation of Rad17 from the 9–1–1 complex [5–7]. In our previous work, the binding energy between the Rad17–RFC2–5 and 9–1–1 complexes was primarily attributable to Rad17, with no measurable contribution from the RFC2–5 subunits (Table 12) [19]. Simple dissociation of Rad17 requires the release of up to ~1200 kJ/mol of binding energy (Fig. 11I, columns A and C). By contrast, replacement by Rhino requires an energy change of only 147 kJ/mol, corresponding to the difference between the binding energies of Rad17 and Rhino (Fig. 11I, columns C and F; −1217 kJ/mol +1070 kJ/mol). Therefore, we propose that the ATP hydrolysis by Rad17 induces conformational changes that promote the replacement of Rad17 by Rhino as a more thermodynamically efficient pathway, rather than the simple dissociation without replacement (Fig. 11C–F).

Rhino is proposed to initially bind Rad1. This model is supported by the observation that the Rhino T52–F61 region exhibits comparable or stronger interaction energy with Rad1 than the corresponding region of Rad17 (Table 13; Fig. 11D). Upon initial binding of Rhino T52–F61 to Rad1, the upstream region T38–I48 is predicted to be inserted into the interface between Rad17 and the 9–1–1 complex. This can increase the total ligand interaction energy (Fig. 11E; 11I column E), providing a driving force for the insertion of Rhino. However, such an insertion may destabilize the association of Rad17, thereby buffering the increase in the total binding energy. ATP hydrolysis by RFC subunits has been proposed to promote the dissociation of the RFC2–5 subunits from the 9–1–1 complex [6], further facilitating Rhino entry. Together, these findings suggest that Rhino insertion is a thermodynamically driven process, leading to the functional remodeling of the 9–1–1 complex.

### Rad17 remains associated with the 9–1–1 complex through its iVERGE region

Our previous study suggested that Rad17 remains associated with the 9–1–1 complex through its iVERGE region, even after the dissociation of the Rad17 KYxxL motif. The iVERGE region interacts strongly with the 9–1–1 complex independently of the remainder of the Rad17 protein [19]. Notably, Rhino and iVERGE do not share overlapping binding interfaces on the 9–1–1 complex (Table 13), allowing the iVERGE to remain anchored within the Rhino–9–1–1 complex (Fig. 11F). In budding yeast, the C-terminus of RAD24 similarly interacts with MEC3 [6]. Previous studies have shown that Rad17 interacts with downstream checkpoint regulators such as claspin, indicating that Rad17 functions as a structural scaffold [78]. Taken together, these findings suggest that the iVERGE is instrumental in anchoring Rad17 within the Rhino–9–1–1 complex during the late phase of the checkpoint response.

### Context-dependent role of the second KYxxL+ motif supports the 9–1–1 polymer hypothesis

The distinct structural contexts of the two KYxxL+ motifs in Rhino suggest non-overlapping functions in the regulation of the 9–1–1 complex. The Rhino S83–S98 peptide showed a binding energy comparable to that of the Rhino P10–A31 peptide (Table 12) and remained stably associated with the Rad9 front pocket in the MD simulation (Fig. 5H–K). However, mutations in the residues S83–S93 did not affect 9–1–1 binding in the soluble fraction (Fig. 4A–D). This suggests that P10–A31 is the primary 9–1–1 binding interface. The P10–A31 segment is further stabilized by additional contacts involving residues T48–F61, whereas the S83–S98 peptide binds to Rad9 independently of these stabilizing interactions. This structural difference underlies the preferential engagement of the P10–A31 region. The double mutant showed no additional reduction in the co-precipitation of the 9–1–1 complex in the soluble fraction (Fig. 4C and D). These results indicate that the second KYxxL+ motif is not a functional substitute for the first but instead serves a distinct role, likely under specific spatiotemporal conditions.

We hypothesize that Rhino bridges two 9–1–1 complexes on damaged chromatin, thereby promoting polymerization of the 9–1–1 complex via Rhino (Fig. 11G). In the polymeric, rather than dimeric, form of the complex, Rhino T38–F61 engages Rad1, while S83–S98 binds to the Rad9 front pocket of a neighboring 9–1–1 complex (Figs 6J and 11G; Table 13). Such polymerization is likely induced during the checkpoint response on damaged chromatin.

The 9–1–1 polymer hypothesis was supported by our *in silico* analyses. The dimeric structure of the 9–1–1 complex remained stable in the absence of the first KYxxL+ motif, where Rhino bridges two 9–1–1 complexes through Rad1 and Rad9 (Fig. 6J–P). Furthermore, the binding energies of Rhino segments S83–S98 and P10–A31 were comparable (Table 12), indicating that polymerization does not alter the overall binding free energy between Rhino and 9–1–1 (Fig. 11, columns F and G). Together, these findings support the structural stability and energetic plausibility of the polymeric configuration.

The 9–1–1 polymer hypothesis is also consistent with biochemical data. This model accounts for the substantial interaction between the K13–F18 mutant and the 9–1–1 complex observed in the chromatin fraction (25%–38% of wild-type level; Fig. 4E–J), given that the polymerization configuration does not involve direct contact between the K13–F18 segment and Rad9 (Fig. 11G). Additionally, this hypothesis explains why the effect of the Rhino S83–S98 mutation becomes evident in the chromatin fraction (Fig. 4G–J), as the 9–1–1 polymer is likely strongly induced on the damaged chromatin. Furthermore, crystallographic data revealed an interaction between Rhino T88–S98 and Rad9 [63]. Therefore, the 9–1–1 polymer hypothesis provides a coherent explanation for the context-dependent functional importance of the second KYxxL+ motif (S83–S98).

The 9–1–1 polymer hypothesis also provides an explanation for previous biochemical observations reporting Rad17 and Rad9 foci formation under fluorescence microscopy [13, 66–77]. Given that the C-terminal regions of Rad17 and Rad24 directly interact with Hus1 and MEC3 [6, 19], the model suggests that Rad17 is associated with each monomer of the 9–1–1 polymer (Fig. 11G). We hypothesized that the polymeric 9–1–1 complex functions as a scaffold for recruiting and concentrating checkpoint mediators at sites of damage, thereby enhancing and sustaining ATR-DDR signaling. Current models of ATR-DDR do not account for the presence of multiple copies of Rad17 and Rad9, creating a discrepancy with the observed fluorescence foci, as discussed earlier. The 9–1–1 polymer hypothesis offers a coherent resolution to this long-standing inconsistency in the field of ATR-DDR.

### Reassociation of the Rad9 tail inactivates the checkpoint through dissociation of Rad17 and Rhino

To our knowledge, this is the first study to present a structural model of the Rad9 C-terminal tail (P356–L370) bound to the Rad9 front pocket, providing a molecular explanation for previous biochemical findings (Fig. 7). Specifically, substitutions of Rad9 residues K359, K360, R362, L364, F365, and F366 were shown to abolish the interaction with the core-ring structure of the 9–1–1 complex [63, 64], and these residues were identified as major anchoring points in our structural analysis (Fig 7F–I; Table 5). These consistencies validate the predicted binding mode of the Rad9 tail.

The structural analysis of the Rad9 C-terminal tail suggests that it can competitively displace Rhino from the Rad9 front pocket. Rad9 P356–L370 binds to the same front pocket as Rhino, and its binding energy was comparable to those of Rhino P10–A31 and S83–S98 (Fig. 7; Table 12). The competition between the Rad9 tail and Rhino suggests a mechanistic role for the Rad9 tail in checkpoint deactivation, independent of the 9–1–1 polymer hypothesis. In the absence of polymer formation, the Rad9 tail can displace the first KYxxL+ motif, thereby disrupting the association of Rhino, an interaction essential for ATR-DDR [24, 25]. Under the 9–1–1 polymer hypothesis, reassociation of the Rad9 C-terminal tail competitively displaces the second KYxxL+ motif of Rhino, leading to polymer disassembly (Fig. 11H; Table 13). As the polymerized form is proposed to sustain checkpoint signaling by concentrating checkpoint mediators, checkpoint inactivation is conceptualized as the structural disassembly of the 9–1–1 polymer. Thus, Rad9 tail reassociation serves as a key trigger for checkpoint inactivation via Rhino dissociation, regardless of the 9–1–1 polymer hypothesis.

The reassociation of the Rad9 tail with the core-ring structure also promotes the release of Rad17 (Fig. 11H) [6, 19]. Our structural analysis predicts that the Rad9 S291–T313 segment binds to the same cleft on Hus1 that interacts with the Rad17 iVERGE motif and RAD24 C-terminal region (Fig. 8). Rad9 S291–T313 and Rad17-iVERGE showed similar binding energies (Table 12), suggesting that these peptides are structurally and energetically interchangeable. Since Rad17 contributes to the recruitment of downstream checkpoint effectors [78], displacement of Rad17 by the Rad9 tail provides a mechanism for inactivating ATR-DDR. The replacement reactions of Rhino and Rad17 did not significantly alter the total binding energies of the ligands for the 9–1–1 complex (Fig. 11I, columns G and H), suggesting that the checkpoint complex shifts from a maintenance phase to an inactivated state through energetically neutral remodeling events.

The disengagement of Rad17 and Rhino functionally substitutes for the unloading of the 9–1–1 complex. Although clamp unloaders have been identified for PCNA [79–81], no such unloader has been identified for the 9–1–1 complex. In this context, our findings suggest that the Rad9 tail acts as a functional substitute for a dedicated unloader by competitively replacing Rhino and the Rad17 iVERGE motif. This replacement triggers the dissociation of checkpoint mediators and the inactivation of ATR-DDR, even if the 9–1–1 complex remains chromatin-bound as a structural memory of prior DNA damage.

## Conclusion

Our findings establish a semi-quantitative model of Rad17–9–1–1–Rhino complex dynamics, in which the complex undergoes structural remodeling through transitions among energetically equivalent states. Central to this remodeling are the conserved KYxxL+ motifs, which mediate context-specific interactions that orchestrate the dynamic assembly, transition, and disassembly of the checkpoint complexes. These interactions support the 9–1–1 polymer hypothesis and help resolve the long-standing discrepancy regarding the Rad17 and Rad9 foci. Together, these results provide a framework for investigating how structural remodeling of checkpoint complexes governs the maintenance and inactivation of ATR-DDR.

## Supplementary Material

gkag093_Supplemental_Files

## Data Availability

The data underlying this article are available in the article and in its online Supplementary data.
