# Supplementary material for: Energetically equivalent structural transitions in the Rad17–Rad9–Hus1–Rad1–Rhino complex underlie the sequential progression from activation through maintenance to inactivation of the ATR-dependent DNA damage response
Source: Nucleic Acids Res. 2026 Feb 16;54(4):gkag093. doi: 10.1093/nar/gkag093 (PMC12907562; doi:10.1093/nar/gkag093)
Supplement: gkag093_Supplemental_Files [file gkag093_supplemental_files.zip › Rhino, supplementary Figures, 251130 0413, noRireki BC.pdf]

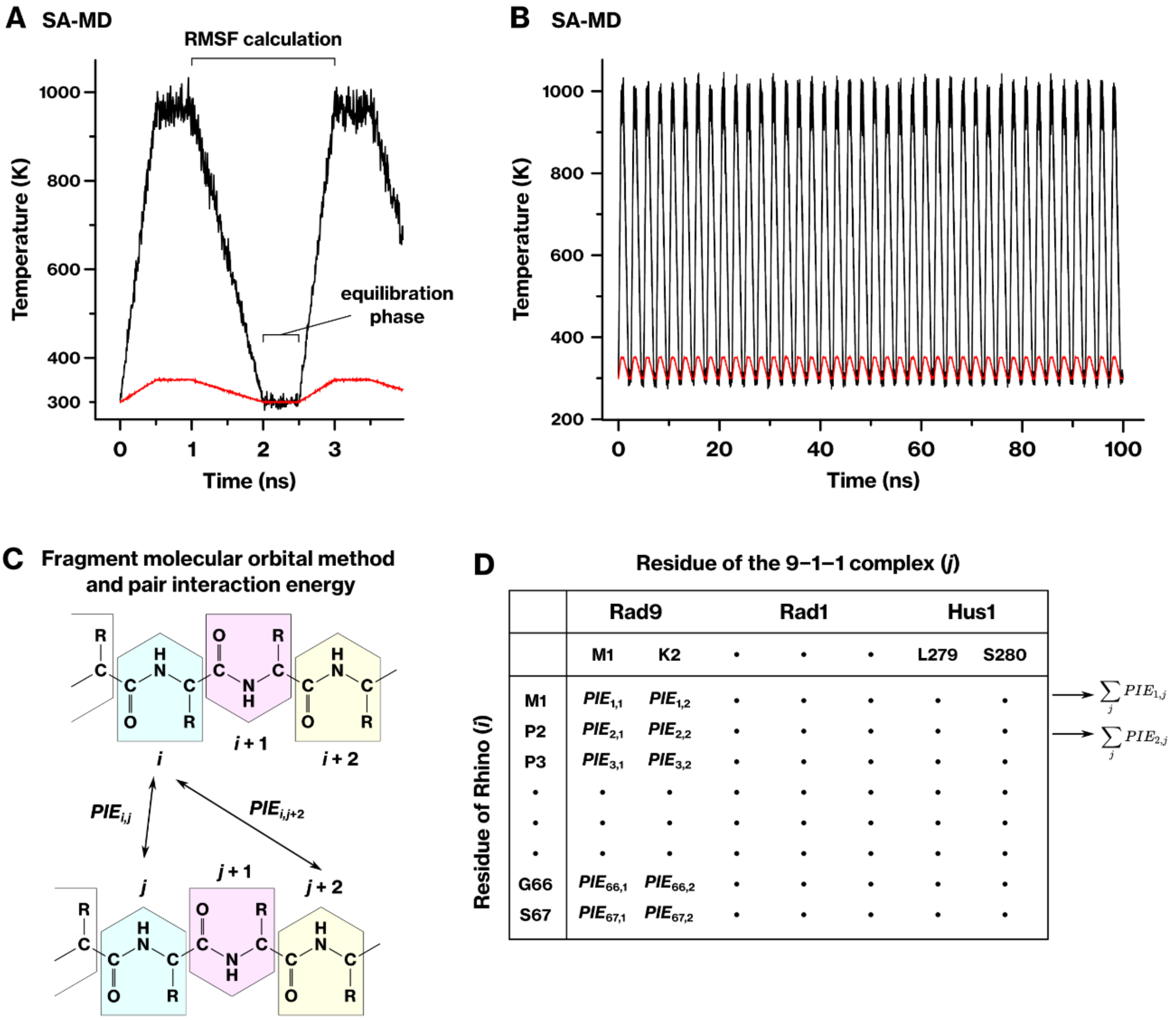

Figure S1. Conformational sampling using SA-MD simulation, the FMO method, and PIE analysis (related to Figures 1A–C). (A, B) Temperature profiles from the SA-MD simulation are shown. Binding conformations of Rhino on the 9–1–1 complex were analyzed following the procedure described in Figure 1C. Possible binding conformations were sampled using SA-MD simulation. Rhino (ligand) and the 9–1–1 complex (receptor) were assigned to high-temperature (black line) and low-temperature (red line) groups, respectively. The averaged structure was obtained from the equilibration phase at 300 K. The RMSF of each residue was calculated from a 2000-ps trajectory centered on the equilibration phase. **(C, D)** Fragment molecular orbitals were defined as indicated by the pentagons. Using the FMO2-DFTB3/PCM method, $PIE_{i,j}$ values were calculated between the fragment molecular orbitals of individual residues in the ligand peptide (indexed by *i*) and those of the receptor (indexed by *j*), resulting in a matrix of PIE values.

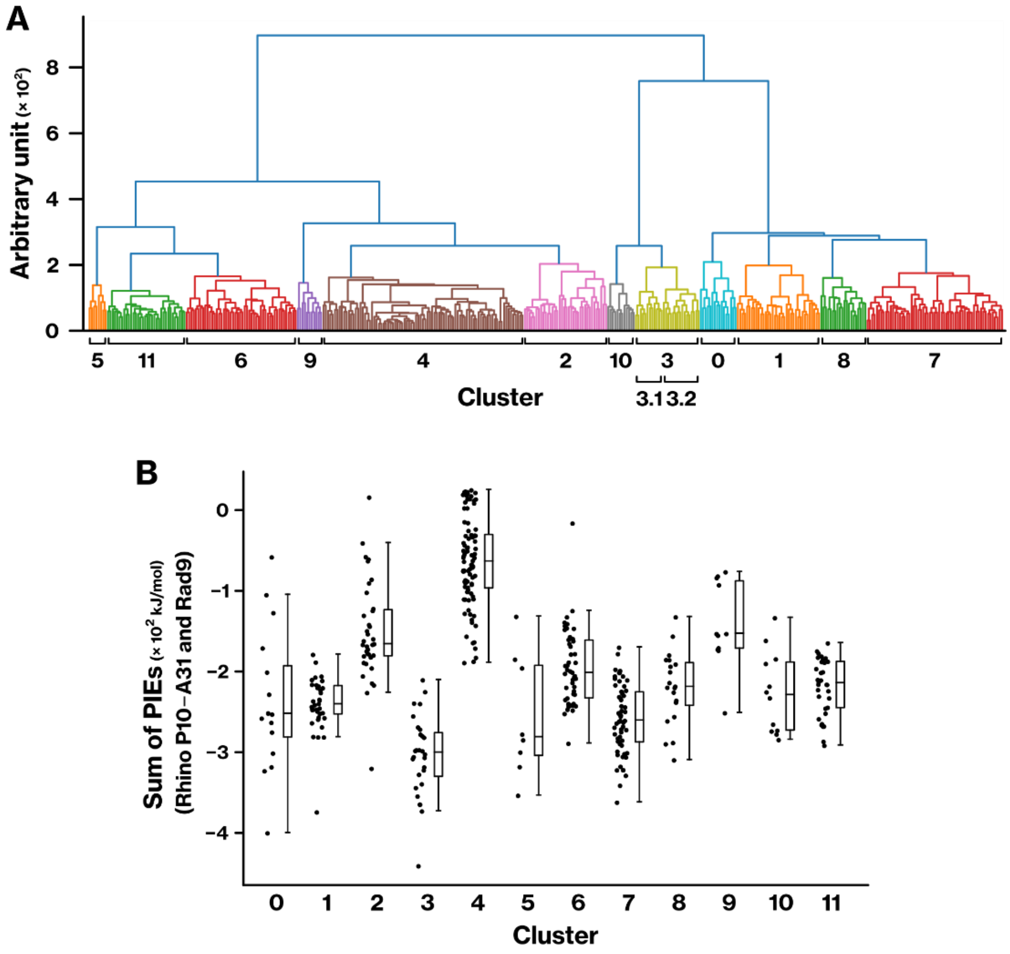

C

Null hypothesis rejection (upper triangle); *p*-value of Tukey–Kramer test (lower triangle)

	0	1	2	3	4	5	6	7	8	9	10	11
0		F	T	T	T	F	F	F	F	T	F	F
1	1.000		T	T	T	F	T	F	F	T	F	F
2	< 0.001	< 0.001		T	T	T	T	T	T	F	T	T
3	0.002	< 0.001	< 0.001		T	F	T	T	T	T	T	T
4	< 0.001	< 0.001	< 0.001	< 0.001		T	T	T	T	T	T	T
5	0.999	1.000	< 0.001	0.450	< 0.001		F	F	F	T	F	F
6	0.200	0.005	0.006	< 0.001	< 0.001	0.092		T	F	F	F	F
7	0.931	0.869	< 0.001	0.008	< 0.001	1.000	< 0.001		F	T	F	T
8	0.998	0.960	< 0.001	< 0.001	< 0.001	0.882	0.820	0.134		T	F	F
9	< 0.001	< 0.001	1.000	< 0.001	< 0.001	< 0.001	0.086	< 0.001	0.004		T	T
10	1.000	1.000	< 0.001	0.001	< 0.001	0.985	0.769	0.021	1.000	0.005		F
11	0.994	0.867	< 0.001	< 0.001	< 0.001	0.815	0.638	1.000	1.000	0.001	1.000	

Figure S2. Identification of a stable binding conformation of Rhino P10–A31 on Rad9 (related to Figure 1D). (A) The conformations sampled in Figure S1 were grouped into clusters using agglomerative clustering based on their PIE matrices. **(B)** The sums of PIE values for individual conformations, $\sum_i \sum_j PIE_{i,j}$, were calculated and plotted as single data points, grouped by cluster. A stable cluster was identified based on the total PIE values. **(C)** The distribution of PIE sums in each cluster was analyzed using the Tukey–Kramer test. Rejection of the null hypothesis and the corresponding *p*-values are shown for each pair. T, true; F, false.

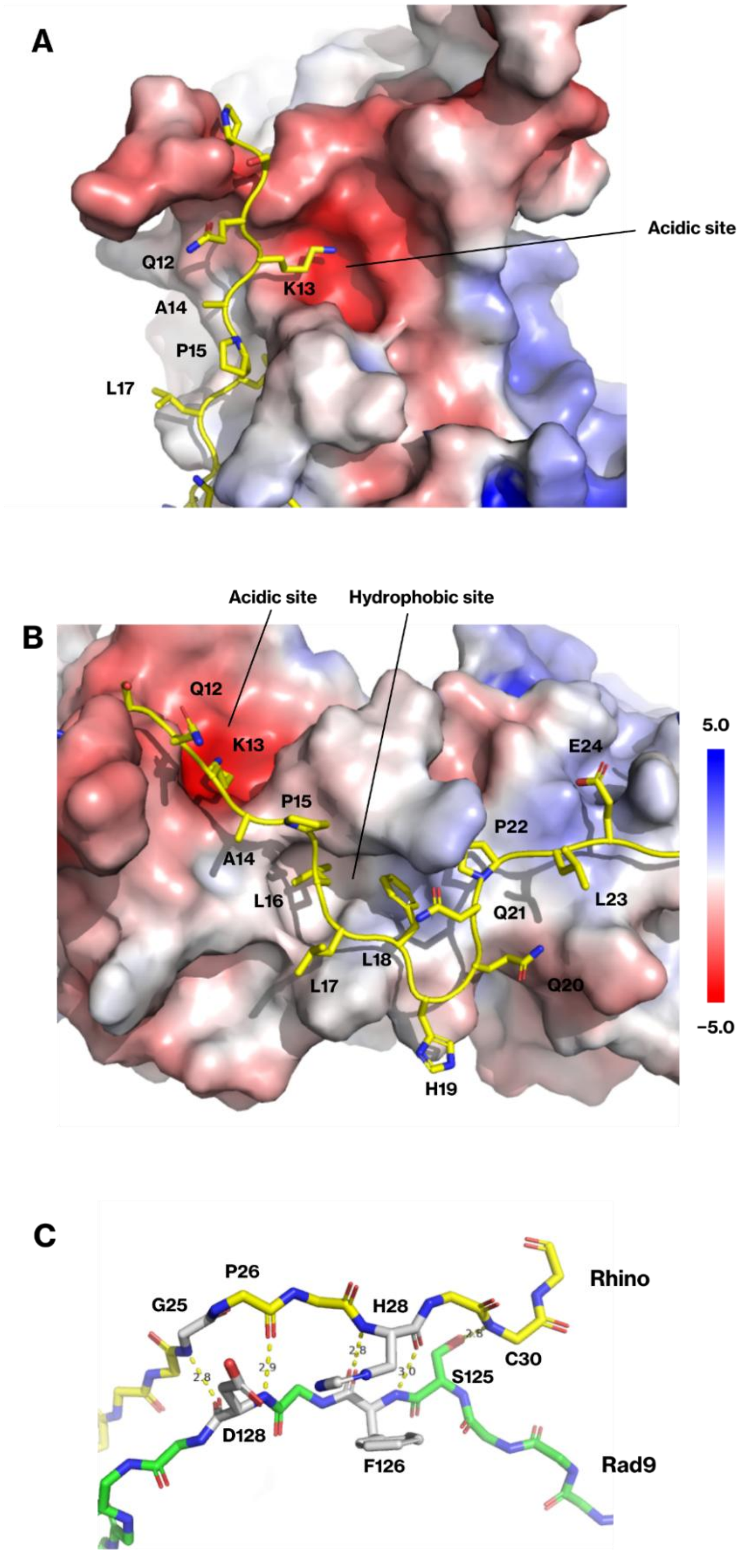

Figure S3. Association of Rhino P10–A31 with the front pocket of Rad9 (related to Figures 1H–J).

(A, B) Uncropped versions of Figures 1H and 1I are shown. **(C)** Hydrogen bonds between Rhino residues G25–C30 and the IDL loop of Rad9 are shown. The corresponding cartoon representation is provided in Figure 1J.

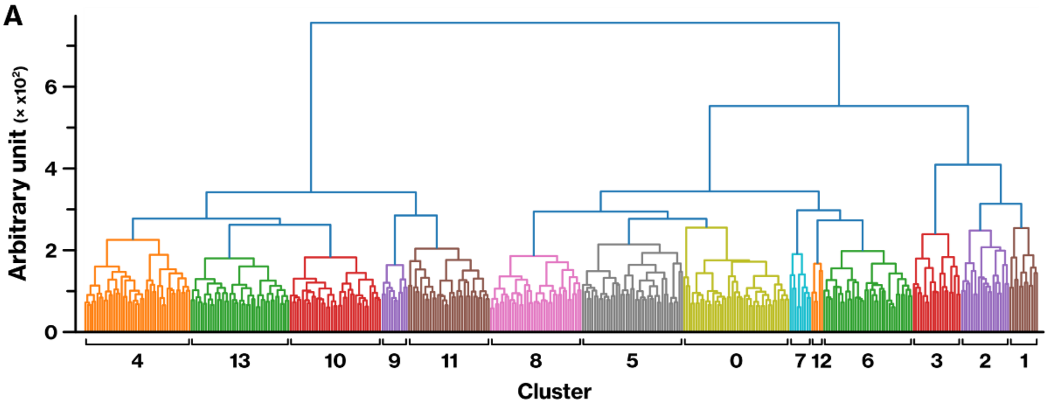

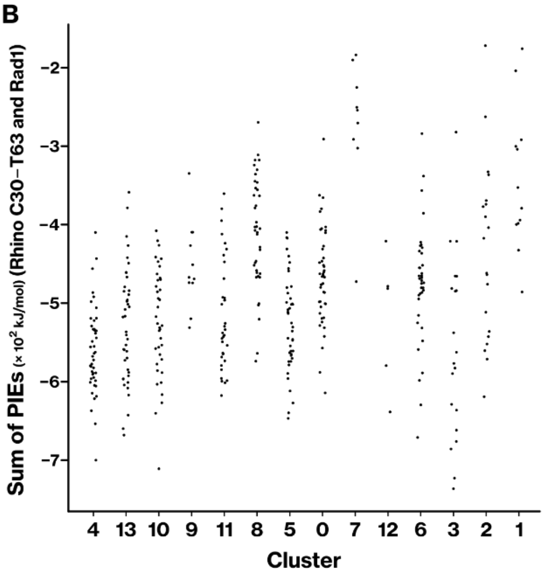

C

Cluster	PIE (kJ/mol)
4	−560 ± 56
3	−555 ± 117
13	−529 ± 74
5	−525 ± 61
10	−524 ± 69
12	−520 ± 88
11	−516 ± 70
6	−476 ± 75
0	−466 ± 62
9	−451 ± 55
2	−436 ± 114
8	−410 ± 72
1	−343 ± 92
7	−271 ± 86

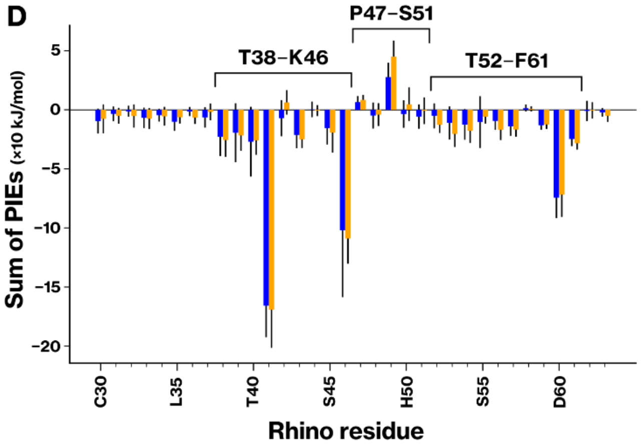

Figure S4. Rhino interacts with Rad1 through two regions: T38–K46 and T52–F61 (related to Figure 2A). **(A)** The trajectory of the SA-MD simulation shown in Figure 1B was reanalyzed based on PIEs between Rhino residues C30–T63 and Rad1, and the sampled conformations were grouped into clusters. **(B)** The sums of PIE values were plotted for each cluster, and stable clusters were identified. **(C)** The mean and standard deviation of PIEs are shown for each cluster. **(D)** The sum of PIE values for each Rhino residue was calculated for each conformation, and the mean and standard deviation per cluster were plotted for clusters 3 (blue) and 4 (orange). Thick and thin bars represent the mean and standard deviation, respectively. Rhino residues T38–K46, P47–S51, and T52–F61 are indicated.

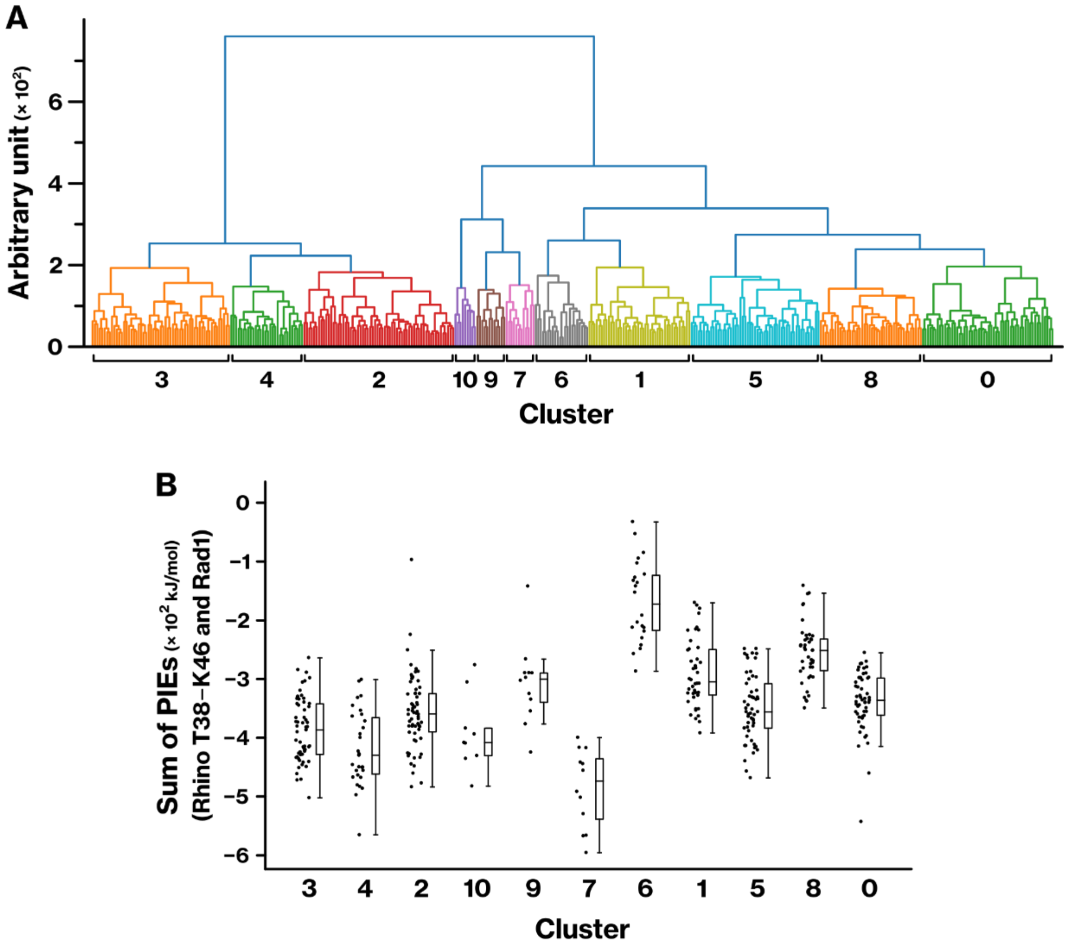

C

Null hypothesis rejection (upper triangle); *p*-value of Tukey–Kramer test (lower triangle)

	0	1	2	3	4	5	6	7	8	9	10
0		T	F	T	T	F	T	T	T	F	F
1	0.004		T	T	T	T	T	T	F	F	T
2	0.833	< 0.001		F	T	F	T	T	T	F	F
3	< 0.001	< 0.001	0.160		F	T	T	T	T	T	F
4	< 0.001	< 0.001	< 0.001	0.360		T	T	T	T	T	F
5	0.997	< 0.001	1.000	0.030	< 0.001		T	T	T	F	F
6	< 0.001	< 0.001	< 0.001	< 0.001	< 0.001	< 0.001		T	T	T	T
7	< 0.001	< 0.001	< 0.001	< 0.001	0.038	< 0.001	< 0.001		T	T	T
8	< 0.001	0.220	< 0.001	< 0.001	< 0.001	< 0.001	< 0.001	< 0.001		F	T
9	0.874	0.998	0.230	0.001	< 0.001	0.517	< 0.001	< 0.001	0.203		T
10	0.288	< 0.001	0.850	1.000	0.980	0.630	< 0.001	0.014	< 0.001	0.050	

Figure S5. Identification of a stable binding conformation of Rhino T38–K46 on Rad1 (related to Figures 2B–E). (A) The trajectory of the SA-MD simulation in Figure 1B was reanalyzed based on PIEs between Rhino residues T38–K46 and Rad1. **(B, C)** The sums of PIE values were plotted for each cluster. The statistical significance of differences between clusters was assessed using the Tukey–Kramer test.

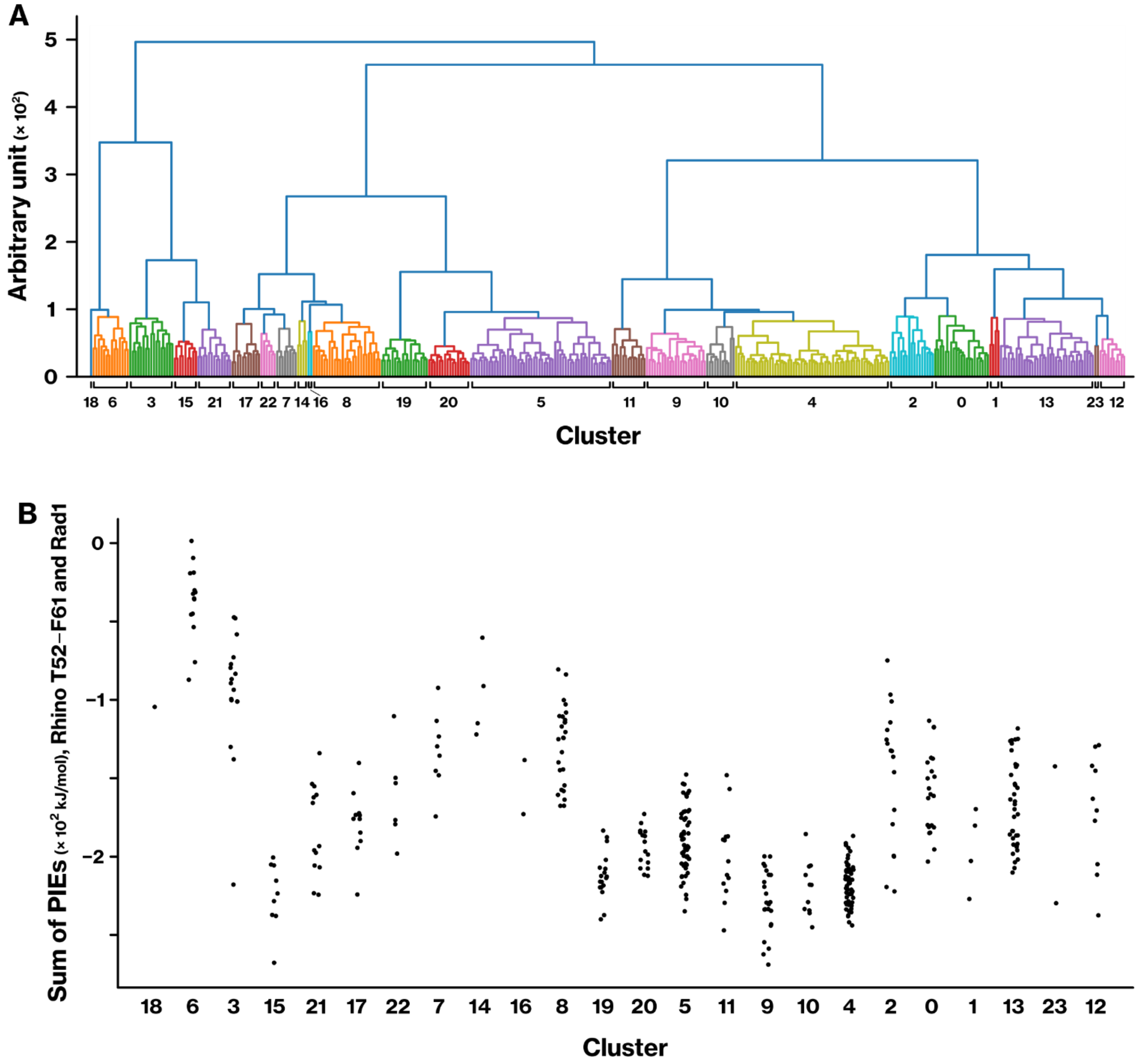

Figure S6. Identification of a stable binding conformation of Rhino T52–F61 on Rad1 (related to Figures 2F–J). (A, B) The same analysis as in Figure S5 was performed, except that PIEs for Rhino residues T52–F61 were analyzed.

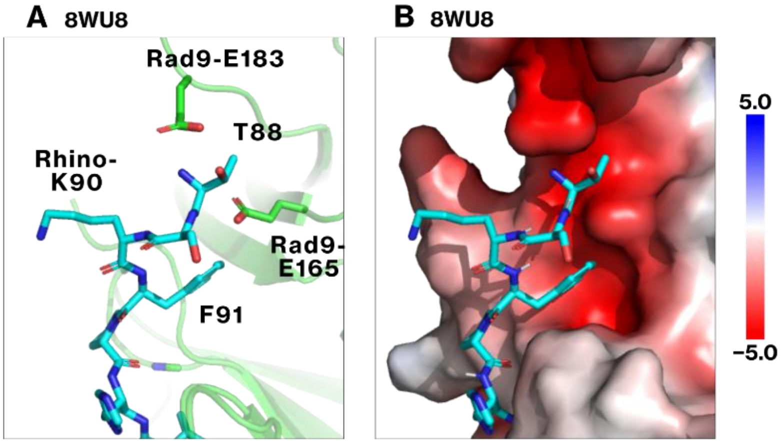

Figure S7. Conformational analysis of Rhino S83–S104 on Rad9 (related to Figure 3).

(A, B) The conformation of Rhino residues T88–P99 on the Rad9 front pocket in PDB 8WU8 is shown. Panel B depicts the side chains of Rhino-T88, K90, and F91, as well as the van der Waals surface of Rad9.

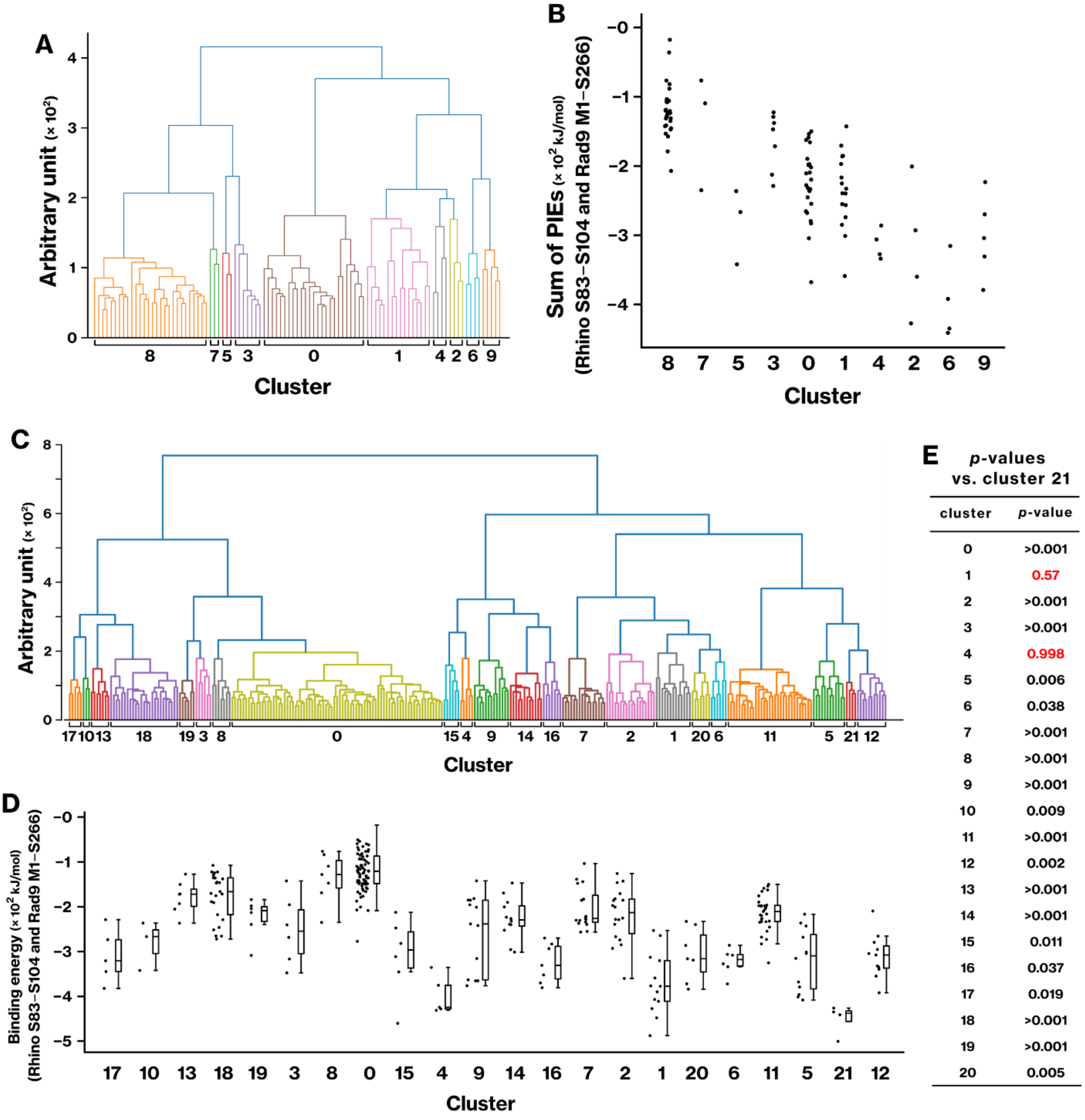

cluster	*p*-value
0	>0.001
1	0.57
2	>0.001
3	>0.001
4	0.998
5	0.006
6	0.038
7	>0.001
8	>0.001
9	>0.001
10	0.009
11	>0.001
12	0.002
13	>0.001
14	>0.001
15	0.011
16	0.037
17	0.019
18	>0.001
19	>0.001
20	0.005

Figure S8. Conformational analysis of Rhino S83–S104 on Rad9 (related to Figure 3B).

(A, B) Possible binding conformations of Rhino residues S83–S104 on the Rad9 core-ring structure were sampled using SA-MD simulation. The initial structure was generated using AlphaFold2. The sampled conformations were clustered based on PIEs between Rhino S83–S104 and Rad9 M1–S266 (A). The sums of PIE values were plotted, and stable clusters were identified (B). **(C–E)** The most stable conformations from clusters 2 and 6 (identified in panels A and B) were used as starting models for another round of conformational sampling. The newly sampled conformations were combined with those from the initial SA-MD simulation shown in panels A and B. These were then re-clustered and visualized as a dendrogram (C). The sums of PIE values were plotted, and a stable cluster was identified (D). Statistical significance of the differences in total PIE values between cluster 21 and the other clusters was assessed using the Tukey–Kramer test (E).

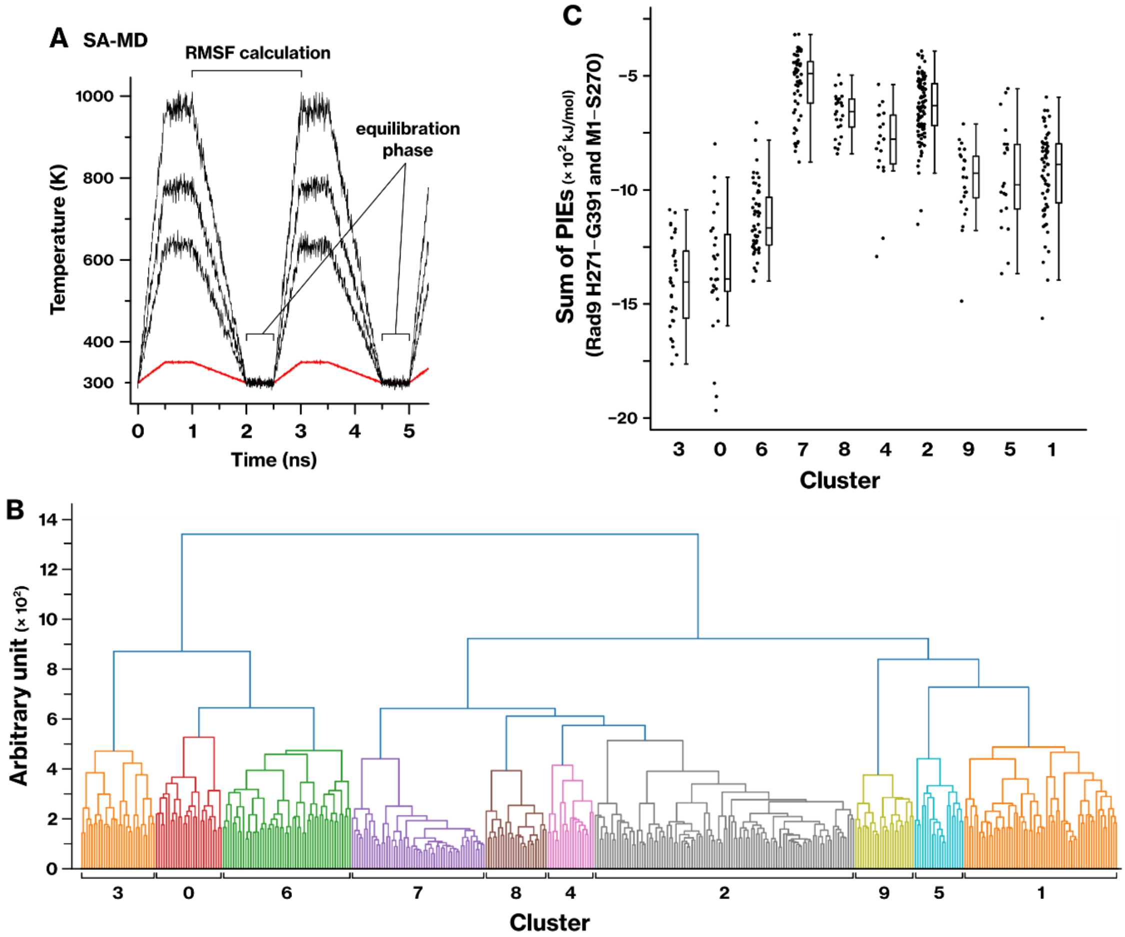

D

Null hypothesis rejection (upper triangle); *p*-value of Tukey–Kramer test (lower triangle)

	0	1	2	3	4	5	6	7	8	9
0		T	T	F	T	T	T	T	T	T
1	<0.001		T	T	F	F	T	T	T	F
2	<0.001	<0.001		T	T	T	T	T	F	T
3	0.984	<0.001	<0.001		T	T	T	T	T	T
4	<0.001	0.136	0.003	<0.001		F	T	T	F	F
5	<0.001	1.000	<0.001	<0.001	0.316		T	T	T	F
6	<0.001	<0.001	<0.001	<0.001	<0.001	0.001		T	T	T
7	<0.001	<0.001	0.021	<0.001	<0.001	<0.001	<0.001		T	T
8	<0.001	<0.001	1.000	<0.001	0.213	<0.001	<0.001	0.048		T
9	<0.001	1.000	<0.001	<0.001	0.097	1.000	0.002	<0.001	<0.001	

Figure S9. Conformation analysis of the Rad9 tail on the core-ring structure of the 9–1–1 complex (related to Figures 7A–C). (A) Possible binding conformations of Rad9 residues H271–G391 on a complex composed of Rad9 M1–S270, Hus1, and Rad1 were sampled using SA-MD simulation. The Rad9 tail (ligand) and the core-ring structure (receptor) were assigned to high- and low-temperature groups, respectively. Their temperature profiles are shown as the black (high) and red (low) lines. The high-temperature group used 1000 K, 800 K, or 650 K as the maximum temperature. **(B)** Averaged conformations were sampled from the trajectories of the SA-MD simulation shown in Figure 7A. PIE values were calculated using the FMO2-DFTB3/PCM method, and the conformations were clustered based on PIEs between Rad9 residues H271–G391 and M1–S270. **(C, D)** The sums of PIE values were plotted, and stable clusters were identified (C). Statistical significance of differences was assessed using the Tukey–Kramer test (D). T, true; F, false.

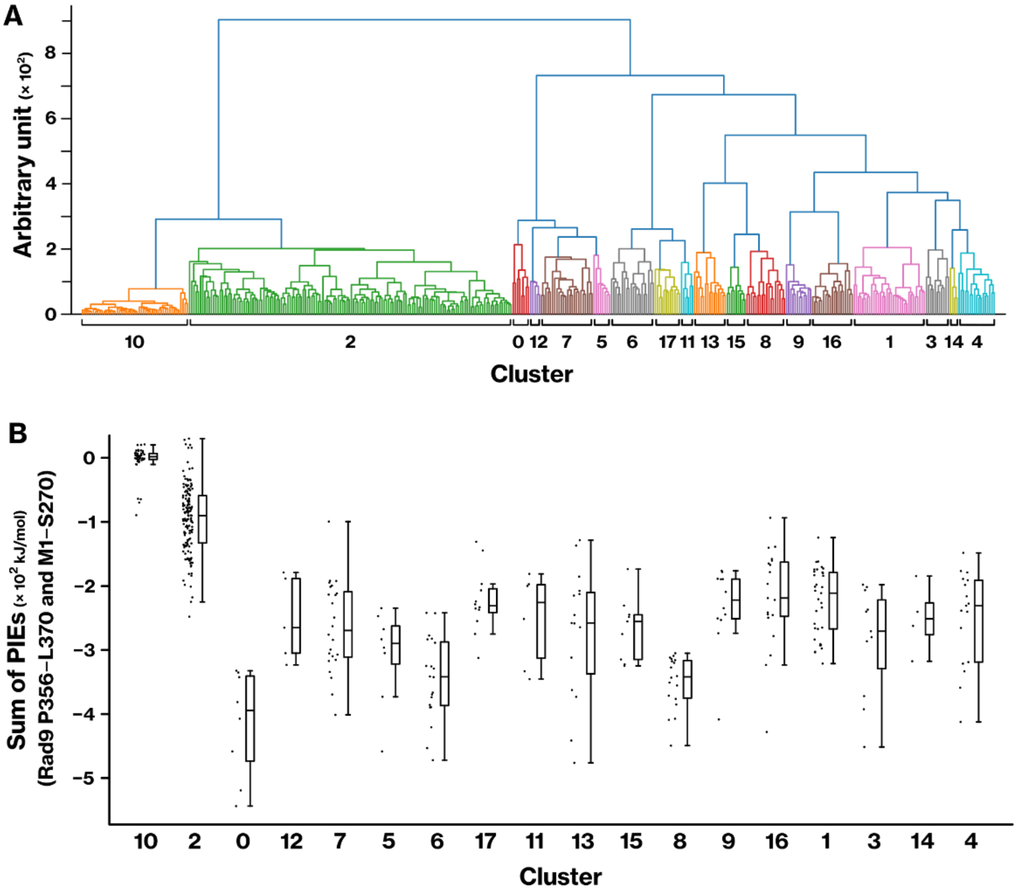

C

Null hypothesis rejection (upper triangle); *p*-value of Tukey–Kramer test (lower triangle)

	0	1	2	3	4	5	6	7	8	9	10	11	12	13	14	15	16	17
0		T	T	T	T	T	F	T	F	T	T	T	T	T	T	T	T	T
1	<0.001		T	F	F	T	T	F	T	F	T	F	F	F	F	F	F	F
2	<0.001	<0.001		T	T	T	T	T	T	T	T	T	T	T	T	T	T	T
3	<0.001	0.244	<0.001		F	F	F	F	F	F	T	F	F	F	F	F	F	F
4	<0.001	0.995	<0.001	0.987		F	T	F	T	F	T	F	F	F	F	F	F	F
5	0.035	0.041	<0.001	1.000	0.679		F	F	F	F	T	F	F	F	F	F	T	F
6	0.297	<0.001	<0.001	0.382	<0.001	0.991		T	F	T	T	F	F	T	F	F	T	T
7	<0.001	0.594	<0.001	1.000	1.000	0.925	<0.001		T	F	T	F	F	F	F	F	F	F
8	0.480	<0.001	<0.001	0.260	<0.001	0.967	1.000	<0.001		T	T	T	F	T	F	T	T	T
9	<0.001	1.000	<0.001	0.808	1.000	0.326	<0.001	0.993	<0.001		T	F	F	F	F	F	F	F
10	<0.001	<0.001	<0.001	<0.001	<0.001	<0.001	<0.001	<0.001	<0.001	<0.001		T	T	T	T	T	T	T
11	<0.001	1.000	<0.001	1.000	1.000	0.949	0.069	1.000	0.043	1.000	<0.001		F	F	F	F	F	F
12	<0.001	1.000	<0.001	1.000	1.000	0.972	0.143	1.000	0.095	1.000	<0.001	1.000		F	F	F	F	F
13	<0.001	0.497	<0.001	1.000	1.000	0.995	0.034	1.000	0.018	0.967	<0.001	1.000	1.000		F	F	F	F
14	0.001	1.000	<0.001	1.000	1.000	0.984	0.257	1.000	0.185	1.000	<0.001	1.000	1.000	1.000		F	F	F
15	<0.001	0.935	<0.001	1.000	1.000	0.991	0.081	1.000	0.048	0.999	<0.001	1.000	1.000	1.000	1.000		F	F
16	<0.001	1.000	<0.001	0.223	0.984	0.039	<0.001	0.565	<0.001	1.000	<0.001	0.999	1.000	0.456	1.000	0.887		F
17	<0.001	1.000	<0.001	0.526	0.999	0.143	<0.001	0.904	<0.001	1.000	<0.001	1.000	1.000	0.803	1.000	0.978	1.000	

Figure S10. Identification of a stable binding conformation of Rad9 P356–L370 on the core-ring structure: first round of conformational sampling (related to Figure 7D). (A) The conformations sampled in Figures 7A and S10 were reanalyzed based on the PIEs between Rad9 residues P356–L370 and M1–S270, and were grouped into clusters. **(B, C)** The sums of PIE values were plotted, and stable clusters were identified (B). Statistical significance of differences was assessed using the Tukey–Kramer test (C). T, true; F, false.

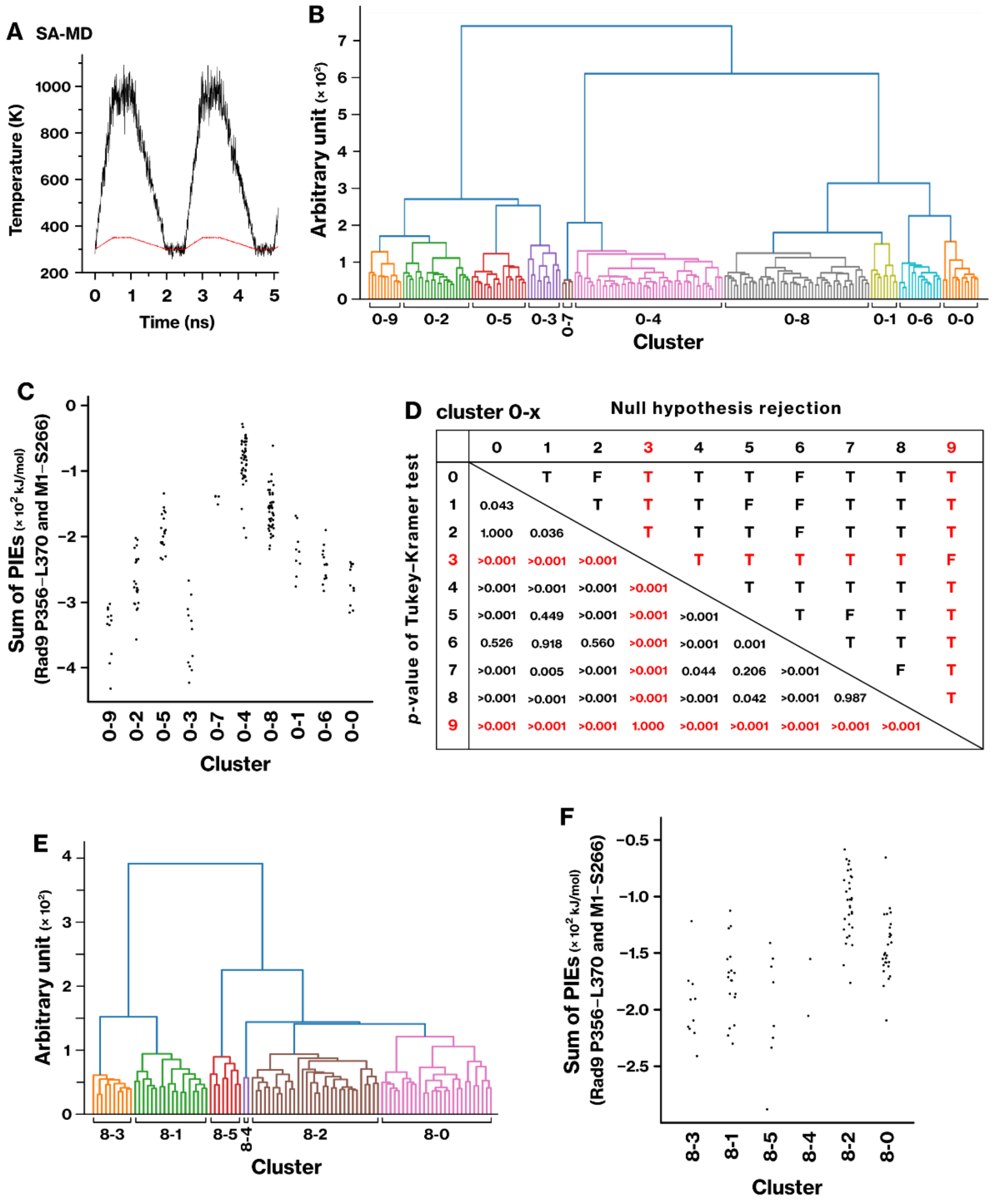

	0	1	2	3	4	5	6	7	8	9
0		T	F	T	T	T	F	T	T	T
1	0.043		T	T	T	F	F	T	T	T
2	1.000	0.036		T	T	T	F	T	T	T
3	>0.001	>0.001	>0.001		T	T	T	T	T	F
4	>0.001	>0.001	>0.001	>0.001		T	T	T	T	T
5	>0.001	0.449	>0.001	>0.001	>0.001		T	F	T	T
6	0.526	0.918	0.560	>0.001	>0.001	0.001		T	T	T
7	>0.001	0.005	>0.001	>0.001	0.044	0.206	>0.001		F	T
8	>0.001	>0.001	>0.001	>0.001	>0.001	0.042	>0.001	0.987		T
9	>0.001	>0.001	>0.001	1.000	>0.001	>0.001	>0.001	>0.001	>0.001	

Figure S11. Conformational sampling of Rad9 P356–L370 on the core-ring structure: second round of sampling (related to Figure 7D). (A) The most stable conformation from cluster 0 in Figure S10 was used as the starting model for the SA-MD simulation. The Rad9 tail (ligand) and the core-ring structure (receptor) were assigned to the high- and low-temperature groups, respectively. Their temperature profiles are shown as black (high) and red (low) lines. The high-temperature group used 1000 K as the maximum temperature. **(B–D)** Possible binding conformations were sampled, and PIEs between Rad9 residues P356–L370 and M1–S266 were calculated using the FMO2-DFTB3/PCM method. Based on the resulting PIE matrices, the sampled conformations were clustered using agglomerative clustering, and stable clusters were identified (B). The sums of PIE values were plotted (C), and statistical significance of differences was assessed using the Tukey–Kramer test. T, true; F, false (D). **(E, F)** The same conformational sampling procedure as in panels A–D was performed, except that cluster 8 in Figure S10 was used as the starting model.

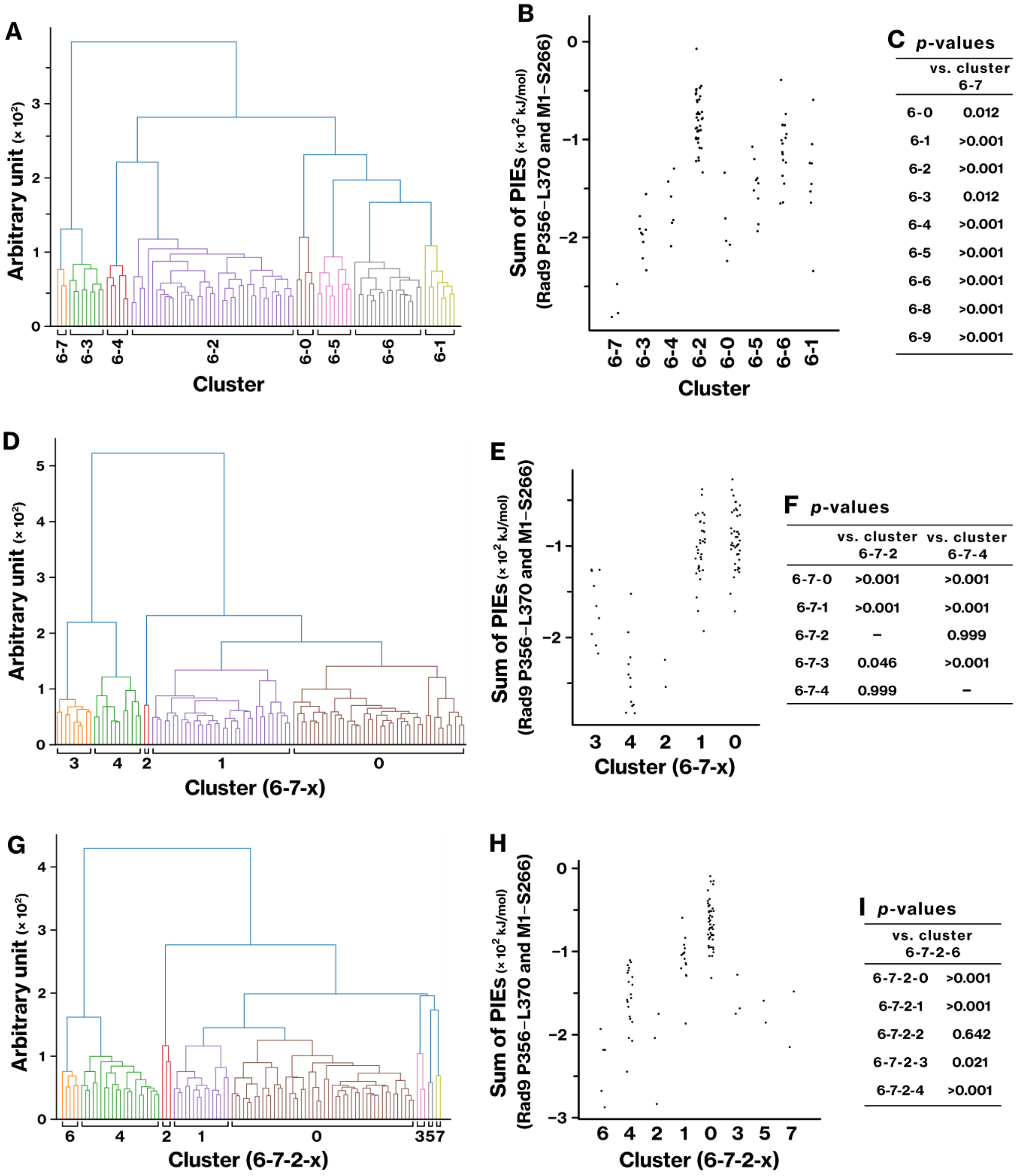

C *p*-values

	vs. cluster 6-7
6-0	0.012
6-1	>0.001
6-2	>0.001
6-3	0.012
6-4	>0.001
6-5	>0.001
6-6	>0.001
6-8	>0.001
6-9	>0.001

F *p*-values

	vs. cluster 6-7-2	vs. cluster 6-7-4
6-7-0	>0.001	>0.001
6-7-1	>0.001	>0.001
6-7-2	–	0.999
6-7-3	0.046	>0.001
6-7-4	0.999	–

I *p*-values

	vs. cluster 6-7-2-6
6-7-2-0	>0.001
6-7-2-1	>0.001
6-7-2-2	0.642
6-7-2-3	0.021
6-7-2-4	>0.001

Figure S12. Conformational sampling of Rad9 residues P356–L370 on the core-ring structure: second, third, and fourth rounds of sampling (related to Figure 7D). (A–C) The same analyses described in Figure S11A–D were performed, except that cluster 6 from Figure S10 served as the starting model. Conformations were sampled (A), and stable clusters were identified (B). Statistical significance of the differences in total PIE values between cluster 6-7 and the other clusters was assessed using the Tukey–Kramer test (C). **(D–F)** The same analyses were performed using cluster 6-7 from panels A–C as the starting model. Statistical significance of the differences in total PIE values was assessed using the Tukey–Kramer test for each of clusters 6-7-2 and 6-7-4 in comparison to the other clusters. **(G–I)** The same analyses were repeated using cluster 6-7-2 from panels D–F as the starting model. Statistical significance of the differences in total PIE values between cluster 6-7-2-6 and the other clusters was assessed using the Tukey–Kramer test. T, true; F, false.

A

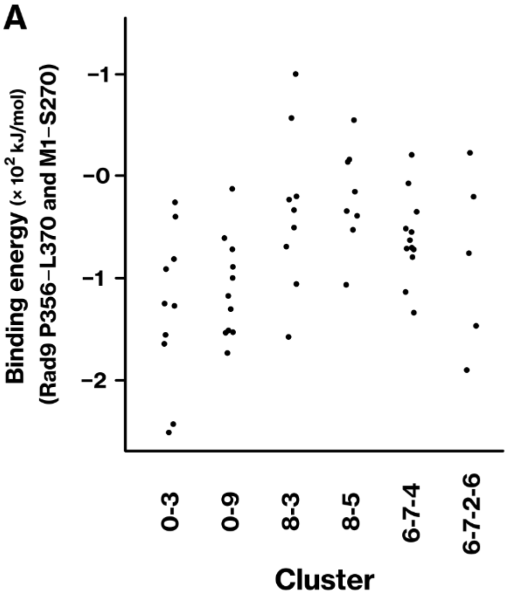

B

Cluster	Binding energy (kJ/mol)
0-3	−131 ± 76
0-9	−111 ± 49
8-3	−34 ± 78
8-5	−21 ± 50
6-7-4	−61 ± 42
6-7-2-6	−83 ± 88

C Null hypothesis rejection (upper triangle); *p*-value of Tukey–Kramer test (lower triangle)

	0-3	0-9	8-3	8-5	6-7-4	6-7-2-6
0-3		F	T	T	F	F
0-9	0.976		F	T	F	F
8-3	0.017	0.088		F	F	F
8-5	0.007	0.036	0.998		F	F
6-7-4	0.117	0.419	0.919	0.715		F
6-7-2-6	0.718	0.959	0.734	0.520	0.988	

D Cluster 0-9

Rad9 residue	PIE (kJ/mol)	*E*es (kJ/mol)	*E*disp (kJ/mol)	*G*sol (kJ/mol)
K359	−128 ± 30	−279 ± 26	−5 ± 2	156 ± 30
K360	−178 ± 37	−317 ± 31	−11 ± 2	151 ± 19
F361	10 ± 17	10 ± 9	−6 ± 3	5 ± 6
**R362**	**−209** ± 21	−293 ± 16	**−13** ± 2	95 ± 14
S363	−3 ± 13	1 ± 7	−3 ± 1	−1 ± 7
**L364**	**−16** ± 9	3 ± 3	**−15** ± 1	**−5** ± 7
F365	−8 ± 6	−1 ± 4	−7 ± 3	0 ± 4
F366	−3 ± 8	−4 ± 5	−6 ± 5	7 ± 9

E Cluster 6-7-4

Rad9 residue	PIE (kJ/mol)	*E*es (kJ/mol)	*E*disp (kJ/mol)	*G*sol (kJ/mol)
K359	−160 ± 34	−239 ± 36	−7 ± 2	86 ± 18
K360	−54 ± 55	−131 ± 66	−3 ± 3	80 ± 26
**F361**	**−14** ± 8	5 ± 9	**−16** ± 2	**−3** ± 5
R362	−91 ± 66	−154 ± 54	−9 ± 5	71 ± 18
S363	−2 ± 6	3 ± 4	−4 ± 1	−1 ± 5
**L364**	**−15** ± 6	1 ± 5	**−13** ± 1	**−4** ± 3
F365	−3 ± 5	2 ± 3	−5 ± 4	0 ± 3
**F366**	**−13** ± 8	−1 ± 2	**−12** ± 5	**−1** ± 2

Figure S13. Identification of stable conformations of Rad9 P356–L370 on the Rad9 front pocket (related to Figures 7F–I and Table 6). (A–C) The binding energy (ΔG_{bind}), defined as $G_{complex} - (G_{protein} + G_{ligand})$, between Rad9 residues P356–L370 and M1–S270 was calculated for each conformation using the FMO3-DFTB3/PCM3 method. The resulting binding energies were plotted for clusters 0-3, 0-9, 8-3, 8-5, 6-7-4, and 6-7-2-6 identified in Figures S11 and S12 (A), and are shown as the mean ± standard deviation (B). Statistical significance of differences in the binding energies was assessed using the Tukey–Kramer test. T, true; F, false (C). **(D, E)** The same analysis as in Table 6 was performed, except that clusters 0-9 and 6-7-4 were used. Sums of PIE, *E*es, *E*disp, and *G*sol were calculated for each Rad9 residue from K359 to F366, and the mean and standard deviation were computed across conformations for each residue.

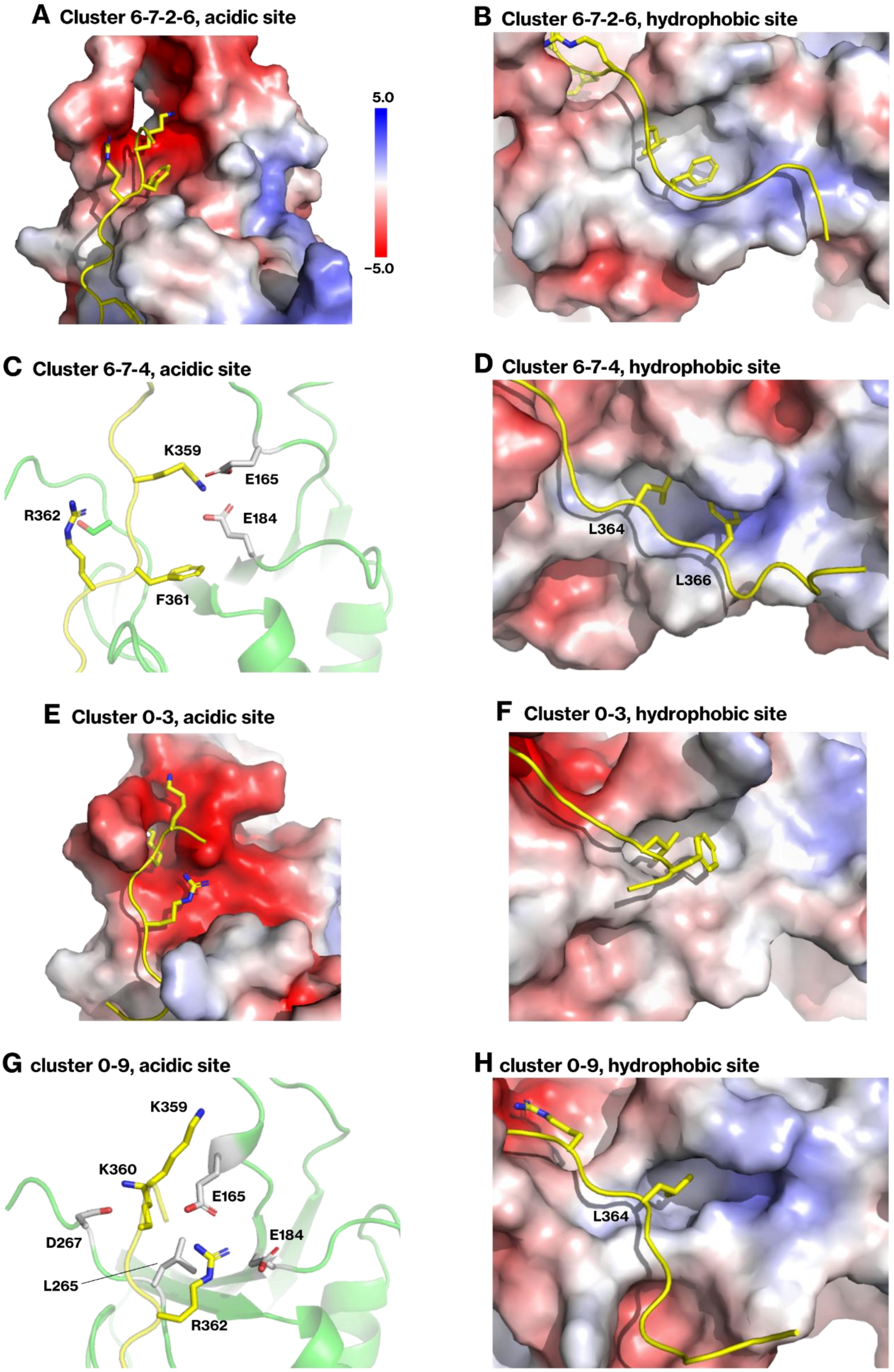

Figure S14. Association of Rad9 P356–L370 with the front pocket of Rad9 (related to Figures 7J–O). **(A, B, E, F)** Uncropped versions of Figures 7J–M are shown. **(C, D, G, H)** Binding conformations of Rad9 residues P356–L370 at the front pocket of Rad9 are shown for clusters 6-7-4 and 0-9.

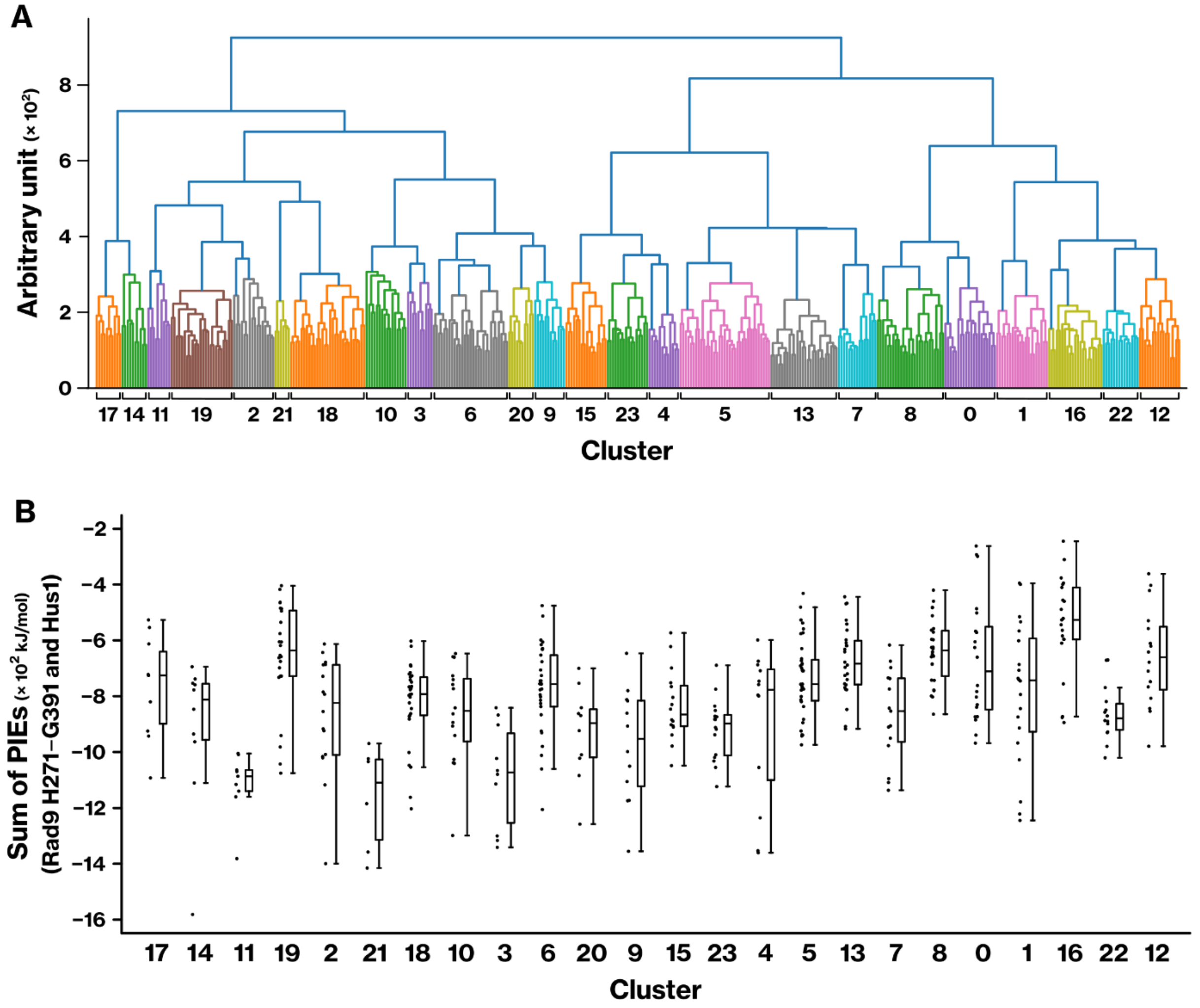

Figure S15. Identification of stable conformations of Rad9 H271–G391 on Hus1 (related to Figure 8A).

(A, B) The conformations sampled in Figure 7A were reanalyzed based on PIE values between Rad9 residues H271–G391 and Hus1, and were grouped into clusters (A). The sums of PIE values were plotted to identify stable clusters (B).

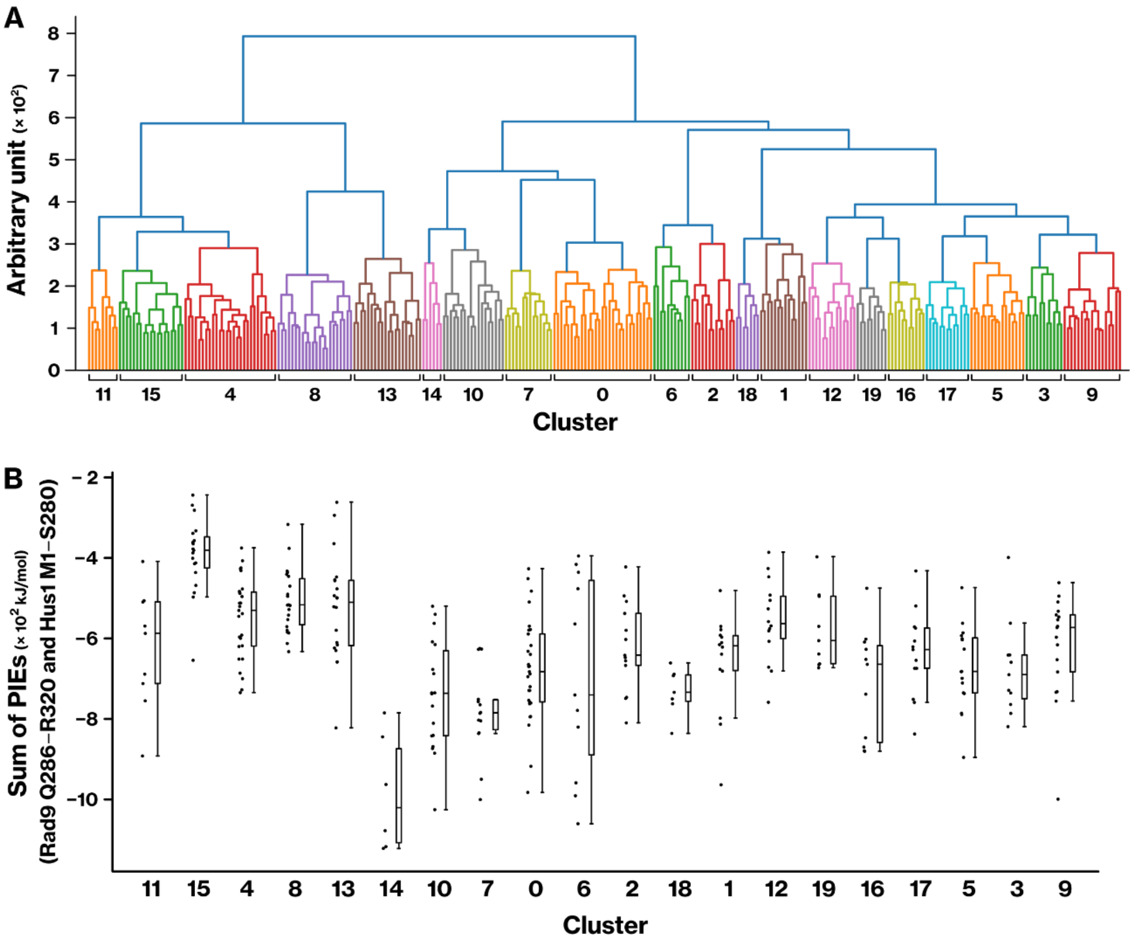

C *p*-values vs. cluster 14 in Tukey-Kramer test

cluster	*p*-value	cluster	*p*-value
0	>0.001	10	0.005
1	>0.001	11	>0.001
2	>0.001	12	>0.001
3	>0.001	13	>0.001
4	>0.001	15	>0.001
5	>0.001	16	0.003
6	>0.001	17	>0.001
7	0.088	18	0.033
8	>0.001	19	>0.001
9	>0.001		

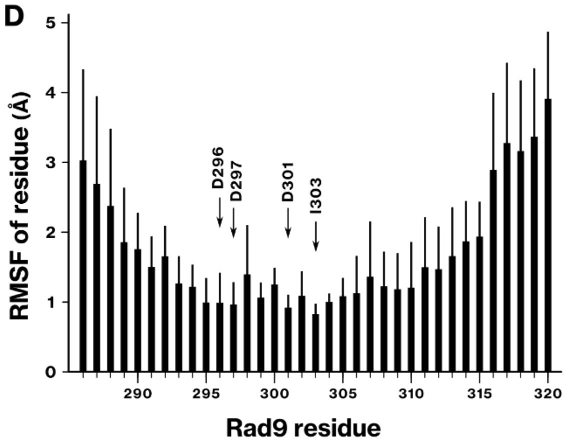

Figure S16. Conformational sampling of Rad9 Q286–R320 on Hus1: second round of sampling (related to Figure 8A). (A–C) The most stable conformations from clusters 3, 11, and 21 in Figure S15 were used as the starting models, and possible binding conformations were sampled using SA-MD simulation. PIE values between Rad9 residues Q286–R320 and Hus1 were calculated, and the sampled conformations were clustered (A). The sums of PIE values were plotted to identify stable clusters (B). Statistical significance of the differences in total PIE values between cluster 14 and the other clusters was assessed using the Tukey–Kramer test (C). **(D)** RMSF was calculated for each Rad9 residue (Q286–R320) in each conformation and then averaged within cluster 14. Thick and thin bars represent the mean and standard deviation, respectively.

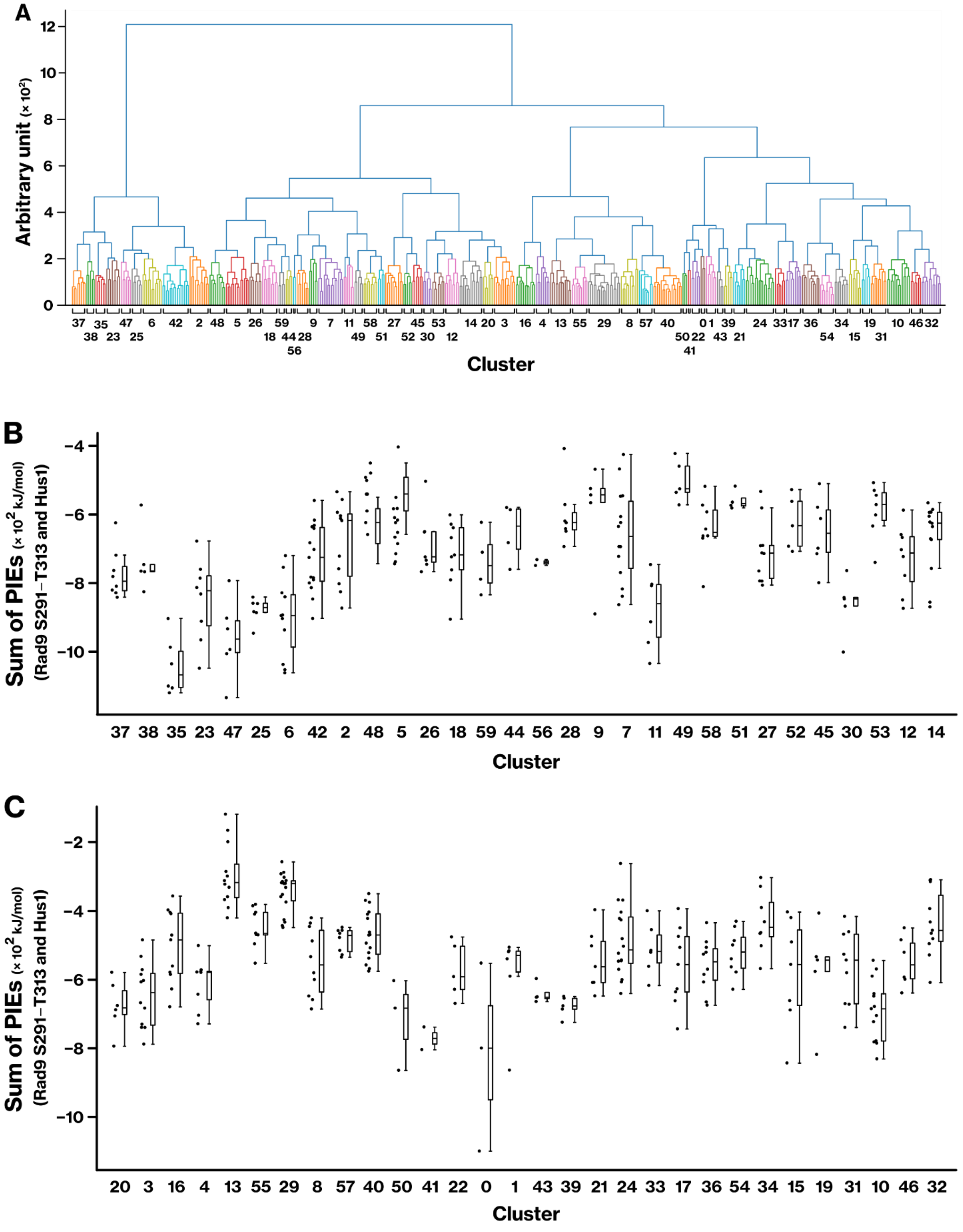

Figure S17. Conformational sampling of Rad9 S291–T313 on Hus1: third round of sampling (related to Figure 8B). (A–C) The most stable conformation from cluster 14 in Figure S16 was used as the starting model, and additional binding conformations were sampled using SA-MD simulation. The PIE values between Rad9 residues S291–T313 and Hus1 were calculated, and the resulting conformations were clustered (A). The sums of PIE values were plotted to identify stable clusters (B, C).

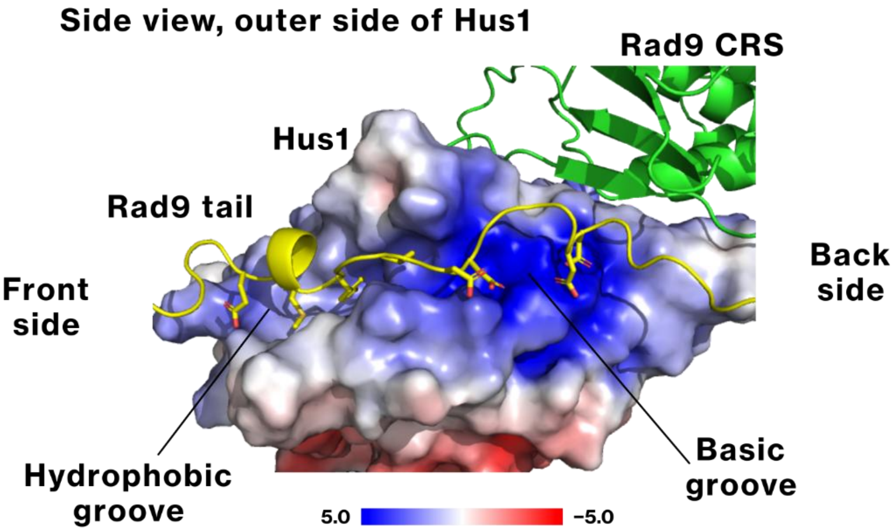

Figure S18. Association of Rad9 residues S291–T313 with the basic and hydrophobic grooves of Hus1 (related to Figures 8F and 8G). The most stable conformation from cluster 35, identified in Figure 8B, is shown. In the Rad9 C-terminal tail, the same side chains highlighted in Figures 8F and 8G are depicted in yellow. The Rad9 core-ring structure is also shown in green. The van der Waals surface of Hus1 is rendered with an overlaid electrostatic potential map.

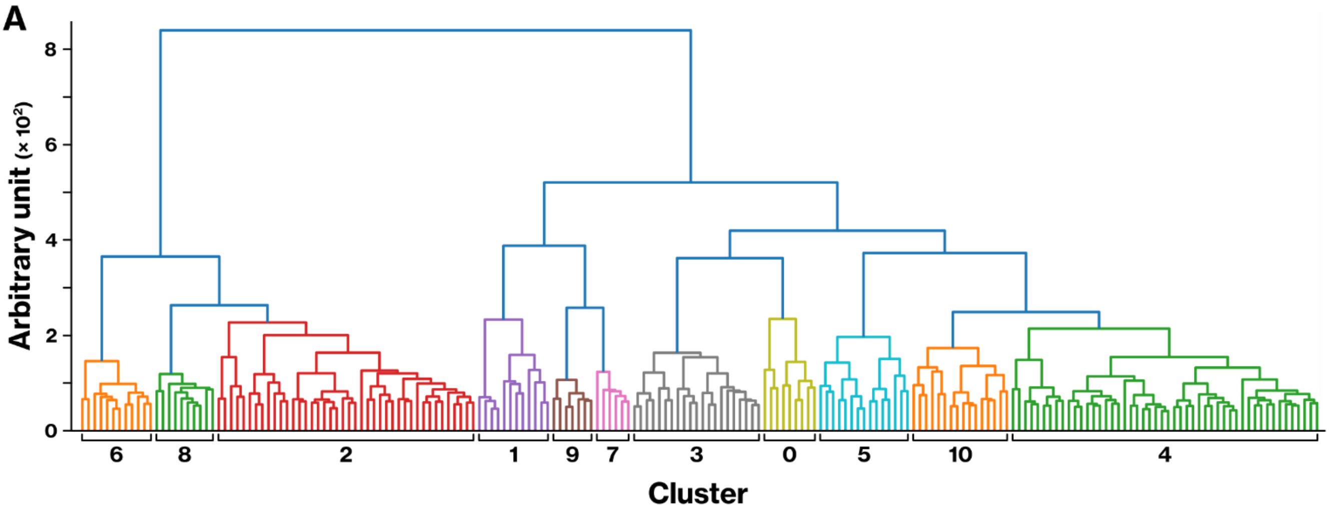

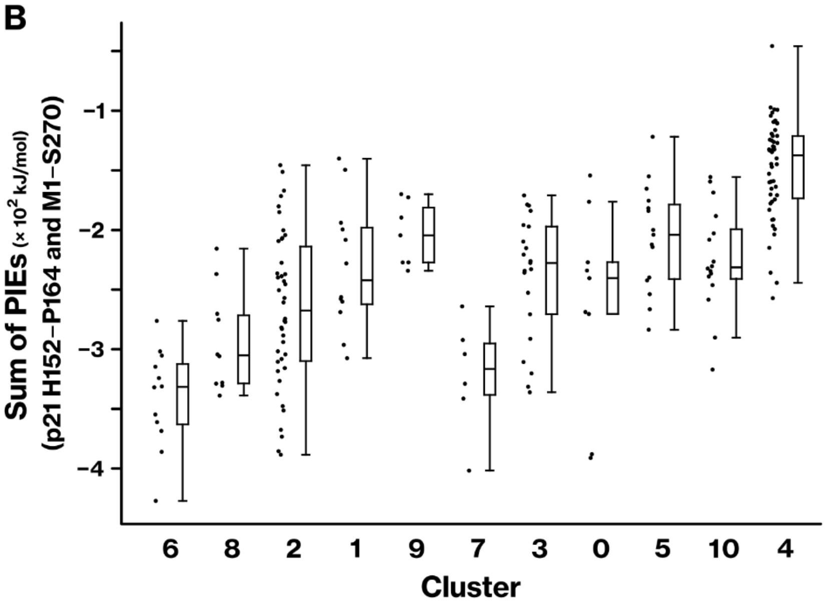

C ***p*-values vs. cluster 6**

cluster	*p*-value
0	0.027
1	>0.001
2	0.001
3	>0.001
4	>0.001
5	>0.001
7	1.000
8	0.570
9	>0.001
10	>0.001

Figure S19. Identification of stable conformations of p21 H152–P164 at the Rad9 front pocket (related to Figure 9). (A–C) Possible binding conformations of p21 residues H152–P164 on the core-ring structure were sampled using SA-MD simulation. The initial structure was generated using AlphaFold2. The sampled conformations were grouped into clusters based on PIE values between p21 H152–P164 and Rad9 M1–S270 (A). The sums of PIE values were plotted to identify stable clusters (B). Statistical significance of the differences in total PIE values between cluster 6 and the other clusters was assessed using the Tukey–Kramer test (C).
