# Supplementary material for: Energetically equivalent structural transitions in the Rad17–Rad9–Hus1–Rad1–Rhino complex underlie the sequential progression from activation through maintenance to inactivation of the ATR-dependent DNA damage response
Source: Nucleic Acids Res. 2026 Feb 16;54(4):gkag093. doi: 10.1093/nar/gkag093 (PMC12907562; doi:10.1093/nar/gkag093)
Supplement: gkag093_Supplemental_Files [file gkag093_supplemental_files.zip › Rhino, supplementary results and PDB lists, 251207 2222, noRireki BC.pdf]

Supplementary Results

Supplementary Results 1. Binding conformations of Rhino residues T38–K46 and T52–F61 on Rad1

Possible binding conformations of Rhino T38–K46 on Rad1 were analyzed using SA-MD simulation, the FMO method, and PIE analysis. These analyses identified cluster 7 in Figure 2B as the most stable (Figures S5A–C). In cluster 7, Rhino-V43 showed lower RMSF and interacted with a hydrophobic pocket on Rad1 (Figures 2C, E). Rhino-V43 showed a solvation energy (*G*sol) of −16.4 kJ/mol and a dispersion energy (*E*disp) of −10.5 kJ/mol (Table 2). These energies suggest that Rhino-V43 stabilizes the interaction between Rhino and Rad1 through hydrophobic and van der Waals interactions. Rhino-R41 showed the largest interaction energy and lower RMSF. The interaction was mostly electrostatic (Figure 2C; Table 2). Rhino residues H39, T40, and R41 interacted with the C-terminal region of Rad1 (Figure 2E). The precise binding mode varied within cluster 7 because of the flexibility of the Rad1 C-terminal region. Together, these results revealed the optimal binding conformation of Rhino T38–K46 on the front side of Rad1.

To gain further insight into the binding mode of Rhino T52–F61, the trajectories of the SA-MD simulation were reanalyzed. Among the sampled conformations, cluster 9, which included the initial structure, was selected as the most stable based on PIE analysis (Figures S6A, B). The initial structure had a total PIE of –205 kJ/mol, whereas the most stable conformation in cluster 9 exhibited stronger binding energy (Figure S6B), indicating that further structural optimization occurred during the SA-MD simulation. In cluster 9, the spatial distribution of interaction energy revealed that Rhino T52–F61 comprised three subregions that contributed to interactions with Rad1: D60/F61, W56/V57, and T52/I53/T54 (Figure 2H). These results revealed the stable binding conformation of Rhino T52–F61 on the outer side of Rad1.

Supplementary Results 2. Sampling of the binding conformations of Rad9 residues P356–L370 on the front pocket of Rad9

AlphaFold2 predicted that the Rad9 C-terminal tail interacts with both Rad9 and Hus1. All five models consistently predicted that the Rad9 tail binds to the front pocket of Rad9. However, structural variations were observed in side chain orientations among the models.

We performed structural optimization of the predicted model, in which the Rad9 tail is bound to the Rad9 front pocket, as detailed below. We performed SA-MD simulation and selected Rad9 residues P357–L364 for further optimization based on their RMSF, as described in the *Results* section (*In silico analysis of the association of the Rad9 C-terminal tail to the Rad9 front pocket*). We reanalyzed the MD trajectories using PIE matrices calculated between Rad9 residues P356–L370 and M1–S270, which resulted in 18 conformational clusters (Figure S10A). Cluster 0 was identified as the most stable cluster (Figure S10B), while clusters 6 and 8 were statistically indistinguishable from cluster 0, as determined by the Tukey–Kramer test (Figure S10C). These three clusters were selected for subsequent analysis.

To further refine and identify the most stable and representative conformations, we performed iterative structure optimization (Figure 7D). Initial structures were derived from the most stable conformations in clusters 0, 6, and 8, treating Rad9 residues P356–L370 as the ligand. In the second round of sampling initiated from cluster 0, PIE analysis identified clusters 0-3 and 0-9 as the most stable (Figures S11A–C). These two clusters were grouped and found to be statistically distinct from the other clusters according to the Tukey–Kramer test (Figure S11D). In the second sampling initiated from cluster 8, clusters 8-3 and 8-5 were identified as stable clusters (Figures S11E, F). In the second sampling initiated from cluster 6, PIE analysis identified cluster 6-7 as the most stable (Figures S12A, B). This cluster was classified as a distinct group from the others according to the Tukey–Kramer test (Figure S12C). Taken together, these SA-MD simulations identified clusters 0-3, 0-9, 8-3, 8-5, and 6-7 as newly optimized and stable conformations.

We performed further optimization on cluster 6-7 by repeating the SA-MD simulation. Cluster 6-7 adopted a conformation distinct from those of the other clusters. However, it comprised only three structures (Figure S12B). In the third round of sampling from cluster 6-7, clusters 6-7-4 and 6-7-2 were identified as the most stable (Figures S12D, E) and were classified as a distinct group according to the Tukey–Kramer test (Figure S12F). Cluster 6-7-2 exhibited a conformation distinct from that of cluster 6-7-4. However, it contained only two structures (Figure S12E), which prompted another round of conformational sampling. In the fourth round of sampling from cluster 6-7-2, cluster 6-7-2-6 was identified as the most stable (Figures S12G, H). Clusters 6-7-2-5 and 6-7-2-7 were excluded from the statistical analysis because of their limited size (only two conformations each). Cluster 6-7-2-2 was excluded from further analysis because of its heterogeneous conformations. As a result of this iterative process, clusters 6-7-4 and 6-7-2-6 were ultimately identified as the most stable

conformations.

To evaluate the validity of each candidate structure prior to further analysis, binding energies were calculated based on the conformations within six selected clusters: 0-3, 0-9, 8-3, 8-5, 6-7-4, and 6-7-2-6. The binding free energy (ΔG_{bind}) was calculated as $\Delta G_{\mathrm{bind}} = G_{\mathrm{complex}} - (G_{\mathrm{receptor}} + G_{\mathrm{ligand}})$, using Rad9 residues M1–S270 and P356–L370 as the receptor and ligand, respectively. Among them, cluster 0-3 showed the strongest binding energy (Figures S13A, B) and was statistically indistinguishable from clusters 0-9, 6-7-4, and 6-7-2-6 according to the Tukey–Kramer test (Figure S13C). In contrast, clusters 8-3 and 8-5 showed less favorable binding energies and were classified into a different group (Figures S13B, C). Based on these results, clusters 8-3 and 8-5 were excluded from further analysis, and clusters 0-3, 0-9, 6-7-4, and 6-7-2-6 were retained for subsequent evaluations.

Supplementary Results 3. Conformational sampling of the Rad9 C-terminal tail in complex with the basic and hydrophobic grooves of Hus1

The association of the Rad9 C-terminal tail with Hus1 was analyzed as shown in Figure 8A. We reanalyzed the trajectory of the SA-MD simulation involving full-length Rad9, Hus1, and Rad1. This is the same trajectory used for the analysis of the interaction between the Rad9 tail and the front pocket (Figure 7A). We identified clusters 3, 11, and 21 as the most stable, based on the lowest median values of the total PIE between Hus1 and the Rad9 tail (H271–G391), indicating stronger binding energies and interactions (Figure S15A, B). The initial structure belonged to cluster 16, indicating that the SA-MD simulation sampled more energetically favorable conformations over time. In clusters 3, 11, and 21, Hus1 interacted with the Rad9 residues H294–I314, H290–E317, and S291–I314, respectively. Based on these observations, we selected Rad9 residues Q286–R320, which span the interacting regions in clusters 3, 11, and 21, for further structural and energetic analysis.

To refine the binding conformation, we conducted a second round of SA-MD simulations, using clusters 3, 11, and 21 as the starting structures. Among the resulting clusters, cluster 14 exhibited the strongest interaction energy (Figures S16A, B). Statistical analysis supported this result: the Tukey–Kramer test showed that cluster 14 was significantly different from all other clusters, except for cluster 7 (Figure S16C). Additionally, a Student's *t*-test further revealed a significant difference between cluster 14 and cluster 7 ($p = 0.003$). We therefore selected cluster 14 as the initial structure in the next round of sampling. RMSF analysis of cluster 14 revealed increased flexibility in the peripheral

regions (Figure S16D), and Rad9 residues S291–T313, corresponding to the stable core region, were selected for further analysis.

We performed a third round of conformational sampling using cluster 14 as the initial structure. The resulting trajectory was analyzed based on the PIEs between Hus1 and Rad9 S291–T313, and cluster 35 was identified as the most stable (Figures S17B, C). Statistical analysis revealed that cluster 35 was significantly different from the other stable clusters, except for cluster 47 (Figure 8B). However, both clusters belonged to the same branch in the dendrogram (Figure S17A), indicating structural similarity. Notably, the initial structure of the third sampling was part of cluster 35, indicating that the most stable conformation had already been sampled during the second round. Taken together, these analyses identified the most stable conformation adopted by the Rad9 C-terminal tail on Hus1.

Supplementary PDB files

Supplementary PDB files contain the following complex structures.

Supplementary PDB file 1: Rhino P10–A31 and Rad9 M1–S270, visualized in Figure 1.

Supplementary PDB file 2: Rhino T38–I48 and Rad1 D12–S282, visualized in Figure 2.

Supplementary PDB file 3: Rhino T52–F61 and Rad1 M1–S282, visualized in Figure 2.

Supplementary PDB file 4: Rhino S83–S98 and Rad9 M1–S270, visualized in Figure 3.

Supplementary PDB file 5: Rad9 T355–A371 and M1–S270, cluster 6-7-2-6, visualized in Figure 7.

Supplementary PDB file 6: Rad9 T355–A371 and M1–S270, cluster 0-3, visualized in Figure 7.

Supplementary PDB file 7: Rad9 S291–T313 and Hus1 M1–S280, visualized in Figure 8.

Supplementary PDB file 8: p21 H152–P164 and Rad9 M1–S270, cluster 6, visualized in Figure 9.

Supplementary PDB file 9: p21 H152–P164 and Rad9 M1–S270, cluster 7, visualized in Figure 9.

Supplementary PDB file 10: 9–1–1 complex and Rhino R8–S67, shown in Figure 5.

Supplementary PDB file 11: 9–1–1 complex and Rhino S83–S98, shown in Figure 5.

Supplementary PDB file 12: two 9–1–1 complex connected via Rhino M1–S104, shown in Figure 6.

Supplementary PDB file 13: two 9–1–1 complex connected via Rhino R41–S104, shown in Figure 6.

Table S1. Reliability scores of AlphaFold2 predictions

Input sequence	ipTM+pTM	Related Figure
Rad9 M1–S270, Hus1, Rad1, Rhino M1–S238	0.783	Figure 1
Rad9 M1–S270, Hus1, Rad1, Rhino F61–M140	0.820	Figure 3
Rad9 M1–G391, Hus1, Rad1	0.844	Figure 7
Rad9 M1–S270, Hus1, Rad1, p21 H152–P164	0.879	Figure 9

The predicted template modelling (ipTM + pTM) score is shown for each structure predicted by AlphaFold2.
